# The effects of integrated care: a systematic review of UK and international evidence

**DOI:** 10.1186/s12913-018-3161-3

**Published:** 2018-05-10

**Authors:** Susan Baxter, Maxine Johnson, Duncan Chambers, Anthea Sutton, Elizabeth Goyder, Andrew Booth

**Affiliations:** School of Health and Related Research, University of Sheffield, Regent Court, Regent Street, Sheffield, S14DA UK

**Keywords:** Systematic review, Integrated care, Service reconfiguration, service delivery

## Abstract

**Background:**

Healthcare systems around the world have been responding to the demand for better integrated models of service delivery. However, there is a need for further clarity regarding the effects of these new models of integration, and exploration regarding whether models introduced in other care systems may achieve similar outcomes in a UK national health service context.

**Methods:**

The study aimed to carry out a systematic review of the effects of integration or co-ordination between healthcare services, or between health and social care on service delivery outcomes including effectiveness, efficiency and quality of care. Electronic databases including MEDLINE; Embase; PsycINFO; CINAHL; Science and Social Science Citation Indices; and the Cochrane Library were searched for relevant literature published between 2006 to March 2017. Online sources were searched for UK grey literature, and citation searching, and manual reference list screening were also carried out. Quantitative primary studies and systematic reviews, reporting actual or perceived effects on service delivery following the introduction of models of integration or co-ordination, in healthcare or health and social care settings in developed countries were eligible for inclusion. Strength of evidence for each outcome reported was analysed and synthesised using a four point comparative rating system of stronger, weaker, inconsistent or limited evidence.

**Results:**

One hundred sixty seven studies were eligible for inclusion. Analysis indicated evidence of perceived improved quality of care, evidence of increased patient satisfaction, and evidence of improved access to care. Evidence was rated as either inconsistent or limited regarding all other outcomes reported, including system-wide impacts on primary care, secondary care, and health care costs. There were limited differences between outcomes reported by UK and international studies, and overall the literature had a limited consideration of effects on service users.

**Conclusions:**

Models of integrated care may enhance patient satisfaction, increase perceived quality of care, and enable access to services, although the evidence for other outcomes including service costs remains unclear. Indications of improved access may have important implications for services struggling to cope with increasing demand.

**Trial registration:**

Prospero registration number: 42016037725.

**Electronic supplementary material:**

The online version of this article (10.1186/s12913-018-3161-3) contains supplementary material, which is available to authorized users.

## Background

It has been argued that growing financial and service pressures in the UK National Health Service (NHS) cannot be tackled without transforming how health and social care are delivered. The NHS Five Year Forward View Plan published in 2014 [1] sets out how services need to change, and emphasises the requirement for greater integration of care [2]. It is argued that increased service integration will enable the achievement of a financially sustainable health and social care system in the NHS by 2020. New models of integrated care are charged with achieving more care beyond the hospital walls, change in the size and shape of acute hospitals, and increased attention to prevention and population health [3]. The drive to introduce new models in the NHS has been formidable, with “vanguard” sites across England funded to test seven new care models that integrate services around the patient. Their impact is currently in the process of being evaluated.

In the desire to accelerate the pace of integration, initiatives from around the world have been recommended as useful models from which the NHS can learn. However, some authors have emphasised that it is imperative to consider contextual differences before implementing the same models in different services and location [4]. While it is important to learn from the international literature, positive outcomes reported in these international models may not be assumed in a UK setting, requiring careful scrutiny of potentially differing effects. There have been calls for greater clarity regarding precisely how integration may impact on outcomes [5]. Doubts regarding the ability of new models to deliver expected benefits have also recently been voiced, with a report from the National Audit Office concluding that progress towards integration has been slower and less successful than envisaged [6]. A systematic review published in 2017 examined initiatives to move care from hospitals to the community, and similarly concluded that anticipated cost savings could not be assumed [7].

In a landscape of changing service delivery and uncertainty regarding effectiveness of new models, we undertook a systematic review to examine the literature on outcomes of integrated care. Given the potential for learning from integrated models across the world, we aimed in particular to compare evidence from the UK and international literature, to explore where similarities and difference in effects have been reported. This paper focuses on data relating to the effects of models of integrated care on actual and perceived service delivery, including the efficiency, effectiveness and quality of care. Other findings from this study including factors influencing implementation and outcomes are reported elsewhere (Baxter et al. In Press).

## Methods

Highly complex system-wide interventions such as models of integrated care provide considerable challenges for systematic review methods [8]. Systematic reviews have typically sought clear intervention-outcome effects from “gold standard” randomised experimental studies. However, recent years have witnessed substantial growth in the range of review methods available, with recognition that different review types are appropriate for answering differing questions and purposes [9, 10]. We selected an appropriate review method to fulfil the three requirements of: examination of multiple types of integrated care initiatives and service delivery outcomes; inclusion of studies of varying designs across the hierarchy of evidence; and learning most applicable to the UK NHS context. We therefore adopted an approach drawing on work by Pawson, [11] which stresses that both rigour and relevance are important when scrutinising complex outcome patterns. We included studies of both comparator and non-comparator design from the UK (as these data were considered to privilege relevance), whereas we prioritised international systematic reviews and international primary studies with comparative design (thereby privileging rigour).

### Literature search strategy

The study protocol was registered with the PROSPERO database (number 42016037725) and was made available on the National Institute for Health Research website (available as an Additional file 1: Appendix S1) The review was conducted in line with PRISMA (Preferred Reporting Items for Systematic Reviews and Meta-Analyses) guidelines (Additional file 1: Appendix S2) [12].

The information specialist on the team carried out systematic searches of health, medical and social care databases in September 2016. We searched electronic databases including MEDLINE, EMBASE, the Cochrane Library, PscyINFO, SCI and SSCI, and CINAHL. Further details of the search strategy are available in the Additional file 1: Appendix S3. Other iterative searching techniques were also employed, including hand searching of reference lists of primary studies and other reviews. We searched for grey literature via reference lists and also via UK websites including that of the Kings Fund (https://www.kingsfund.org.uk) and NHS England (https://www.england.nhs.uk). In May 2017 we conducted a citation search to identify any literature published subsequent to the formal bibliographic searches.

### Eligibility criteria

We defined “models of integrated care” as changes to health or both health and health-related service delivery which aim to increase integration and/or coordination.

- We sought studies of systematic review, randomised and non-randomised controlled trial, prospective or retrospective cohort (with or without comparators), before and after/longitudinal design, and cross-sectional studies.
- We included studies reporting any outcome relating to the delivery of services (effectiveness or efficiency or quality) and/or the effect on patients and staff delivering services.
- Studies were required to have been carried out in a developed country (a member of the Organisation for Economic Collaboration and Development) and to have been published since 2006 in English, or have an English abstract. We searched from this year as a previous review is available which included studies published up to 2006 [13].

Studies were excluded if they reported only clinical, rather than service delivery outcomes, or if integrated services did not include healthcare. We included grey literature from the UK in the form of reports, but conference abstracts and theses were excluded.

### Data collection

Retrieved citations were uploaded to an EndNote database, and title and abstracts (where available) of papers were screened by three reviewers against the inclusion/exclusion criteria. Any queries regarding inclusion were discussed by the full team at regular (fortnightly) team meetings. After independent screening and discussion of the first 5% of the database to establish agreement, further screening was carried out by a single reviewer, with checking of a 10% sample by other team members.

Articles which met the inclusion criteria were read in full and data extracted by the team of three reviewers. Data extractions were second-checked by a different member of the team. Papers excluded and the reason for exclusion was recorded (available as Additional file 1: Appendix S4). The extraction form collected data on: first author/year; study design; sample size; population characteristics (type of group, condition/department, sex, age, other details reported); context; data collection method; outcome measures; type and details of the intervention; summary of results; main author conclusions; reported associations; and potential factors relating to applicability. The extraction form for systematic review included number of studies in the review, together with details of the inclusion criteria. Double counting was avoided by noting where included primary studies were also contained in included systematic reviews.

### Assessment of risk of bias

Quality assessment was based on the hierarchy of study design, together with use of a variety of checklists for each study type. For studies using comparative design we considered sources of potential bias based on the Cochrane criteria (selection bias, performance bias, attrition bias, detection bias, reporting bias) [14]. Where studies utilised before and after (pre-post) designs with no comparator group, or reported systematic reviews, we used the National Institutes of Health checklists [15]. In line with Cochrane recommendation we did not score elements, and instead provided a narrative rather than numerical indication of quality [14]. The completed checklists are available as Additional file 1.

### Data synthesis and analysis

Our protocol allowed for meta-analysis if heterogeneity permitted. However, the wide variety of models of integrated care, and multiple and complex elements contained therein, together with the heterogeneity of outcomes measured, contra-indicated the use of summary statistics. Instead, we report where there is greater or lesser strength (or certainty) in the evidence for each outcome reported [16].

It is important that any assessment of strength of evidence considers not only quality and volume of studies, but also considers consistency [17]. Our evaluation therefore draws on work by Hoogendoom [17], together with principles from the GRADE and CERQUAL rating schemes [16, 18], and our work from a previous systematic review with diverse evidence [19] to indicate a rating of strength (certainty) for each reported outcome across the included studies. Due to the nature of the intervention no studies were able to achieve the “gold standard” of double blinding and full randomisation and thus provide evidence considered to be “strong”. We therefore used comparator labels (stronger versus weaker), to provide a relative evaluation of strength. Appraisal of strength of evidence was undertaken by the research team at a series of meetings to establish consensus.

Each outcome reported in a study was recorded as either “increase”, “reduction” or “no significant difference (statistical significance).” We used these terms, as for some outcomes the judgement of being positive or negative depends on ones point of view. For example an increase in service usage may be positive for patients or the service, but may also be negative in terms of costs or detrimental effect on other services. Following rating of the outcomes in each individual study, we then applied an overall rating to the evidence across all studies which reported the same outcome. The rating scale was as follows: “stronger evidence” represented generally consistent findings in multiple studies with a comparator group design, or three or more systematic reviews; “weaker evidence” represented generally consistent findings in one study with a comparator group design and several non-comparator studies, or two systematic reviews, or multiple non-comparator studies; “very limited evidence” represented an outcome reported by a single study; and finally, “inconsistent evidence” represented an outcome where fewer than 75% of studies agreed on the direction of effect.

We separately rated evidence from the UK studies, evidence from systematic reviews, evidence from the international comparator studies, and evidence from international non-comparator studies, and then provided an overall rating of effect across the study types.

## Results

### Literature search results and study selection

**Fig. 1:**
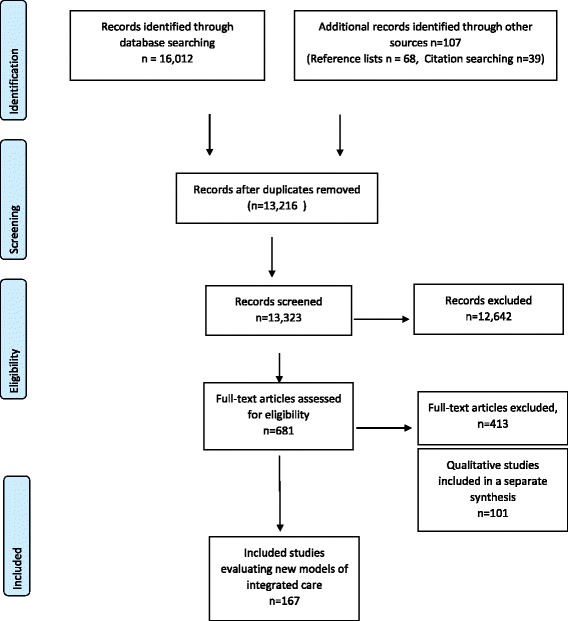
The process of study selection

Following screening of 13,323 unique citations, 167 documents representing 153 unique studies were eligible for inclusion. See Fig. 1 for a diagram illustrating the study selection process.

The list of studies excluded at the full paper selection stage and reasons for their exclusion is available as an Additional file 1: Appendix S4).

### Characteristics and quality of included studies

Of the 167 included documents, 54 reported studies carried out in the UK [20–73], and 43 reported systematic reviews [13, 74–115], we included 49 high quality studies from outside the UK using comparator group designs [116–164]. We included 21 low quality non-UK studies (no comparator group) [165–185] within a “light touch” analysis.

**Fig. 2:**
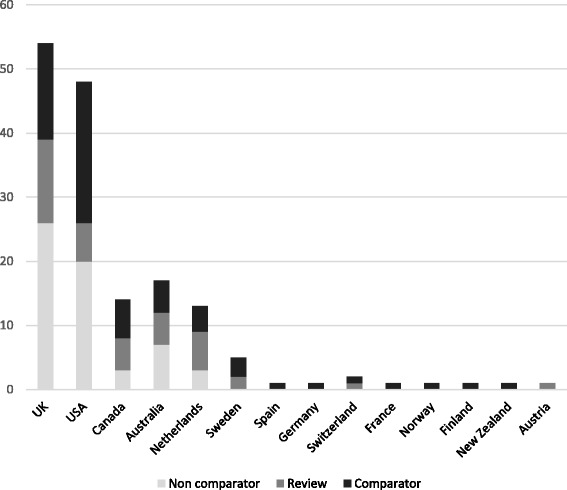
Country of origin and design of the included studies

We observed little overlap between primary studies and reviews, with time lags in publication of the systematic reviews meaning that the majority of their primary studies preceded our inclusion date of 2006. Figure 2 summarises the country of origin for the different types of study design.

While there were large numbers of studies from both primary/community services, and acute care, the larger group was initiatives implemented outside hospital settings. Thirty five studies were carried out in primary care/community contexts, 24 studies were carried out solely in hospital settings, and two were carried out in nursing homes. Nineteen studies specifically described both health and social care services being included in the integration, although reporting of specific details of partner organisations/services was often limited. Authors did not make links between the context and outcomes of initiatives, apart from reported issues regarding staff training and retention in social care [38]. and the benefit of physical co-location of services [32].

Of the included 54 UK articles, 16 reported findings from studies using higher quality comparator designs [25, 28, 30, 31, 34, 38, 40, 44, 49, 60, 63, 64, 67, 68, 71, 72]. Only two had utilised some form of random allocation to condition [44, 49], with allocation concealment not possible due to the nature of the intervention. Blinding of participants and personnel was also limited or not possible, with only four studies achieving this [30, 31, 49, 72]. Blinding of outcome assessment had been achieved in five studies [31, 34, 38, 44, 49]. The included UK studies fared better in regard to completion of outcome assessment, and reporting was assessed as being accurate for all but one [44] which had insufficiently discussed the study limitations. Overall therefore the UK studies were all considered to be at risk of potential bias, with none achieving all six criteria for reducing potential sources of bias.

The international comparative design studies rated slightly better in terms of randomisation with 19 (reported in 26 papers) having random allocation [116–119, 123–128, 131, 132, 136, 137, 139, 142, 144, 147–149, 152, 155, 156, 161, 163, 164], although only nine studies (reported in 14 papers) achieved allocation concealment [116, 118, 119, 123–125, 127, 128, 131, 132, 139, 161, 163, 164]. As with the UK studies, blinding was problematic as patients were unable to be blinded to their study arm. The incomplete reporting of outcomes data meant that in many cases it was not possible to judge the extent of attrition, although for three studies (reported in seven papers) large loss to follow up was reported [123–125, 136, 145, 146, 184]. Reporting was poor in around a third of the studies, making it difficult to judge the extent of possible selective reporting. Other limitations included small sample sizes leading to inadequate statistical power, with some concerns regarding the processes of allocation. As with the UK comparative design studies, none met all the criteria for reduction of potential bias.

The UK before and after/longitudinal studies demonstrated similar issues regarding blinding, with only one study clearly reporting that outcome assessors were blinded [66]. Generally participants recruited appeared to be representative of the population of interest, although often it was difficult to ascertain the recruitment process. Just over half the included studies reported sample sizes that were sufficiently large to have confidence in the findings. Only a third were judged to have clearly described the intervention and its delivery, and none reported taking measures at multiple time points prior to the intervention. Only just over half used statistical measures (such as *p* values) to evaluate change over time.

### Elements of models of integrated care

**Fig. 3:**
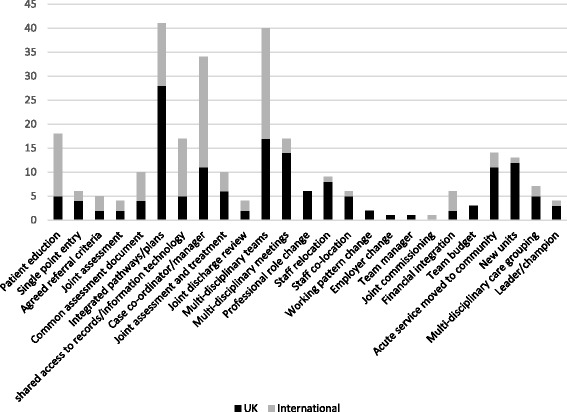
Elements of new models of integrated care in the included literature

The majority of the included models of integrated care were complex and multi-element interventions. The elements contained within them could be divided into four main categories: first, those with a focus on improving patient care directly; secondly, those that focused on making changes to organisations and systems; thirdly, those that focused on changing staff employment or working practices; and finally, those that addressed financial or governance aspects of integration. Many models incorporated multiple elements, and it was often challenging to elucidate the form and components due to limited reporting. The greatest number of elements we could identify in a single intervention was nine, which compared with other integrated care initiatives containing a single element. Typically models contained four to six elements. Case manager/case co-ordinator initiatives were more common in the international literature, whereas integrated care pathways/plans were more often a feature of models in the UK. Figure 3 summarises elements of new models of integrated care in the included studies.

### Effect on each outcome

We identified an extensive range of outcomes from the literature. We grouped these into three main areas: those relating to usage of health care resources; those relating to the quality of care received by patients; and outcomes for staff working experience. We adopted the four-item rating scale described in the Methods section to evaluate the quantity and consistency of available evidence for each outcome. We provide the rating for studies from the UK, international systematic reviews, international primary studies and finally an overall rating of available evidence. Where reports of outcomes were duplicated in multiple papers from the same study we identify only one instance, to avoid over-representation of these data. Additional file 2: Table S1 details the number of studies reporting each outcome, with each study (or papers from the same study) represented by either a plus “+” meaning that the study reported an increase for this outcome, or a plus/minus sign “±” meaning that the study reported no significant change for this outcome, or a minus sign “-” meaning that the study reported a reduction for this outcome. Symbols highlighted in grey are from UK studies using a higher quality comparative design.

The evidence was rated as stronger for three outcomes: that integrated care leads to an increase in patient satisfaction; that integrated care leads to increased perceived quality of care (staff perception in the UK studies, staff and patient perceptions in the non-UK studies); and that integrated care can lead to increased/improved patient access. UK studies indicated evidence of a reduction in waiting times and out-patient appointments, although the international literature as a whole was more inconclusive.

Nine of 11 UK studies evaluating differing types of interventions across a range of conditions and services reported increased levels of patient satisfaction [21, 23, 29, 32, 37, 44, 52, 61, 69]. All 11 systematic reviews reporting this outcome concluded that the evidence suggested a positive effect on patient satisfaction [13, 82, 85, 86, 92, 99, 102, 110, 111, 114, 184]. Four of six international comparator studies similarly reported increased satisfaction amongst older, acute and paediatric patient populations following service integration, case management and patient-centred medical home interventions [119, 136, 150, 159].

Four UK intervention studies reported staff perceptions of increased quality of care following service redesign, case management or integrated pathway interventions in hospital or primary care for older adults, general caseloads or patients with C-difficile infection [31, 50, 58, 69]. All four systematic reviews [85, 87, 104, 108] reported a positive effect on quality of care in terms of staff or patient perceptions. One of two international comparator studies (reported in three papers) supported the finding that quality of care was perceived by patients to have improved [123–125].

Five included (non-comparator) UK intervention studies reported that access to services in the community and/or specialists/intermediate care had increased [35, 41, 59, 72, 73]. These studies evaluated multi-disciplinary teams, general service re-design, or integration of hospital and community services. Two systematic reviews reported that access to services had “improved” [76, 104]. Three international comparator studies (reported in five papers) supported the finding that integrated care initiatives improved access [117, 123–126]. Two international non-comparator studies similarly reported improved access to services for patients [167, 179].

In regard to similarities and differences between studies carried out in the UK and in other countries, we found three areas of variance in rating between UK evidence and the evidence overall. Five UK studies offered evidence of a reduction in waiting times [27, 41, 49, 61, 71]. The international evidence however, is more inconclusive, with three studies indicating a reduction, two studies indicating no effect, and one an increase. UK studies found a reduction in out-patient appointments [31, 44, 53, 60, 67], however, the two international studies reporting this outcome found no significant effect. We found weaker UK-only evidence in three studies for the likelihood of care meeting patient preferences (predominantly end of life decisions) [20, 39, 65] with no included international studies evaluating this outcome.

Evidence regarding the following outcomes was rated as inconsistent: number of clinician contacts (five indicated a reduction, and three an increase); number of GP appointments (two UK studies reported a reduction and another UK study no difference); length of stay (24 studies reported a reduction, two studies found an increase, and 11 no effect); unscheduled admissions (10 studies found a reduction, two an increase; and nine no effect); number of admissions (24 studies found a reduction, five reported an increase, and nine no effect) although considered alone the systematic reviews provided stronger evidence of a reduction; re-admissions (nine studies, with eight from the same authors reported no effect, two studies found an increase and two a reduction); attendance at accident and emergency (nine studies found a reduction, two an increase and eight no effect); quality of care standards (two studies reported an increase and one no difference); and staff work experience (two reviews of UK studies indicated improved experience, and one international study indicated no difference).

The rating of very limited evidence (insufficient studies) was assigned to the following outcomes: prescribing rates; access to resources; time spent in accident and emergency department; the number of incidents/complaints; and identification of unmet need.

We also examined evidence relating to wider impacts across the whole of a healthcare system. The evidence was inconsistent regarding the impact on cost of provision (17 studies reported a reduction, two an increase and 20 no difference); community care activity (four studies reported a reduction, five an increase, and one no difference); secondary care activity (no studies reported an increase, four found a reduction, and two no difference); and overall healthcare utilisation (two systematic reviews found the evidence was unclear).

**Fig. 4:**
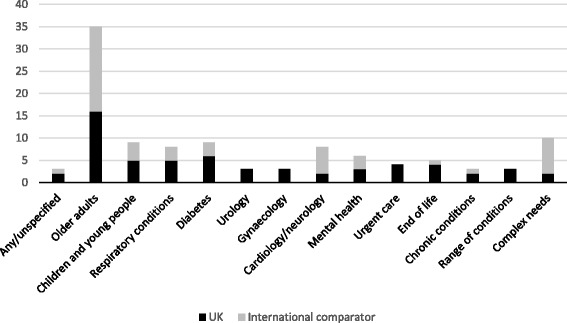
Included studies categorised by patient type/condition

We explored the potential for sub-group differences between different types of patients. Figure 4 summaries the types of patients and conditions in the studies included in the review.

We examined the data regarding outcomes and impacts for studies in the two largest sub-groups of patients - older adults, and populations described as having complex needs. We then compared this to the strength of evidence ratings assigned to the included studies as a whole. The effect of integrated care initiatives in older adult populations echoed the strength rating for all studies, with reports of increased access and patient satisfaction, and inconsistency in regard to admissions, emergency admissions, length of stay, patient contacts/service usage, and costs.

In contrast to the wider evidence base however, the evidence on patients described as having “complex needs”, suggested a stronger indication of positive outcomes in terms of reduced admissions and emergency department use, and weaker strength of evidence regarding reduced length of stay. The studies all utilised non-comparator designs however, so this indication needs to be treated with caution. We also looked for any patterns in regard to the type of initiatives that appeared to lead to more positive outcomes, with little clarity in signal beyond suggesting that integrated pathways as “stand alone” interventions may have a limited effect.

## Discussion

Models of integrated care encompass diverse initiatives that aim to improve integration of care across healthcare and between health and social care services. We identified diverse and frequently contradictory outcomes for models of integrated care reported in the included literature. Three outcomes appeared to offer stronger evidence of effect: firstly, that integrated care leads to increased patient satisfaction; secondly, that integration increases perceived quality of care; and thirdly, that integrated care increases patient access to services. UK-only evidence indicated that patient waiting time and outpatient appointments may be reduced, and patient wishes at end of life are met more often (although inconsistency or lack of evidence for these effects was found in the international literature). The system-wide impact on community and hospital-based services was unclear, with reports of both increased and decreased use of community services, although we identified no evidence to suggest that models of integrated care increase use of secondary care. Neither was there clear evidence regarding whether models of integrated care are cost neutral, increase or reduce costs. The review identified numerous changes to delivery of services which are subsumed within the label of models of integrated care. As highly complex interventions, these models challenge linkage of particular elements of initiatives to effects, with a lack of clarity on which key elements are causally associated with positive outcomes.

We highlight the challenges inherent when defining models of integrated care, given the lack of agreed definition and clear boundaries to the term. This limitation may have resulted relevant work being excluded from this review. We found it particularly challenging to distinguish between new models of care that are integrated/co-ordinated from those that are not during the screening and selection process. “Integration” could be used in a variety of ways, including to describe interventions which related to enhanced care or quality assurance but did not include staff working in new ways. Although our search terms enabled relevant citations to be retrieved, we recognise that indexing may be imperfect, and we may have not identified all studies of relevance. We also acknowledge a potential issue of publication bias, with studies reporting less positive outcomes potentially under-represented in the review. We highlight the paucity of literature reporting objective quality of care outcomes, with our findings regarding the effect on quality based on staff or patient perceptions.

One particular limitation relates to the lack of statistical summary of effectiveness (meta-analysis) although we would argue that not only did the heterogeneity of interventions and outcomes preclude this type of analysis, but also, in exploring the complexity of the area a strength of evidence approach was beneficial. Included studies highlighted the challenges in identifying causal relationships between models of integrated care, and service delivery impacts [76, 87, 120–122]. In view of this challenge, we used strength of evidence ratings to summarise where greater or lesser certainty existed in the literature, considering quality, volume and consistency of the evidence identified. Reporting strength by volume of studies (“vote counting”) may be imperfect, primarily indicating where there has been research activity. In exploring consistency as well as volume when assessing strength of evidence, we have sought to some extent to mitigate this limitation.

Evaluating outcomes and impacts from models of integrated care presents challenges in determining what a “good” outcome may be. In terms of financial outcomes, the effects of integrated care may be perceived differently by different stakeholders, offering contradictory incentives for achieving change. At an organisational level for example reduced activity in one sector may mean financial losses. There are also known to be considerable challenges in transferring money or resources between organisations in response to changed levels of activity. Another tension exists between cost-saving and provision of improved quality of care. Some studies reported that increasing quality of care for patients may come at increased cost for services already facing financial pressure.

The potentially positive outcome of increasing ease of access for patients, also offers contradictory effects. Improved access may be perceived positively by patients, and enable serious conditions to be identified and treated earlier; but also may incur a detrimental effect on costs and capacity. Recognition is growing that rather than new models of integration within services, reform at scale is required, with reconfiguration at a whole systems level including in the UK new forms of commissioning and contracting (the way that NHS organisations assess the needs of an area and then draw up contracts with suitable providers) [3]. The literature included in this review rarely focused on organisational change within integrated care models. This may reflect the challenges inherent in the organisational change process [186]. Some authors highlighted the continuance of varied pre-existing governance arrangements following integration of organisations, with progress on new models often reported to be particularly limited in the areas of budgets, financial, and contracting mechanisms [187].

The implementation of highly complex whole-system change interventions such as new forms of integration is known to be challenging [188], and differing degrees of success in implementation may contribute to the varying outcomes reported. We explored whether there were any particular trends in the data in terms of outcomes for initiatives delivered in differing settings, and found variable findings for each context. Similarly, examination of integration amongst health services versus combined health and social care did not indicate any particular trends in effectiveness. While there appeared to be no clear pattern of differential outcomes between settings or initiatives, there appeared to be potential for more positive outcomes amongst those categorised by authors as having “complex needs”, although currently most research evidence comes from studies in older adults. Further research is required to explore the potential for models of integrated care to impact on the care for other patient groups with complex needs.

## Conclusions

This review adds to the growing evidence that integrated care initiatives rarely lead to unequivocally positive effects, although the calls for integrated care have never been stronger. The potential for integrated services to increase patient contacts, is a particular concern in already over-stretched services. New models of care may be best targeted to particular patient groups (such as those with complex needs) rather than being seen as a panacea for all.

We identified surprisingly little evidence regarding the impact of integrated care models on patient experiences of services, beyond measures of reported patient satisfaction. There seems a need for further attention to how reconfiguration impacts on patients and carers, including whether service users perceive any change, or have greater knowledge of or involvement in services.

## Additional file

Additional file 1: **Appendix S1.** Study protocol. **Appendix S2.** Completed PRISMA checklist. **Appendix S3.** Search strategy. **Appendix S4.** Studies excluded at full paper screening. **Appendix S5.** Completed quality appraisals. (DOCX 197 kb)

Additional file 2: **Table S1.** Summary of studies and effect for each outcome (DOCX 335 kb)
