# Supplementary material for: The effects of integrated care: a systematic review of UK and international evidence
Source: BMC Health Serv Res. 2018 May 10;18:350. doi: 10.1186/s12913-018-3161-3 (PMC5946491; doi:10.1186/s12913-018-3161-3)
Supplement: Supplementary file 1 — Appendix S1. Study protocol. Appendix S2. Completed PRISMA checklist. Appendix S3. Search strategy. Appendix S4. Studies excluded at full paper screening. Appendix S5. Completed quality appraisals. (DOCX 197 kb) [file 12913_2018_3161_MOESM1_ESM.docx]

**Online supplementary material**

**Appendix S1**

**Study protocol**

**Project title:** Understanding new models of care in local contexts: a systematic review using frameworks to examine pathways of change, applicability, and generalisability of the international research evidence

**Summary of Research:** The NHS has been challenged to adopt new, flexible models of service delivery that are tailored to local populations [NHS England, 2014]. Evidence from the international literature is needed in order to support the development and implementation of these new models of care and to overcome reported potential issues and barriers [Ham & Murray, 2015].

The proposed study aims to support the development of new, flexible models of service delivery in the NHS by carrying out a systematic review of evidence relating to new models of healthcare. It will combine rigorous and systematic methods for identification of literature, together with innovative methods for synthesis and presentation of evidence. The proposed synthesis methods will address the identified need for: firstly, enhanced understanding of the mechanisms whereby new models of service delivery impact on healthcare and patient outcomes; and secondly, greater understanding of ways that models might work in different contexts.

The research team will carry out a systematic search of the international literature on new models of care. We will search electronic databases and use supplementary searching methods such as citation searching and reference list screening [Booth et al., 2013]. This systematic search will advance understanding by privileging **rigour** i.e. by focusing on formal evaluations and empirical designs. At the same time a UK-centric systematic search, with a more inclusive evidence threshold will identify contextually rich ephemeral materials (for example commentaries, editorials, news items, process evaluations, meeting reports) that will privilege **relevance** [Torejeson et al., 2015]. In this way both context and outcomes will receive corresponding attention within this bi-partite review for, as Pawson [2006] has stressed, “outcome patterns are contingent on ... assessments of both rigour and relevance”.

Evidence identified via these means will be synthesised in two ways. Firstly, the study will use data from included studies to develop an evidence-based logic model. Initially UK-based literature will be used to populate the model, which will set out evidence underpinning the pathway from new models of care to long term and system-wide impact. The logic model will detail: the types of service transformation which have been described; short term outcome measures reported; and longer term evaluation tools used to examine impact. The model will also outline the mechanisms of action (i.e. theory of change) for the pathway from these new models of care to different types of impact, together with factors reported as moderating or mediating elements. Following development of the prototype model we will then scrutinise the international literature, making comparisons and contrasts between this evidence and the UK evidence. The logic model will thereby act as a translation tool between multiple and diverse evidence types, and enable a contextually sensitive analysis.

The second method of synthesis in the proposed work will further examine comparisons and contrasts between the UK and international literature in order to develop a framework of factors potentially impacting on generalisability and applicability of the literature. This element of the work will address the need for understanding how particular models might work in different local contexts.

The final phase of the study will use consultation with key stakeholders to examine the clarity and resonance of the developed logic model, and also to gain feedback regarding the assessment of generalisability and applicability. This builds on evidence synthesis methods we previously developed for a systematic review of demand management interventions commissioned by the HS&DR programme and subsequently have used widely and effectively for sharing the findings, in a similarly complex topic area, with practitioners and service commissioners [Baxter et al. 2014; Blank et al. 2014].

**Background and rationale:** It has been argued that the growing financial and service pressures in the NHS cannot be tackled without transforming how health and care are delivered. The NHS Five Year Forward View Plan [NHS England 2014; 2015] sets out a view on how services need to change and what models of care will be required in the future, with a common thread being the need to break down barriers between services through greater integration of care [Shortell et al., 2015]. It is proposed that there should be new networks of provider organisations (organised horizontally as multispecialty community providers or vertically as primary and acute care systems) which form the bedrock of provision [Long et al., 2015]. Thirty-seven “vanguard sites” have been identified to develop and test new models of care, including enhanced health in care homes and new approaches to urgent and emergency care as well as multispecialty community providers and primary and acute care systems. The rationale underpinning the development of the vanguard sites programme is to evaluate a small number of different models, while allowing flexibility in the way models are implemented to meet local needs.

A study by the Kings Fund [Ham & Murray, 2015] highlighted that change was needed at a whole-system and governance level, with the development and rapid implementation of integrated models of care needing to draw on resources and expertise from across the local health system. This primarily qualitative work analysed five case study sites that have developed more integrated models of care. The work found that significant barriers to implementing changed systems included: a lack of clarification of roles and responsibilities; competition for funding; and challenges in engaging primary care. The authors of this work also highlighted that “one size does not fit all” with different models developed at each of the case study sites. Common themes regarding the requisite types of changes encompassed: delivering more care beyond the hospital walls; changing the future size and shape of acute hospitals; an increased role in prevention and population health; and new organisational models with local partners.

Other studies have similarly reported that cultural and structural barriers have proved difficult to overcome in the drive to provide integrated care [Ahmed et al., 2015]. A key challenge is to create an environment of collaboration between all providers, including primary and secondary care [Long et al.]. Authors have highlighted that there is a need for greater understanding regarding how integrated care can be best delivered, and a requirement for further clarity regarding how new models of integration may impact on patient outcomes [Robertson, 2011].

It has been suggested that models from the United States such as Accountable Care Organizations may be useful frameworks from which the NHS can learn. However, studies have also highlighted that it is important to consider the difference in contexts before implementing the same models in different areas [Ahmed et al. 2015]. The NHS vanguard sites have been encouraged to develop new models of care by adapting systems to local needs and configurations [NHS England, 2015]. However, while individual models should be rooted in local communities and have a “local resonance”, it has also been emphasised that it is important to identify simple standard approaches and products which can be replicated across the country [NHS England, 2015].

The proposed study will add to the existing body of knowledge by providing a critical summary of the international literature on new models of care. The review of the literature will provide knowledge regarding key ingredients of care models and will identify best practice and areas of learning which may be important in contributing to the success of a programme. It will also detail potential outcomes and impacts reported in the literature which will inform the planned multi-faceted approach to measurement and evaluation of new care models in the NHS. The logic model method that will be used to synthesise the review findings is intended to add to the body of knowledge by illuminating complex pathways between models of care and long term health impacts. The method will provide an accessible summary of the literature, and will inform the draft logic models which every vanguard site has been asked to develop as the basis for further development and refinement of local models. The analysis of factors relating to generalisability and applicability in the proposed study, will contribute to knowledge by providing key information to commissioners, service managers and practitioners regarding implementation of care models in their local context, and support understanding of how care models might be replicated in other local care systems.

**Evidence explaining why this research is needed now:** In order to support the development and introduction of new care models in the NHS via vanguard sites, a rigorous and systematic review of the international evidence is needed. A range of models which are rooted in local communities have been developed, and there is now a need to understand the key ingredients of successful new models of care described in the international literature. This is needed in order to inform evaluation and further replication within the NHS.

The proposed systematic review is particularly relevant in its emphasis on using the international evidence to understand the complex pathways from new models of care to longer term impacts, and also to explore the mechanisms of action underpinning change pathways. It also has as its focus the understanding of components of interventions that influence generalisability and applicability. This focus will directly inform understanding of how models might work in different contexts. The vanguard sites have been asked to develop draft logic models, and the logic model output from the proposed work will be valuable in helping to identify where local models have common characteristics to each other, and to the international literature.

**Aims and objectives:** The proposed study aims to carry out a rigorous and inclusive systematic review of evidence underpinning new models of healthcare. It will have the following aims:

1. To examine what can be learned from the international literature regarding key elements of implementation, and potential impacts of new models of care.

2. To examine reported mechanisms of change and outcomes and impacts associated with new models of care.

3. To assess how generalisable the findings from the international literature might be to different local populations and contexts, and examine how mechanisms of change might operate differently in differing local services.

4. To explore how the international evidence can be applied in a UK context, and what factors of applicability for the new models of care may be important for local populations and local service contexts.

The work will have the following specific objectives:

1. To carry out a systematic review of the international literature on new models of healthcare.

2. To use a logic model method to outline mechanisms of change underpinning the introduction and outcomes from new models of healthcare delivery, including potential barriers and facilitators.

3. To explore how the developed model resonates with the views and experiences of key stakeholders.

4. To develop a framework which details factors that may impact on the generalisability or applicability of the research literature, and to use this framework to evaluate models of care reported.

**Research Plan/Methods:** The proposed study will combine established systematic reviewing methods for identification of literature, with innovative methods of analysis and synthesis to examine mechanisms of change, generalisability, and applicability of international evidence to local contexts. The outcomes from this work will be two syntheses: firstly, a logic model synthesis outlining and comparing the UK and international evidence on new models of care; and secondly, an applicability synthesis that will develop and use a methodology of wider value within health service and delivery research.

**i) Identification of literature**

*Search strategy:* A systematic search of key health, medical and social care databases will be undertaken to identify relevant studies published since the year 2006. We will search from this year as a previous review [Davies et al. 2006; 2008] is available which included studies published up to 2006. Search terms will be developed from keywords with input from the information specialist on the team via an iterative process of scrutinising retrieved papers to inform further searching, and MeSH terms. The initial consultation sessions will also be used to inform keyword identification. Electronic databases searched will include MEDLINE, EMBASE, the Cochrane Library (including the Cochrane Database of Systematic Reviews, Cochrane Central Register of Controlled Trials, Database of Abstracts of Reviews of Effects), PscyINFO, SCI and SSCI, and CINAHL. We will review the titles and abstracts of all articles in a small number of key journals: International Journal of Integrated Care, Journal of Integrated Care, International Journal of Care Coordination. Additionally, Google Scholar will be searched for relevant articles. The search process will be recorded in detail with lists of databases searched, date search run, limits applied, number of hits and duplication as per PRISMA guidelines.

In addition to standard electronic database searching other iterative searching techniques will be employed including citation searches of included articles and authors, searches on any key models of care we identify, additional targeted searching on keywords and concepts identified from the included papers, hand searching of reference lists, and contacting key authors and experts to obtain further relevant published and unpublished material. Relevant review articles will also be used to identify studies. In particular we will examine all articles citing the two published versions of the Powell Davies review [2006; 2008] which provide 78 and 29 citations respectively. In this way a community of citing authors will help us in the definition of “new models of care”.

*Initial scoping work:* An initial scoping search has been carried out by the information specialist on the team (AS) to gauge the volume of literature and to provisionally test and refine the proposed search strategy. Ovid Medline was searched using the search terms listed below resulting in 7,256 hits. We have carried out some initial sifting and categorisation of these citations (see diagram below).

We have also carried out searching in further databases (Cochrane, PsychINFO, and CINAHL) to estimate the total volume of literature. Across all these databases we have retrieved 20,103 references.

**7,256**

Retrieved citations

**6,945**

Do not meet inclusion criteria

**34** Reviews or relevant discussion papers

**242** (of which 80 UK) Potentially relevant intervention studies

**35** (of which 13 UK)

Potentially relevant non-intervention studies

**Scoping search**

A combination of MeSH subject headings and free-text terms were utilised.

**MeSH:** Health Care Reform, Organizational Innovation, Quality Improvement, Health Priorities, Accountable Care Organizations, Delivery of Health Care, Integrated

The above MeSH terms were combined with the Floating Subheadings Organization & Administration [og] OR Trends [td]

**Free-text terms** included (searching in title OR abstract): care model(s) and “new”, service delivery model(s), healthcare model(s), transformation of service or care, integrated care, integrated health system(s), vanguard(s), accountable care, future-proofing, service redesign, “five year forward plan”

*The search was limited to humans, English language and the publication dates 2006-present.*

*Additional search for UK evidence:* In the proposed study we will carry out a supplementary search process to locate evidence relevant to the development, implementation and evaluation of new models of care in the NHS in England, focusing specifically on the models being implemented by the NHS vanguard sites (or a random/purposive sample). This will involve contact with NHS England and/or vanguard site representatives to obtain as much information as possible about the new models of care being implemented. Starting from information obtained from these sources and/or from published literature, we will apply CLUSTER methodology (Booth et al. 2013), modified if necessary, to understand as fully as possible the context and underlying concept(s) of the intervention(s) under study. For this part of the search process, we will consider evidence for inclusion based on relevance to the process of developing and implementing a new care model, (although discursive papers with little or no data will be excluded).

**ii) Inclusion/exclusion criteria:** The following inclusion/exclusion criteria will be applied to assess the relevance of studies identified during the searching process.

*Target population:* Patients receiving a healthcare service and staff delivering services.

*Target interventions:* We will define new models of care as changes to service delivery which increase integration and coordination within primary health care (PHC) and/and between PHC, health and health related services (including social care). We will include: Polyclinics; Accountable Care Organisations; and Integrated Care Pilots. Based on papers identified during the scoping search, a potential typology outlining the types of intervention that will be included is:

1. Interventions with a focus on service re-design/re-configuration. This may be sub-divided into - single point of access, joint clinics or sessions, integrated care pathways, and re-location of services.

2. Interventions with a focus on workforce changes. This may be sub-divided into changed roles, and further education/training.

3. Interventions with a focus on integration of different services, or working across service boundaries.

4. Greater co-operation between services (for example using common assessments), but without the aim of integration.

5. Interventions with a focus on financial strategies.

6. Information systems or other technology to promote new ways of working.

*Control/comparators:* The review will examine interventions with comparator groups (such as care networks) and also those with no comparator.

*Outcome measures:* As one of the objectives of the work is to identify and report measures of outcome and impact that have been used in the literature, we prefer not to specify a priori the outcomes measures that will be included. The review will include studies with any outcome relating to the delivery of services (effectiveness or efficiency) or an impact on patient care, or staff. This will include quantitative measures, together with views/perceptions of patients/service users, staff and other key stakeholders.

*Study design:* We will carry out a rigorous search of the international literature for all relevant systematic reviews, randomised and non-randomised controlled trials, prospective and retrospective cohort studies with and without comparators, and other before and after/longitudinal studies. With the increasing recognition that a broad range of evidence is needed to inform the depth and applicability of review findings, the review will be inclusive and encompass both experimental and observational studies, together with qualitative work reporting views of service users or staff delivering services. Descriptive or discursive papers will be excluded. A particular focus of the synthesis will be the inclusion of UK formal service evaluations of diverse study types and methodologies.

*Other inclusion/exclusion criteria:* We will include studies from any country which is a member of the Organisation for Economic Collaboration and Development (OECD). Studies in these developed countries will be of most relevance to UK health systems. We will examine studies published in English, however we will consider translation of any key international papers which have abstracts in English. In order to examine the most relevant studies, the review will include work published since 2006.

**iii) Selection of papers:** Retrieved citations will be uploaded to EndNote, and title and abstracts (where available) of papers will be independently screened by two reviewers and any queries regarding inclusion will be discussed by the full team. Full paper copies of potentially relevant articles will be retrieved for systematic screening. The screening process will identify papers which are of relevance to answering questions of intervention outcomes, or provide data regarding contextual factors, or perceived barriers and facilitators to successful outcomes.

**iv) Data extraction:** Studies which meet the inclusion criteria will be read in full and a brief data extraction will be completed. A data extraction form will be developed using the previous expertise of the review team, trialled using a small number of papers, and refined as necessary. Extracted data will include: study population, comparator, baseline characteristics of the population and service provision: details of the model of care: and study findings. In addition to these data we will be identifying elements relating to implementation of care models which may impact on generalisability, or relate to applicability. Data will be extracted by one reviewer and checked by a second.

**v) Methods for combining/synthesising findings and different forms of evidence:** The literature on new models of care presents two key challenges for systematic review methods. Firstly, it is increasingly recognised that any intervention in healthcare can be considered to be complex, with individual and organisational factors affecting how and if interventions lead to improved outcomes [Rees et al., 2004]. Interventions such as new care models, which act at a system or organisational level, or even across organisational boundaries, provide considerable additional complexity due to their multi-factorial processes. The new care models programme has been described as being “complex in its breadth and depth” [NHS England, 2015]. This complexity presents difficulties for systematic review methodologies which seek to quantify or report clear intervention-outcome effects.

A second challenge to review and synthesis of this literature comes from the extensive variety of models which have been introduced. This diversity in type is particularly apparent as many models have been developed in response to local needs and configurations. New models of care may adopt a number of different approaches and organising principles which respond to the local context. This diversity presents challenges for considering how generalisable the findings from a study may be, and how applicable evidence from varied national and international research may be to a particular local context.

We propose to use two main methods of synthesis to overcome these key challenges, and enable the integration of different forms of evidence. The first method, will be to synthesise the identified UK and international quantitative and qualitative literature using logic modelling techniques, to develop an evidence-based framework of links between new models of care and health impacts. This method, which was developed by the team [Baxter et al. 2010], has been used successfully in previous systematic review studies we have carried out [for example Blank et al. 2014; Allmark et al. 2012], and is ideally suited to the analysis of complex, system-based interventions.

In the logic model method, data from included studies are extracted and analysed to produce an intervention typology, detail regarding the range of outcomes reported, factors which may be influential in the pathway from a new model to health impacts, and reported associations between elements of the model. Both quantitative and qualitative data are used to underpin construction of the elements of the model. The model will outline evidence in the literature regarding different models of care, relationships between contextual factors, inputs, processes and outcomes [Joly et al. 2007; Anderson et al. 2013]. Lines between each element in the model will demonstrate the logic or theory of the interventions and portray “if......then” relationships in the causal chain from new models of care to system wide health impacts.

Initially, the work will use UK published literature, grey literature and other forms of documentary evidence relating to new models of care to develop an initial or “start” logic model. This will include any available data/documents relating to the Vanguard sites. Following development of the UK-based logic model the second phase of the work will entail examination and synthesis of the international (non UK) literature, making comparisons and contrasts between the primarily internally valid and externally valid domains. We will initially search for and include systematic review level international studies alongside the UK evidence, to underpin a further draft of the model. We will use a “saturation and gap filling” approach to further develop the model via subsequent search iterations, using international primary evidence where we are unable to find reviews to explore gaps in the evidence base. The logic model framework will act as a translation tool between findings from review of the UK evidence and the international literature, and enable contextually sensitive synthesis of multiple and diverse evidence types.

In a further phase of work we will use a second method of synthesis to compare and contrast the UK and international evidence, in order to develop a framework of factors of applicability and generalisability. This additional method will be employed to address the diversity in type of new models of care, and challenges in evaluating generalisability and applicability of the literature to varied local contexts. We will move between the UK and international literature to examine elements of setting, intervention and outcomes. From this analysis we will develop a framework of generalisability and applicability to local NHS contexts in the form of a checklist, which will be used to evaluate the evidence that we find. The framework will be applied to each reported model to provide an evaluation of generalisability and applicability. In addition to detailed analysis, indications of greater/lesser applicability to the UK NHS will be derived from this framework and indicated on the logic model.

**vi) Assessment of quality and relevance of studies:** The critical appraisal of included evidence is considered a key part of the review process; although remains the subject of debate in the field, with no single recognised tool. There is also variation in views regarding the use of scoring systems and methods for appraising strength of evidence across studies (particularly in qualitative work). Pawson et al. [2003] argued that there is no hierarchy of knowledge, although users of knowledge need to understand that some types of knowledge are more relevant to some purposes than others, and be aware of the quality and reliability of the knowledge. The appraisal of quality and evidence in the proposed study needs to take account of the wide range of study types that we are likely to find, and recognise that a higher number of papers in an area does not necessarily indicate greater strength of evidence; only where more work has been carried out. This is particularly important given that the rigorous evidence base is likely to be dominated by studies from outside the UK, whereas the most relevant studies will be derived from the UK.

Our approach to assessment of strength of evidence will be based not only on the quality and volume of studies, but also consider consistency of the evidence [Hoogendoorn et al., 1999]. We have used this approach successfully in a previous review and logic model study with diverse evidence [Baxter et al., 2014]. We will report where, in the logic model there is greater or lesser strength of evidence for associations and outcomes.

Stronger evidence (Level i) will be defined as: generally consistent findings in multiple higher quality studies.

Weaker evidence (Level ii) will be defined as: generally consistent findings in one higher quality study and lower quality studies, or in multiple lower quality studies.

Very limited evidence (iii) will be defined as: only one study available

Inconsistent evidence (iv) will be defined as: inconsistent findings in multiple studies. Study findings will be considered to be inconsistent if fewer than 75% of studies reported the same conclusions.

Consideration of individual study quality will be based on the hierarchy of design, together with consideration of potential for bias as recommended by the Cochrane Collaboration [2011] (selection bias, performance bias, attrition bias, detection bias, reporting bias). Consideration of systematic review and qualitative evidence will be based on the Critical Skills Appraisal Programme [NHSPRU, 2006] checklists. We will also use emerging methods relating to Confidence in Review Findings (CERQual) that examine relevance, adequacy, and coherence (the qualitative counterpart to consistency) alongside methodological quality of qualitative studies.

Any concerns regarding study quality will be identified and recorded during data extraction. Appraisal of overall strength of evidence will be undertaken by the research team at a series of meetings to establish consensus.

**vii) Consultation phase:** The final phase of the work will be a period of consultation. This consultation will be carried out via individual and group sessions with stakeholders at local and national levels including: Commissioning Managers, Portfolio Leads, Portfolio Managers, Clinical Executive Directors, members of the Vanguard Evaluation team, together with Service Managers and clinicians from Vanguard sites. This phase of the work will be important in terms of validating the developed model. The consultation will be used to seek feedback regarding the hypothesised causal chains and any areas for amendment, and to explore practitioners’ and patients’ understanding of the model in order to ascertain the usefulness of the framework as a communication tool. We will also seek feedback regarding the assessment of generalisability and applicability and seek input regarding any further dissemination strategies and/or impact activities.

**Design and theoretical/conceptual framework:** Logic model methods provide a graphical description of a system and are designed to identify important elements and relationships within that system [Anderson et al. 2013]. They are one form of theory-based evaluation that focuses on relating hypothesised links between an intervention and its constituent parts to its outcomes and long term impacts. Logic models are concerned with examining the processes of implementation, mechanisms of change and participant responses in order to develop hypothesised links or a “theory of change” [Weiss, 2007]. They serve as a tool to represent the causal system of interest, set out proposed causal pathways in the relationship between the intervention and its health and other outcomes, and identify potential moderators of that relationship [Anderson et al. 2013]. Outcomes are conceptualised as being the end of a chain of intermediate changes which the evaluation process seeks to track, with each intermediate point predicting the outcomes that may occur in the future [Dyson & Todd, 2010]. Theory-based approaches focus on assessing the validity of the theory on which an intervention is built and are concerned with opening up the black box of interventions and outcomes to uncover underlying mechanisms [Foss Hansen, 2005]. It is argued that without having a clear understanding of the assumptions underlying an intervention and how it is supposed to work, evaluators cannot ascertain whether it did work and why it did or did not achieve the intended benefits [Rossi et al. 2004].

Logic models, and other theory-based methods of evaluation such as realist synthesis, are becoming increasingly recognised as important additions to conventional methods of evaluating efficacy and effectiveness in systematic reviews. It has been argued that standard approaches can lead to disappointingly inconclusive findings regarding the success or failure of interventions due to their lack of examination of contextual factors [Pawson and Tilley, 1997]. Also, it has been highlighted that factors of process and the fidelity of an intervention may be overlooked in conventional methods of synthesis [Nilsen, 2007]. Theory-based evaluation approaches in contrast view understanding the context of an intervention as vital in attributing causation, and for gaining an understanding of mechanisms and impact. It has been emphasised that contextual factors need to be fully examined if an evaluation is to address issues of external validity [Blamey & Mackenzie, 2007].

The key strength of logic models is in linking complex system processes to system outcomes, and thus guiding the development of strategies and research tools for making system improvements [Handler et al. 2001]. Logic models have been recommended for evaluating highly complex, multi-site interventions with multiple and/or indeterminate outcomes [Connell & Kubishch, 1998] and provide a conceptual basis for explicit reporting of the methods and assumptions used within the synthesis [Anderson et al. 2013]. Health impacts following service re-organisation may be long-term, with evaluation complicated by multiple influential factors. The identification of intermediate impact, indications toward longer term outcomes and understanding processes between them is therefore of key importance. A logic model approach to synthesis is well-suited to analysing and reporting this literature, and examining the mechanisms and impacts underpinning new models of service delivery. A recent multi-author supplement on the systematic review of complex interventions counsels: “Independent of the overall synthesis strategy adopted, integrating diverse types of evidence collected from a wide set of study designs requires a coherent logic or conceptual model that can inform the design (structure and parameters) of narrative and statistical approaches to evidence synthesis” [Anderson et al. 2013]. This approach to synthesis of the international literature will also resonate with the draft logic models that every vanguard site has been asked to develop.

The terms “generalisability” and “applicability” are often considered to be synonymous. However, in the proposed study we are using the term “generalisability” to describe how relevant the results of a study might be to other sites and populations. We use the term “applicability” to refer to information regarding the study processes, and insights into whether and how an intervention may work in other situations. In the proposed study we will draw on work by Burchett et al. [2011] who reviewed published frameworks that included criteria for the assessment of external validity, applicability and transferability. The frameworks identified in this work considered elements relating to setting, intervention, outcomes and evidence. Examples of specific elements include: can the intervention be delivered elsewhere (is it feasible, can adequate coverage of the population be achieved, is the intervention acceptable to recipients); does the intervention meet recipients' needs (do potential recipients have similar needs to those of the original study participants); are investigators explicit about pathways/mechanisms/hypotheses through which an intervention is expected to act and about how intervention processes are influenced by context. The understanding of how evidence (in particular international evidence) can be applied to varying local situation and contexts is a key challenge for systematic review synthesis. The method for evaluating applicability and generalisability that we propose in this work, will assist decision-makers in evaluating how relevant research findings may be to their local organisations and services, and will be of value to future reviews of health services and delivery.

**Sampling:** For the consultation phase we will use our links with local and national commissioners, service providers and practitioners to gain input from a representative range of stakeholders. We will purposively sample in order to gain diversity in participant role and background. Previous work by the team [Baxter et al., 2014] has indicated that input from around 30-40 individuals should enable a point of saturation in views/perceptions to be reached.

**Setting/context:** Health care service delivery including primary and secondary care.

**Data collection:** Not applicable

**Data analysis:** See methods for analysis/synthesis section.

**Dissemination and projected outputs:** The main output will be a report providing a critical synthesis of evidence underpinning new models of care. Evidence based practice requires policy makers and practitioners to have readily available access to information on interventions that have shown to work or not work, or have the potential to cause harm. Systematic reviews are an established way of exploring the effectiveness of interventions and a cornerstone of evidence-based practice in order to identify, evaluate and summarise the findings of all available research evidence. The findings of the synthesis will also be presented as an evidence-based framework identifying key aspects of applicability and generalisability which will inform decision-making regarding the transferability of new models to local care contexts. The framework will provide an overview of evidence in an accessible form for stakeholders, and will further the understanding of how elements at a local level and wider organisational structure may enhance or provide obstacles to the implementation and outcomes of new models of care.

The work is expected to have an impact at a number of levels - researcher, practitioner, service managers and commissioners.

Researchers - the synthesis will provide an overview of the current state of knowledge in the field, indicate where further research is needed and the framework is expected to be a useful tool for understanding evidence in other areas of healthcare research. Whatever the findings, the work will highlight the importance of considering the applicability and generalisability of systematic review findings when considering evidence within the academic community. It will be complementary to and enhance other work on review methodology that is ongoing, such as methods of including both quantitative and qualitative synthesis, and incorporation of wider sources of evidence. We envisage that this framework will become a knowledge translation tool for use in future outputs of the NIHR HS&DR Programme where rigour is dominated by non-UK studies, but where relevance is located in lower study designs. Systematic reviews are used by researchers across the health disciplines to summarise current levels of evidence, and the proposed work will add to the methodologies available for ensuring impact on practice and service delivery.

Practitioners and service managers - the work will inform the optimal introduction of new models of care and highlight factors which may be mitigating the effectiveness of any models currently in use. The framework may provide a valuable resource for undertaking local evaluation of implementation and effectiveness and further the understanding of practice and delivery of new models of care. Research currently suggests that practitioners make limited use of available research evidence and find challenges in accessing and understanding systematic review findings due to difficulties in understanding how the review findings may be applied to their particular local context. The work to be carried out in this proposal aims to directly address this challenge and provide practitioners and managers with a framework for understanding the review evidence.

Commissioners - the work will outline evidence underpinning new models of care in use or under consideration, and provide insights into how factors within their local context may influence the implementation and outcomes of particular models.

**Impact activities:** The findings will be disseminated (in addition to the HS&DR journal) via conferences, as well as high impact peer reviewed journals. The team have a strong track record of publishing previous work in quality journals and of published papers attracting high interest. The University Media Centre will provide support for disseminating research findings via the media, both locally and internationally. Data from the research will be available via a report accessible from the University website. The review will be registered in the PROSPERO database, and in conjunction with the White Rose repository we are able to make journal articles widely available subject to publisher restrictions.

As a University department with a strong teaching and postgraduate research element, the ongoing research and findings will be disseminated via our established teaching for Medical students and other healthcare Masters level courses. Members of the team have current and will have future UK and international students undertaking dissertations and PhD study in the department, which will provide further opportunities to disseminate the findings and build potential future research studies in the area. The team have established links with a wide range of practitioner and commissioning groups from previous and other ongoing research which will be used to provide opportunities for influencing future practice. The work will also be disseminated via training sessions for professionals and researchers. The team and the location where the study will be based have established expertise in providing short courses on secondary research methods to a variety of audiences including medical specialist registrars, other health professionals, PhD students and the research community. Findings from this work will be incorporated into these courses and influence the training of future health researchers.

**Plan of investigation and timetable**

| Month | 1 | 2 | 3 | 4 | 5 | 6 | 7 | 8 | 9 | 10 | 11 | 12 |
| --- | --- | --- | --- | --- | --- | --- | --- | --- | --- | --- | --- | --- |
| Protocol development |  |  |  |  |  |  |  |  |  |  |  |  |
| Searching |  |  |  |  |  |  |  |  |  |  |  |  |
| Data extraction |  |  |  |  |  |  |  |  |  |  |  |  |
| Synthesis and development of logic model |  |  |  |  |  |  |  |  |  |  |  |  |
| Development of applicability and generalisability framework |  |  |  |  |  |  |  |  |  |  |  |  |
| Consultation phase |  |  |  |  |  |  |  |  |  |  |  |  |
| Report writing and dissemination |  |  |  |  |  |  |  |  |  |  |  |  |

**14. Project management:** The lead applicant will act as project manager and will oversee timely completion of the project milestones. Team meetings will be scheduled on a fortnightly basis as this has been a proven method of ensuring good communication between team members and co-ordination of work in previous projects. The project team is based within the same University department and in close working locations which will enhance joint working. The previous and current experience of the team in working together has established successful mechanisms of collaboration which will contribute to efficiency of project completion within the rapid timescale. We will establish a PPI group to maximise lay involvement in the study.

**Approval by ethics committee:** We intend to consult with the University of Sheffield School of Health and Related Research Ethics Committee to gain confirmation that the consultation phases of the work will not require ethical approval. Previous studies with similar consultative components have not required approval as we are not collecting and analysing data beyond receiving verbal and written comments.

**Patient and public involvement:** We have consulted with a local PPI group which includes lay members with experience of a variety of health and social care services and models of care (the Sheffield Palliative Care Studies Group) to obtain their input regarding writing the lay summary for this grant proposal. In particular, we wanted feedback on how to make the topic area ("new models of care") clear and meaningful to patients and the public. At a meeting with the group some members suggested ways of describing the topic area that might make it easier for lay readers of the proposal to understand. This increased our understanding of what the topic might mean to patients and the public and the importance of using terms and language that is familiar to them. Members of the group were able to ask questions about our proposed methods which allowed us to reflect on the clarity of our study design. Thus, we have used feedback from the group to write the lay summary and to facilitate clarity of the proposal.

During our meeting we asked whether the group would be interested in being involved in the study should we be successful in being awarded funding and also the ways that lay representatives may be involved. The group considered that a key challenge of the topic area for this work was making it clear and meaningful to a lay audience. When we asked what the term “new models of care” meant to them, only one member had heard of the term, few participants were able to make any guesses what it might mean. The group indicated that they would be interested in helping us to produce a piece of research that was meaningful to a lay audience. This would involve PPI input at the design stage as well as continuous input throughout the duration of the project as part of a lay advisory team.

We intend to form a project-specific public involvement advisory group for the duration of the work. One or two individuals from each of the 15 PPI groups co-ordinated by a local NHS Trust have been invited to join the public involvement advisory group (including Cardiology and Cardiothoracic Surgery Research Patient Panel, Community Infections Patient Panel, Bone Research Lay Advisory Panel, Obstetrics, Gynaecology and Neonatology Research, Sheffield Addiction Recovery Research Panel, Sheffield Emergency Care Forum) together with a generic online panel. We intend that this advisory group will have representation across different NHS service users. It is anticipated that this group will meet three times during the project: early phase, middle phase and towards the final phase. The public involvement advisory group will provide input on search terms and existing models and have a particularly important role to play in assessing whether the logic model produced from the work is understandable to a wide audience. PPI will also be important at the stage of writing the report to maximise meaningful messages for lay readers. We will obtain suggestions from the PPI group for disseminating the findings of the study in ways that reach the public, particularly since this topic has the potential to impact on the broad population.

**References**

Ahmed F, Mays N, Ahmed N, Bisognano M, Gottlieb G. Can the Accountable Care Organization model facilitate integrated care in England? Journal of Health Services Research & Policy 2015: doi:10.1177/1355819615590845.

Allmark P, Baxter S, Goyder E, Guillaume L. Assessing the health benefits of advice services: using systematic review and logic model methods to explore complex pathways. Health and Social Care in the Community 2012; DOI: 10.1111/j.1365-2524.2012.01087.

Anderson, L. M., Oliver, S. R., Michie, S., Rehfuess, E., Noyes, J., & Shemilt, I. Investigating complexity in systematic reviews of interventions by using a spectrum of methods. Journal of clinical epidemiology 2013; 66: 1223-1229.

Baxter S, Killoran A, Kelly M, Goyder E. Synthesizing diverse evidence: the use of primary qualitative data analysis methods and logic models in public health reviews. Public Health 2010; 124: 99-106.

Baxter S, Blank L, Woods H B, Payne N, Rimmer M, Goyder E. Using logic model methods in systematic review synthesis: describing complex pathways in referral management interventions. BMC Medical Research Methodology 2014; 14: 62. doi:10.1186/1471-2288-14-62.

Bienkowska-Gibbs T, King S, Saunders C, Henham M. New organisational models of primary care to meet the future needs of the NHS: a brief overview of recent reports. London: RAND Europe, 2015.

**Blamey** A, **Mackenzie** M. (2007) Theories of change and **realistic evaluation**: peas in a pod or apples and oranges? Evaluation 2007; 13: 439-455.

Blank L, Baxter S, Woods HB, Goyder E, Lee A, Payne N & Rimmer M. [Referral interventions from primary to specialist care: A systematic review of international evidence](http://dx.doi.org/10.3399/bjgp14X682837). British Journal of General Practice 2014, 64(629), e765-e774.

Bonell C, Oakley A, Hargreaves J, Strange V, Rees R. Assessment of generalisability in trials of health interventions: suggested framework and systematic review. BMJ. 2006 August 12; 333: 346–349.

Booth, A., Harris, J., Croot, E., Springett, J., Campbell, F., & Wilkins, E. (2013). Towards a methodology for cluster searching to provide conceptual and contextual “richness” for systematic reviews of complex interventions: case study (CLUSTER). BMC medical research methodology 2013: 13:118.

Burchett H, Umoquit M, Dobrow M. How do we know when research from one setting can be useful in another? A review of external validity, applicability and transferability frameworks. J Health Serv Res Policy 2011: 16:238-44.

Centre for Reviews and Dissemination (CRD). CRD’s Guidance for Undertaking Reviews in Health Care. University of York, 2009.

Cochrane Collaboration, Cochrane Handbook for Systematic Reviews of Interventions, Cochrane Collaboration, Version 5.1.0 March 2011. Available from http://www.cochrane-handbook.org. Accessed 3/7/15.

Connell J. Kubisch A. Applying a theory of change approach to the evaluation of comprehensive community initiatives: progress, prospects and problems. In New Approaches to Evaluating Community Initiatives, Vol 2: Theory, Measurement and Analysis. Ed K. Fullbright-Anderson, A. Kubisch, J. Connell 15-44, Queenstown: The Aspen Institute, 1998.

Davies, G. P., Williams, A. M., Larsen, K., Perkins, D., Roland, M., & Harris, M. F. (2008). Coordinating primary health care: an analysis of the outcomes of a systematic review. Medical Journal of Australia 2008; 188: S65.

Davies, G., Harris, M., Perkins, D., Roland, M., Williams, A., Larsen, K., & McDonald, J. Coordination of care within primary health care and with other sectors: a systematic review. Australian Primary Health Care Research Institute Report, 2006.

Dyson A, Todd L. Dealing with complexity: theory of change evaluation and the full service extended schools initiative. *International Journal of Research and Method in Education* 2010; 33: 119-134.

Foss Hansen H. Choosing evaluation models: a discussion on evaluation design. Evaluation 2005: 11; 447-462.

Handler A, Issel M, Turnock B. A conceptual framework to measure performance of the public health system. American Journal of Public Health 2001; 91:1235-1239.

Hoogendoorn WE, van Poppel MNM, Bongers PM, Koes BW, Bouter LM. Physical load during work and leisure time as risk factors for back pain. Scand J Work Environ Health 1999; 25(5):387-403.

Joly B, Polyak G, Davis M, Brewster J, Tremain B, Raevsky C, Beitsch L. Linking Accreditation and Public Health Outcomes: A Logic Model Approach. Journal of Public Health Management Practice, 2007;13: 349–356.

Ham C, Murray R. Implementing the NHS five year forward view: aligning policies with the plan. London: Kings Fund, 2015.

Long T, Khan AM, Chana N. Achieving better value: primary care must lead on population health. Postgraduate Medical Journal 2015; 91:59: doi:10.1136/postgradmedj-2015-133264.

National Health Service Public Health Resource Unit. Critical appraisal skills programme: qualitative research appraisal tool. 2006. Available from: http://www.phru.nhs.uk/Doc_Links/Qualitative%20Appraisal%20Tool.pdf. Accessed 3/7/15.

National Health Service England. The New Care Models Programme NHS England Board Paper: 23 July. 2015. Available from: <http://www.england.nhs.uk/wp-content/uploads/2015/07/Item-3-New-Models-of-Care-Vanguard-Support.pdf>. Accessed 3/7/15.

National Health Service England. Five Year Forward View, NHS England, 2014. [http://www.england.nhs.uk/wp-content/uploads/2014/10/5yfv-web.pdf. Accessed 23 July 2015](http://www.england.nhs.uk/wp-content/uploads/2014/10/5yfv-web.pdf.%20Accessed%2023%20July%202015).

National Health Service England. The forward view into action: New Care Models: update and initial support, NHS England, 2015. <http://www.england.nhs.uk/wp-content/uploads/2015/07/ncm-support-package.pdf>. Accessed 6/8/2015.

Nilsen P. The how and why of community-based injury prevention: a conceptual and evaluation model. *Safety Science* 2007; 45: 501-521.

Ogilvie, D, Fayter D, Petticrew M, Sowden A, Thomas S, Whitehead M, Worthy G. The harvest plot: A method for synthesising evidence about the differential effects of interventions**.** BMC Medical Research Methodology 2008: 8: doi:10.1186/1471-2288-8-8.

Pawson R, Tilley N. Realistic Evaluation. London: Sage, 1997.

Pawson RD. Evidence-based policy: a realist perspective. London: Sage, 2006.

Pawson R, Boaz A, Grayson L, Long A, Barnes C. Types and quality of social care knowledge. Stage two: towards the quality assessment of social care knowledge. London: Social Care Institute for Excellence, 2003.

Rees K, Bennett P, West R, Davey Smith G, Ebrahim S. Psychological interventions for coronary heart disease. Cochrane Database of Systematic Reviews 2004, 2, CD002902. DOI: 10.1002/14651858.

Robertson, H. Integration of health and social care: A review of literature and models Implications for Scotland. Edinburgh: Royal College of Nursing Scotland, 2011.

Rogers P. Using programme theory to evaluate complicated and complex aspects of interventions. Evaluation 2008; 14: 29-48.

Rossi P, Lipsey M, Freeman H. Evaluation – A Systematic Approach, Thousand Oaks CA: Sage, 2004.

Shortell SM, Addicott R, Walsh N, Ham C. The NHS five year forward view: lessons from the United States in developing new care models. The BMJ 2015;350:h2005. doi:10.1136/bmj.h2005.

Torjesen I. Roundtable: Leading the way in developing new care models 2015. <http://www.hsj.co.uk/resource-centre/supplements/roundtable-leading-the-way-in-developing-new-care-models/5084817.article#.VbO5cflVhHw>. Accessed 25 July 2015.

Weiss C. Theory-based evaluation: past, present and future. New Directions for Evaluation 2007; 76: 68-81.

**Appendix S2 Completed PRISMA checklist**

| **Section/topic** | **#** | **Checklist item** | **Reported on page #** |
| --- | --- | --- | --- |
| **TITLE** | | |  |
| Title | 1 | Identify the report as a systematic review, meta-analysis, or both. | 1 |
| **ABSTRACT** | | |  |
| Structured summary | 2 | Provide a structured summary including, as applicable: background; objectives; data sources; study eligibility criteria, participants, and interventions; study appraisal and synthesis methods; results; limitations; conclusions and implications of key findings; systematic review registration number. | 2 |
| **INTRODUCTION** | | |  |
| Rationale | 3 | Describe the rationale for the review in the context of what is already known. | 3 |
| Objectives | 4 | Provide an explicit statement of questions being addressed with reference to participants, interventions, comparisons, outcomes, and study design (PICOS). | 5 |
| **METHODS** | | |  |
| Protocol and registration | 5 | Indicate if a review protocol exists, if and where it can be accessed (e.g., Web address), and, if available, provide registration information including registration number. | 2,4 |
| Eligibility criteria | 6 | Specify study characteristics (e.g., PICOS, length of follow-up) and report characteristics (e.g., years considered, language, publication status) used as criteria for eligibility, giving rationale. | 5 |
| Information sources | 7 | Describe all information sources (e.g., databases with dates of coverage, contact with study authors to identify additional studies) in the search and date last searched. | 4 |
| Search | 8 | Present full electronic search strategy for at least one database, including any limits used, such that it could be repeated. | App 1 |
| Study selection | 9 | State the process for selecting studies (i.e., screening, eligibility, included in systematic review, and, if applicable, included in the meta-analysis). | 5 |
| Data collection process | 10 | Describe method of data extraction from reports (e.g., piloted forms, independently, in duplicate) and a26ny processes for obtaining and confirming data from investigators. | 5 |
| Data items | 11 | List and define all variables for which data were sought (e.g., PICOS, funding sources) and any assumptions and simplifications made. | 5 |
| Risk of bias in individual studies | 12 | Describe methods used for assessing risk of bias of individual studies (including specification of whether this was done at the study or outcome level), and how this information is to be used in any data synthesis. | 6 |
| Summary measures | 13 | State the principal summary measures (e.g., risk ratio, difference in means). | 6 |
| Synthesis of results | 14 | Describe the methods of handling data and combining results of studies, if done, including measures of consistency (e.g., I^2^) for each meta-analysis. | 6 |

Page 1 of 2

| **Section/topic** | **#** | **Checklist item** | **Reported on page #** |
| --- | --- | --- | --- |
| Risk of bias across studies | 15 | Specify any assessment of risk of bias that may affect the cumulative evidence (e.g., publication bias, selective reporting within studies). | 7 |
| Additional analyses | 16 | Describe methods of additional analyses (e.g., sensitivity or subgroup analyses, meta-regression), if done, indicating which were pre-specified. | 7 |
| **RESULTS** | | |  |
| Study selection | 17 | Give numbers of studies screened, assessed for eligibility, and included in the review, with reasons for exclusions at each stage, ideally with a flow diagram. | 7 |
| Study characteristics | 18 | For each study, present characteristics for which data were extracted (e.g., study size, PICOS, follow-up period) and provide the citations. | 8 |
| Risk of bias within studies | 19 | Present data on risk of bias of each study and, if available, any outcome level assessment (see item 12). | 9 |
| Results of individual studies | 20 | For all outcomes considered (benefits or harms), present, for each study: (a) simple summary data for each intervention group (b) effect estimates and confidence intervals, ideally with a forest plot. | 11-15 |
| Synthesis of results | 21 | Present results of each meta-analysis done, including confidence intervals and measures of consistency. | n/a |
| Risk of bias across studies | 22 | Present results of any assessment of risk of bias across studies (see Item 15). | 9 |
| Additional analysis | 23 | Give results of additional analyses, if done (e.g., sensitivity or subgroup analyses, meta-regression [see Item 16]). | n/a |
| **DISCUSSION** | | |  |
| Summary of evidence | 24 | Summarize the main findings including the strength of evidence for each main outcome; consider their relevance to key groups (e.g., healthcare providers, users, and policy makers). | 9-15 |
| Limitations | 25 | Discuss limitations at study and outcome level (e.g., risk of bias), and at review-level (e.g., incomplete retrieval of identified research, reporting bias). | 18-19 |
| Conclusions | 26 | Provide a general interpretation of the results in the context of other evidence, and implications for future research. | 19-20 |
| **FUNDING** | | |  |
| Funding | 27 | Describe sources of funding for the systematic review and other support (e.g., supply of data); role of funders for the systematic review. | 21 |

*From:*  Moher D, Liberati A, Tetzlaff J, Altman DG, The PRISMA Group (2009). Preferred Reporting Items for Systematic Reviews and Meta-Analyses: The PRISMA Statement. PLoS Med 6(7): e1000097. doi:10.1371/journal.pmed1000097

For more information, visit: **www.prisma-statement.org**.

Page 2 of 2

**Appendix S3**

**Search strategy**

**MEDLINE, MEDLINE In-Process & Other Non-Indexed Citations, Epub Ahead of Print, MEDLINE ® without Revisions via OvidSP**

1 (care adj1 model*).ti,ab.

2 new.ti,ab.

3 1 and 2

4 (model* adj1 service delivery).ti,ab.

5 (model* adj1 (healthcare or health care or health-care)).ti,ab.

6 (transform* adj1 (service* or care)).ti,ab.

7 ("integration of care" or integrated care).ti,ab.

8 (integrated system* and health).ti,ab.

9 (vanguard* and (health or service*)).ti,ab.

10 accountable care.ti,ab.

11 (future proof* or future-proof* or futureproof*).ti,ab.

12 (reform* adj (health or service* or care or healthcare)).ti,ab.

13 (service* adj1 redesign*).ti,ab.

14 "five year forward view".ti,ab.

15 ((health or healthcare or service*) adj reform*).ti.

16 or/3-15

17 *Health Care Reform/

18 *Organizational Innovation/

19 *Quality Improvement/

20 *Health Priorities/

21 *Accountable Care Organizations/

22 *"Delivery of Health Care, Integrated"/

23 (og or td).fs.

24 or/17-22

25 23 and 24

26 (health service* or healthcare or health care or model*).ti,ab.

27 25 and 26

28 16 or 27

29 limit 28 to english language

30 limit 29 to yr=”2006-Current”

**Search Filters:**

• To retrieve Systematic Reviews the following search filter was combined with (AND) line 30 of the above search strategy: Scottish Intercollegiate Guidelines Network (SIGN). Search Filters: Systematic Reviews. Available from: http://www.sign.ac.uk/assets/search-filters-systematic-reviews.docx [Accessed 5th July 2017]

• To retrieve UK primary studies the following terms were combined with (AND) line 30 of the above search strategy: exp Great Britain/ OR (Britain or british or wales or welsh or Scottish or scots or Scotland or England or English or Birmingham or leeds or London or Liverpool or Manchester or Glasgow or Edinburgh or Cardiff or Belfast or UK or GB or aberdeen).ti,ab,in,hw.

**EMBASE via OvidSP**

1 (care adj1 model*).ti,ab.

2 new.ti,ab.

3 1 and 2

4 (model* adj1 service delivery).ti,ab.

5 (model* adj1 (healthcare or health care or health care)).ti,ab.

6 (transform* adj1 (service* or care)).ti,ab.

7 ("integration of care" or integrated care).ti,ab.

8 (integrated system* and health).ti,ab.

9 (vanguard* and (health or service*)).ti,ab.

10 accountable care.ti,ab.

11 (future proof* or future-proof* or futureproof*).ti,ab.

12 (reform* adj (health or service* or care or healthcare)).ti,ab.

13 (service* adj1 redesign*).ti,ab.

14 "five year forward view".ti,ab.

15 ((health or healthcare or service*) adj reform*).ti.

16 or/3-15

17 *Health Care Reform/

18 *Organizational Innovation/

19 *Quality Improvement/

20 *Health Priorities/

21 *Accountable Care Organizations/

22 *"Delivery of Health Care, Integrated"/

23 (og or td).fs.

24 or/17-22

25 23 and 24

26 (health service* or healthcare or health care or model*).ti,ab.

27 25 and 26

28 16 or 27

29 limit 28 to embase

30 limit 29 to english language

31 limit 30 to yr=”2006-Current”

**Search Filters:**

• To retrieve Systematic Reviews the following search filter was combined with (AND) line 31 of the above search strategy: Scottish Intercollegiate Guidelines Network (SIGN). Search Filters: Systematic Reviews. Available from: http://www.sign.ac.uk/assets/search-filters-systematic-reviews.docx [Accessed 5th July 2017]

• To retrieve UK primary studies the following terms were combined with (AND) line 31 of the above search strategy: exp Great Britain/ OR (Britain or british or wales or welsh or Scottish or scots or Scotland or England or English or Birmingham or leeds or London or Liverpool or Manchester or Glasgow or Edinburgh or Cardiff or Belfast or UK or GB or aberdeen).ti,ab,in,hw.

**CINAHL via EBSCO**

S1 TI (care n1 model*) OR AB (care n1 model*)

S2 TI new OR AB new

S3 S1 AND S2

S4 TI (model* n1 service delivery) OR AB (model* n1 service delivery)

S5 TI ( (model* n1 (healthcare or health care or health-care)) ) OR AB ( (model* n1 (healthcare or health care or health-care)) )

S6 TI ( (transform* n1 (service* or care)) ) OR AB ( (transform* n1 (service* or care)) )

S7 TI ( ("integration of care" or integrated care) ) OR AB ( ("integration of care" or integrated care) )

S8 TI ( (integrated system* and health) ) OR AB ( (integrated system* and health) )

S9 TI ( (vanguard* and (health or service*)) ) OR AB ( (vanguard* and (health or service*)) )

S10 TI accountable care OR AB accountable care

S11 TI ( (future proof* or future-proof* or futureproof*) ) OR AB ( (future proof* or future-proof* or futureproof*) )

S12 TI ( (reform* n1 (health or service* or care or healthcare)) ) OR AB ( (reform* n1 (health or service* or care or healthcare)) )

S13 TI (service* n1 redesign*) OR AB (service* n1 redesign*)

S14 TI "five year forward view" OR AB "five year forward view"

S15 S4 OR S5 OR S6 OR S7 OR S8 OR S9 OR S10 OR S11 OR S12 OR S13 OR S14

S16 S3 OR S15

S17 (MM "Health Care Reform")

S18 (MM "Diffusion of Innovation") OR (MM "Organizational Change")

S19 (MM "Quality Improvement")

S20 (MM "Accountable Care Organizations")

S21 (MM "Health Care Delivery, Integrated")

S22 MW OG or TD

S23 S17 OR S18 OR S19 OR S20 OR S21

S24 S22 AND S23

S25 TI ( (health service* or healthcare or health care or model*) ) OR AB ( (health service* or healthcare or health care or model*) )

S26 S24 AND S25

S27 S16 OR S26

**Search Filters:**

• To retrieve Systematic Reviews the following search filter was combined with (AND) line S27 of the above search strategy: Scottish Intercollegiate Guidelines Network (SIGN). Search Filters: Systematic Reviews. Available from: http://www.sign.ac.uk/assets/search-filters-systematic-reviews.docx [Accessed 5th July 2017]

- To retrieve UK primary studies the following terms were combined with (AND) line S27 of the above search strategy: (MH "Great Britain") OR (Britain or british or wales or welsh or Scottish or scots or Scotland or England or English or Birmingham or leeds or London or Liverpool or Manchester or Glasgow or Edinburgh or Cardiff or Belfast or UK or GB or aberdeen)

• Search results were refined to English language and 2006-2016 publication date.

**PyscINFO via OvidSP**

1 (care adj1 model*).ti,ab.

2 new.ti,ab.

3 1 and 2

4 (model* adj1 service delivery).ti,ab.

5 (model* adj1 (healthcare or health care or health-care)).ti,ab.

6 (transform* adj1 (service* or care)).ti,ab.

7 ("integration of care" or integrated care).ti,ab.

8 (integrated system* and health).ti,ab.

9 (vanguard* and (health or service*)).ti,ab.

10 accountable care.ti,ab.

11 (future proof* or future-proof* or futureproof*).ti,ab.

12 (reform* adj (health or service* or care or healthcare)).ti,ab.

13 (service* adj1 redesign*).ti,ab.

14 "five year forward view".ti,ab.

15 ((health or healthcare or service*) adj reform*).ti.

16 or/3-15

17 *Health Care Reform/

18 *Organizational Innovation/

19 *Integrated Services/

20 17 or 18 or 19

21 (health service* or healthcare or health care or model*).ti,ab.

22 20 and 21

23 16 or 22

24 limit 23 to english language

25 limit 24 to yr=”2006-Current”

**Search Filters:**

• To retrieve Systematic Reviews the following search filter was combined with (AND) line 25 of the above search strategy: The University of Texas School of Public Health. Search Filters for Various Databases: Ovid PsycINFO (Systematic reviews and meta-analyses). Available from: http://libguides.sph.uth.tmc.edu/search_filters/ovid_psycinfo_filters [Accessed 5th July 2017].

● To retrieve UK primary studies the following terms were combined with (AND) line 25 of the above search strategy: (Britain or british or wales or welsh or Scottish or scots or Scotland or England or English or Birmingham or leeds or London or Liverpool or Manchester or Glasgow or Edinburgh or Cardiff or Belfast or UK or GB or aberdeen).ti,ab,in,hw.

**The Cochrane Library (DARE, CDSR, CENTRAL, HTA)**

#1 (care near/1 model*):ti,ab,kw (Word variations have been searched)

#2 new:ti,ab

#3 #1 and #2

#4 (model* near/1 service delivery):ti,ab

#5 (model* near/1 (healthcare or health care or health-care)):ti,ab

#6 (transform* near/1 (service* or care)):ti,ab

#7 ("integration of care" or integrated care):ti,ab

#8 (integrated system* and health):ti,ab

#9 (vanguard* and (health or service*)):ti,ab

#10 accountable care:ti,ab

#11 (future proof* or future-proof* or futureproof*):ti,ab

#12 (reform* next (health or service* or care or healthcare)):ti,ab

#13 (service* near/1 redesign*):ti,ab

#14 "five year forward view":ti,ab

#15 ((health or healthcare or service*) next reform*):ti

#16 ^318-#15^

#17 MeSH descriptor: [Health Care Reform] this term only

#18 MeSH descriptor: [Organizational Innovation] this term only

#19 MeSH descriptor: [Quality Improvement] this term only

#20 MeSH descriptor: [Health Priorities] this term only

#21 MeSH descriptor: [Accountable Care Organizations] this term only

#22 MeSH descriptor: [Delivery of Health Care, Integrated] this term only

#23 Any MeSH descriptor with qualifier(s): [Organization & administration - OG]

#24 Any MeSH descriptor with qualifier(s): [Trends - TD]

#25 ^10-#22^

#26 #24 and #25

#27 (health service* or healthcare or health care or model*):ti,ab

#28 #26 and #27

#29 #16 or #28 Publication Year from 2006 to 2016

#30 MeSH descriptor: [Great Britain] explode all trees

#31 (Britain or british or wales or welsh or Scottish or scots or Scotland or England or English or Birmingham or leeds or London or Liverpool or Manchester or Glasgow or Edinburgh or Cardiff or Belfast or UK or GB or aberdeen)

#32 #30 or #31

#33 #29 and #32 – UK primary studies

#34 #29 not #32 - Reviews

**Search Filters:**

No search filters were applied to The Cochrane Library searches.

**Science Citation Index & Social Sciences Citation Index via Web of Science**

#1 TOPIC: (care near/1 model*)

#2 TOPIC: (new)

#3 #2 AND #1

#4 TOPIC: (model* near/1 "service delivery")

#5 TOPIC: (model* near/1 ("healthcare" or "health care" or "health-care"))

#6 TOPIC: ((transform* near/1 (service* or care)))

#7 TOPIC: (("integration of care" or integrated care))

#8 TOPIC: ((integrated system* and health))

#9 TOPIC: ((vanguard* and (health or service*)))

#10 TOPIC: (accountable care)

#11 TOPIC: ((future proof* or future-proof* or futureproof*))

#12 TOPIC: ((reform* near (health or service* or care or healthcare)))

#13 TOPIC: ((service* near/1 redesign*))

#14 TOPIC: (five year forward view)

#15 TITLE: (((health or healthcare or service*) near reform*))

#16 #15 OR #14 OR #13 OR #12 OR #11 OR #10 OR #9 OR #8 OR #7 OR #6 OR #5 OR #4 OR #3

**Search Filters:**

Methodological search filters are not available for Web of Science, therefore the following approach was used to identify reviews and UK primary studies.

• To retrieve Systematic Reviews the following terms were combined with (AND) line #16 of the above search strategy TITLE: ((meta analysis) or (systematic review)) and the search results were refined to “Review” for Document Type and the Publication Years 2006-2016.

• To retrieve UK primary studies the following terms were combined with (AND) line #16 of the above search strategy TS=((Britain or british or wales or welsh or Scottish or scots or Scotland or England or English or Birmingham or leeds or London or Liverpool or Manchester or Glasgow or Edinburgh or Cardiff or Belfast or UK or United Kingdom or GB or aberdeen)) and the search results were refined to “Article” for Document Type and the Publication Years 2006-2016.

**Appendix S4**

**Studies excluded at full paper review**

**UK studies**

|  | Addicott R: Challenges of commissioning and contracting for integrated care in the National Health Service (NHS) in England. Australian Journal of Primary Health 2016, 22(1):50-54. | Describes features of five case study sites but provides no other data. |
| --- | --- | --- |
|  | Adinolfi P: Barriers to reforming healthcare: the Italian case. Health care analysis : HCA : journal of health philosophy and policy 2014, 22(1):36-58. | General description of reform in Italian healthcare rather than a literature review |
|  | Adomaviciute S, Watt H, Soljak M, Car J, Majeed A: Impact of the Integrated Care Pilot on HbA1c, cholesterol and systolic blood pressure levels in patients with diabetes. Diabetic Medicine 2014, 31:1 | Conference abstract |
|  | Alderwick H, Ham, C; Buck, D.: Population health systems: Going beyond integrated care. London: Kings Fund; 2015. | Provides an overview of the issues, and describes initiatives in other countries. |
|  | Ali S, O'Callaghan V, Middleton JD, Little R: A prospective mini health impact assessment of the 'Towards 2010' programme in Sandwell and West Birmingham in the West Midlands. Public Health 2007, 121(6):469-481. | Describes a desktop exercise to explore projected health impacts (health impact assessment) |
|  | Anderson KJ: A review of health care reform in the United States and in Alaska. International journal of circumpolar health 2010, 69(5):424-436. | General description of US health service reform rather than a review |
|  | Andrews T, Read J: The importance of collaborative theory in older people's services. Journal of Integrated Care 2009, 17(2):35-40. | Describes development of a mental health resource. Outcomes descriptive apart from mention of a high satisfaction rating from service users, that the projected secured funding, the team responds to all referrals within 48 hours. |
|  | Annus C, Kelly C: Evaluation of nurse-led service innovations designing models of integrated care for patients with cardiovascular disease. European Journal of Cardiovascular Nursing 2014, 13:S85-S86. | Conference abstract |
|  | Archie, S. Integrated care improves one year outcomes in first episode psychosis. Evidence-based mental health 2006; 9 (6) 46 | Brief (half a page) summary of non-UK study. |
|  | Back A, Park E, Greer J, Jackson V, Jacobsen J, Gallagher E, Temel J: Clinician roles in early integrated palliative care for patients with advanced cancer: a qualitative study. Journal of palliative medicine 2014, 17(11):1244-1248. | Describes clinician role in care provision for patients rather than integrated care |
|  | Balatsoukas P, Williams R, Davies C, Ainsworth J, Buchan I. User Interface Requirements for Web-Based Integrated Care Pathways: Evidence from the Evaluation of an Online Care Pathway Investigation Tool. Journal of Medical Systems. 2015;39 (11) (no pagination)(183). | Technical rather than service delivery focus |
|  | Bali A, Hargreaves DS, Cowman J, Lakhanpaul M, Dunkley C, Power M, Cross JH: Integrated care for childhood epilepsy: ongoing challenges and lessons for other long-term conditions. Archives of Disease in Childhood 2016, 24:24. | Discussion/overview of literature in the area |
|  | Bamford D, Rothwell K, Tyrrell P, Boaden R: Improving care for people after stroke: how change was actively facilitated. Journal of health organization and management 2013, 27(5):548-560. | Describes the process of developing a new care approach rather than the approach |
|  | Banham SJ: Using a patient-focused approach to improve services in a nurse-led general practice. Nursing times 2009, 105(22):17-19. | Commentary |
|  | Barr PJ, McElnay JC, Hughes CM: Connected health care: the future of health care and the role of the pharmacist. Journal of evaluation in clinical practice 2012, 18(1):56-62. | Describes current and future developments |
|  | Barrett RV: An integrated community team works with the London ambulance service: Perspectives on the emerging role of physiotherapists in pre-hospital care. Physiotherapy (United Kingdom) 2015, 101:eS121. | Conference abstract |
|  | Barton D, Mashlan W: An advanced nurse practitioner-led service - consequences of service redesign for managers and organizational infrastructure. Journal of nursing management 2011, 19(7):943-949. | Describes themes arising from evaluation of the service but provides no data to support the description |
|  | Beech R, Henderson C, Ashby S, Dickinson A, Sheaff R, Windle K, Wistow G, Knapp M: Does integrated governance lead to integrated patient care? Findings from the innovation forum. Health & social care in the community 2013, 21(6):598-605. | Describes patient pathways in existing service delivery rather than a new model |
|  | Bell C, Hashemi N, Wieland F, Lowrey C, Kaur V: ACE impact: evaluation of an integrated geriatric service...Acute Care of the Elderly. Age & Ageing 2014, 43(suppl_2):ii7-ii7. | Conference abstract |
|  | Bell K, Kinder T, Huby G: What comes around goes around: on the language and practice of 'integration' in health and social care in Scotland. Journal of Integrated Care 2008, 16(4):40-48. | Descriptive overview |
|  | Belton J, Sears C: Nurse-led general practice: a new care model. Nursing Times 2015, 111(47):12-13. | Descriptive evaluation of the model only with no data |
|  | Bender M, Williams M, Su W, Hites L: Refining and validating a conceptual model of Clinical Nurse Leader integrated care delivery. Journal of Advanced Nursing 2016, 24:24. | Describes the development of a model of clinical nurse leadership rather than exploring integrated care delivery |
|  | Bernard S, Aspinal F, Gridley K, Parker G: Integrated policy making in England for adults with long-term neurological conditions (LTNCs): some preliminary findings from a scoping study. International Journal of Integrated Care 2008, 8:e60. | Describes the implementation of the national service framework and reports data regarding the perception of impact of policy on patients |
|  | Bion J, Evans T: The influence of health care reform on intensive care: a UK perspective. American journal of respiratory and critical care medicine 2011, 184(10):1093-1094. | Editorial discussion |
|  | Birrell D, Heenan D. Implementing the Transforming Your Care agenda in Northern Ireland within integrated structures. Journal of Integrated Care. 2012;20(6):359-66. | Descriptive overview |
|  | Bladin C, Fullerton S, Chapman M, Ryan C. Introduction of the Liverpool care pathway for the dying patient improved the care of dying patients and their families in an acute stroke unit. International Journal of Stroke. 2009;4:23. | Conference abstract |
|  | Bourke SJ, Doe SJ, Gascoigne AD, Heslop K, Fields M, Reynolds D, Mannix K: An integrated model of provision of palliative care to patients with cystic fibrosis. Palliative medicine 2009, 23(6):512-517. | Describes clinical care of patients, no data relating to delivery of the service |
|  | Bridges J, Meyer J: Policy on new workforce roles: a discussion paper. International journal of nursing studies 2007, 44(4):635-644. | Descriptive overview |
|  | Brownsell S, Aldred H, Young T, Hawley M. Reforming health care through information and communication technologies. Journal of Care Service Management. 2008;2(3):286-300. | Relates to clinical use of ICT rather than a new model of care |
|  | Burden ACF: Diabetes service redesign: Useful lessons. Practical Diabetes International 2009, 26, 11 | Commentary |
|  | Burton CR, Payne S: Integrating palliative care within acute stroke services: developing a programme theory of patient and family needs, preferences and staff perspectives. Bmc Palliative Care 2012, 11. | Relates to integrating palliative and stroke care rather than integrating services |
|  | Busse R, Stahl J: Integrated care experiences and outcomes in Germany, the Netherlands, and England. Health Affairs 2014, 33(9):1549-1558. | Describes the programmes and provides an overview of evidence. Useful background but not a primary study or a systematic review. |
|  | Campbell SM, Kontopantelis E, Reeves D, Valderas JM, Gaehl E, Small N, Roland MO: Changes in patient experiences of primary care during health service reforms in England between 2003 and 2007. Annals of family medicine 2010, 8(6):499-506. | Relates to general NHS reforms rather than new models of care |
|  | Candy B, Taylor SJC, Ramsay J, Esmond G, Griffiths CJ, Bryar RM: Service implications from a comparison of the evidence on the effectiveness and a survey of provision in England and Wales of COPD specialist nurse services in the community. International journal of nursing studies 2007, 44(4):601-610. | Describes a survey of current practice in service provision for COPD |
|  | Chalkley M, McVicar D: Choice of contracts in the British National Health Service: an empirical study. Journal of health economics 2008, 27(5):1155-1167. | Descriptive overview of literature |
|  | Challis D, Stewart K, Donnelly M, Weiner K, Hughes J: Care management for older people: does integration make a difference? Journal of Interprofessional Care 2006, 20(4):335-348. | Describes and compares the extent of integrated care in Northern Ireland and England. |
|  | Checkland K, Harrison S, Marshall M: Is the metaphor of 'barriers to change' useful in understanding implementation? Evidence from general medical practice. Journal of health services research & policy 2007, 12(2):95-100. | Relates to implementation of quality standards/checklist rather than new model of care |
|  | Chestnutt IG, Thomas DR, Patel R, Treasure ET: Perceptions and attitudes to a fundamental reform of general dental services in Wales. Primary dental care : journal of the Faculty of General Dental Practitioners (UK) 2007, 14(1):13-18. | Provides little data of relevance, reports a survey of views of working arrangements |
|  | Cohen MAH, McCarthy P, Khan Y: Integrated care results in fewer elderly people dying in hospital. BMJ (Clinical research ed) 2012, 345:e4731. | Comment only |
|  | Collins CG, Leahy AL: Integrated care pathways in surgery. The surgeon: journal of the Royal Colleges of Surgeons of Edinburgh and Ireland 2008, 6(2):69-70. | Descriptive article with no data |
|  | Connor M, Kissen G: Tackling whole-systems change: the Trafford framework for integrated services. Journal of Integrated Care 2010, 18(3):4-14. | Provides a descriptive overview of the Trafford integrated service work but provides no data. |
|  | Coventry P, Lovell K, Dickens C, Bower P, Chew-Graham C, McElvenny D, Hann M, Cherrington A, Garrett C, Gibbons CJ et al: Integrated primary care for patients with mental and physical multimorbidity: cluster randomised controlled trial of collaborative care for patients with depression comorbid with diabetes or cardiovascular disease. BMJ (Clinical research ed) 2015, 350:h638. | Intervention comprises integrating psychological therapy with other routine GP care rather than a new model of service. Describes clinical outcomes. |
|  | Craig TKJ, Johnson S, McCrone P, Afuwape S, Hughes E, Gournay K, White I, Wanigaratne S, Leese M, Thornicroft G: Integrated care for co-occurring disorders: psychiatric symptoms, social functioning, and service costs at 18 months. Psychiatric services (Washington, DC) 2008, 59(3):276-282. | Describes a training programme for care managers to improve care provided |
|  | Cresswell KM, Worth A, Sheikh A: Integration of a nationally procured electronic health record system into user work practices. Bmc Medical Informatics and Decision Making 2012, 12. | Describes challenges in implementing electronic record systems generally rather than focusing on integrating systems. |
|  | Crilly T, Plant M. Reforming emergency care: Primary Care Trust power in action research. Health services management research : an official journal of the Association of University Programs in Health Administration / HSMC, AUPHA. 2007;20(1):37-47. | Models the flow of patients rather than providing outcomes |
|  | Currie G, Finn R, Martin G. Accounting for the 'dark side' of new organizational forms: the case of healthcare professionals. Human Relations 2008 n61 (4) 539e - 564 | Focus on employment relations within networks. |
|  | Dalley C, Basarir H, Wright JG, Fernando M, Pearson D, Ward SE, Thokula P, Krishnankutty A, Wilson G, Dalton A et al: Specialist integrated haematological malignancy diagnostic services: an Activity Based Cost (ABC) analysis of a networked laboratory service model. Journal of clinical pathology 2015, 68(4):292-300. | Describes use of an economic model for evaluating laboratory services, refers to different models but the emphasis is on the methodology rather than cost effectiveness of the models. |
|  | Davies C: The promise of 21st century professionalism: regulatory reform and integrated care. Journal of interprofessional care 2007, 21(3):233-239. | Editorial |
|  | de Silva D, Haririan S: Service redesign: Joint benefits of streamlined care. The Health service journal 2012, 122(6290):20-22. | Commentary |
|  | Doherty, C. A qualitative study of health service reform on nurses' working lives: learning from the UK National Health Service (NHS). International journal of nursing studies 2009: 46 (8) 1134-42 | About general change rather than new models of care. |
|  | Duarte A, Walker J, Walker S, Richardson G, Hansen CH, Martin P, Murray G, Sculpher M, Sharpe M: Cost-effectiveness of integrated collaborative care for comorbid major depression in patients with cancer. Journal of Psychosomatic Research 2015, 79(6):465-470. | The intervention is a multi-component treatment programme rather than a new model of care |
|  | Eason K, Waterson P: The implications of e-health system delivery strategies for integrated healthcare: lessons from England. International journal of medical informatics 2013, 82(5):e96-e106. | General description only with no data |
|  | Eaton M: Embedding change in the healthcare sector. Perspectives in public health 2014, 134(1):12-13. | General overview of change processes |
|  | Egbunike JN, Shaw C, Porter A, Button LA, Kinnersley P, Hood K, Bowden S, Bale S, Snooks H, Edwards A: Streamline triage and manage user expectations: lessons from a qualitative study of GP out-of-hours services. The British journal of general practice : the journal of the Royal College of General Practitioners 2010, 60(572):e83-97. | Evaluates GP service only rather than integration across services |
|  | Elwell R: Developing a nurse-led integrated 'red legs' service. British journal of community nursing 2014, 19(1):12-19. | New clinical service rather than new model of care |
|  | Ewing CI, Cropper SA, Horsburgh TB: Developing, implementing and evaluating integrated care models for infants, children, young people and their families. Archives of Disease in Childhood 2016, 101(9):781-782. | Discusses questions which should be asked when evaluating integrated care |
|  | Fayers K, Price H: Successful recommissioning of community diabetes services in West Hampshire. British Journal of Diabetes and Vascular Disease 2015, 15(3):127-130. | Describes the development of the service but provides no data |
|  | Featherstone I, Keen J: Do integrated record systems lead to integrated services? An observational study of a multi-professional system in a diabetes service. International journal of medical informatics 2012, 81(1):45-52. | Little data provided, predominantly related to different professionals’ use of the records rather than integrating care |
|  | Fiandeiro C, Gibson N, Giannitopoulos G, Zoumprouli A. Management of patients with spinal cord injury: An integrated care pathway. Journal of Neurosurgical Anesthesiology. 2015;27 (4):379-80. | Conference abstract |
|  | Fifield L, Blake S: The early intervention safeguarding nurse pilot: an integrated model of working. Community practitioner: the journal of the Community Practitioners' & Health Visitors' Association 2011, 84(11):27-31. | Describes the development of a team which includes a health visitor and nurse in addition to social services staff. |
|  | Finn R, Learmonth M, Reedy P: Some unintended effects of teamwork in healthcare. Social Science & Medicine 2010, 70(8):1148-1154. | Ethnographic study of teamworking, focus on exploration of teamworking and identity. |
|  | Fletcher A, Worthington D: What is a 'generic' hospital model?--a comparison of 'generic' and 'specific' hospital models of emergency patient flows. Health care management science 2009, 12(4):374-391. | Describes literature around different economic models that can be used for modelling emergency services |
|  | Fox A. A new model for care and support: sharing lives and taking charge. Working with Older People: Community Care Policy & Practice 2011; 15 (2) 58-63 | Description; no methods or data |
|  | Gage H, Dickinson A, Victor C, Williams P, Cheynel J, Davies SL, Iliffe S, Froggatt K, Martin W, Goodman C: Integrated working between residential care homes and primary care: a survey of care homes in England. BMC geriatrics 2012, 12:71. | Reports a survey of existing practice |
|  | Goodwin N: Are networks the answer to achieving integrated care? Journal of health services research & policy 2008, 13(2):58-60. | Editorial |
|  | Graham O, Jayadeva P, Guthrie K: The use of an integrated care pathway for evidence-based practice and clinical governance in abortion care. Journal of obstetrics and gynaecology : the journal of the Institute of Obstetrics and Gynaecology 2010, 30(4):397-403. | Reports clinical outcomes only |
|  | Graves H, Pollard LC, Lempp H, Kingsley GH, Scott DL: Perceived barriers to integrated care in rheumatoid arthritis (RA): Views of providers and recipients of primary and secondary care services. Rheumatology 2009, 48:i107. | Conference abstract |
|  | Greaves, F. Pappas, Y. Bardsley, M. Harris, M Curry, N. et al. Evaluation of complex integrated care programmes: the approach in North West London. International Journal of Integrated Care 2013 e006 | Study protocol |
|  | Gregory M: Developing a Patient Care Co-ordination Centre in Trafford, England: lessons from the International Foundation for Integrated Care (IFIC)/Advancing Quality Alliance integrated care fellowship experience. International Journal of Integrated Care 2015, 15:e009. | Describes plans for development of the new centre based on discussion of models in other countries |
|  | Griffiths C, Miles K, Penny N, George B, Stephenson J, Power R, Twist P, Brough G, Edwards SG: A formative evaluation of the potential role of nurse practitioners in a central London HIV outpatient clinic. AIDS Care 2006, 18(1):22-26. | Focus of the paper is on views regarding the need for potential role change, mentions the need for training however, nothing else of relevance |
|  | Guleri A, More R, Roberts D, Zacharias J, Tang A, Waddington N, et al. Integrated care pathway for management of infective endocarditis - An innovative multidisciplinary approach: clinical outcomes and experience from the Lancashire cardiac centre, UK. Clinical Microbiology and Infection. 2011;17:S353-S4. | Conference abstract |
|  | Guven-Uslu P: Uncertainty and commitment in commissioning of health services. Public Money & Management 2012, 32(5):349-356. | Describes challenges in PCT funding processes, mentions that these difficulties will impact on service integration but focus is not on new models |
|  | Hall I, Parkes C, Samuels S, Hassiotis A: Working across boundaries: clinical outcomes for an integrated mental health service for people with intellectual disabilities. Journal of intellectual disability research : JIDR 2006, 50(Pt 8):598-607. | Exclusively clinical measures |
|  | Hall JL, van Teijlingen ER: A qualitative study of an integrated maternity, drugs and social care service for drug-using women. BMC Pregnancy & Childbirth 2006, 6:19. | Focus on perceptions of care rather than new ways of service delivery |
|  | Hamilton S, Manthorpe J, Szymczynska P, Clewett N, Larsen J, Pinfold V, Tew J: Implementing personalisation in integrated mental health teams in England. Journal of Interprofessional Care 2015, 29(5):488-493. | Focus of the paper is on how professional role issues impact on providing personalised budgets for service users, rather than integration of services |
|  | Harden PN, Walsh G, Bandler N, Bradley S, Lonsdale D, Taylor J, Marks SD: Bridging the gap: an integrated paediatric to adult clinical service for young adults with kidney failure. BMJ (Clinical research ed) 2012, 344:e3718. | Reports only clinical outcomes from a new clinic for young people |
|  | Harris M, Greaves F Patterson S, Jones J, Pappas Y, Majeed A, Car J. The North West London Integrated Care Pilot: innovative strategies to improve care coordination for older adults and people with diabetes. The Journal of ambulatory care management 2012; 35 (3), 216-225 | Description; no methods or data |
|  | Hatfield B, Sharma I, Ryan T. Changing the Focus of Community Mental Health Teams: A Study in one English Locality. Journal of Integrated Care. 2007;15(3):17-28. | Data relate to characteristics of service users rather than service delivery |
|  | Hawkes N: GP networks could be answer to integrated care, report says. BMJ (Clinical research ed) 2014, 348:g1652. | Commentary |
|  | Hawkes N: Hospitals need encouragement to become part of integrated care, report says. BMJ (Clinical research ed) 2013, 346:f2445. | Commentary |
|  | Hawkes N: Only 12 of 211 commissioning groups are acting on integrated care, conference hears. BMJ (Clinical research ed) 2014, 348:g2475. | Commentary |
|  | Heaps K, Marks-Maran D: Integrated end-of-life care services - the Greenwich Care Partnership. European Journal of Palliative Care 2015, 22(2):84-89. | Describes the components of the service. Provides extremely limited data apart from three quotes from patient relatives describing general positive comments and a figure showing place of death. |
|  | Heenan D, Birrell D: Organisational integration in health and social care: some reflections on the Northern Ireland experience. Journal of Integrated Care 2009, 17(5):3-12. | General discussion |
|  | Heightman M, Restrick L, Stern M: Developing the first UK integrated respiratory registrar role in an inner city integrated care organisation. Clinical Medicine 2015, 15 Suppl 3:s29. | Describes the funding of a new type of post |
|  | Henderson EJ, Rubin GP: Development of a community-based model for respiratory care services. BMC health services research 2012, 12:193. | Describes use of a Delphi to develop service standards |
|  | Hewett N, Bax A, Halligan A: Integrated care for homeless people in hospital: an acid test for the NHS? British journal of hospital medicine (London, England : 2005) 2013, 74(9):484-485. | Editorial |
|  | Hunter B, Segrott J: Are clinical pathways 'a good thing'? Reviewing the evidence. MIDIRS Midwifery Digest 2009, 19(4):515-517. | Descriptive overview of the literature |
|  | Hunter B, Segrott J: Using a Clinical Pathway to Support Normal Birth: Impact on Practitioner Roles and Working Practices. Birth-Issues in Perinatal Care 2010, 37(3):227-236. | Focus on clinical care rather than integration |
|  | Huxley P, Evans S, Munroe M, Cestari L: Integrating health and social care in community mental health teams in the UK: a study of assessments and eligibility criteria in England. Health & social care in the community 2008, 16(5):476-482. | Survey of levels of staff agreement on patient need |
|  | Iacobucci G: Hospitals must take lead on new care models and work better with GPs, think tank says. BMJ (Clinical research ed) 2015, 350:h1512. | Commentary |
|  | Iacobucci G: Integrated care scheme for older people and people with diabetes has not reduced emergency admissions in its first year. BMJ (Clinical research ed) 2013, 346:f3255. | Commentary |
|  | Ingleton C, Payne S, Sargeant A, Seymour J: Barriers to achieving care at home at the end of life: transferring patients between care settings using patient transport services. Palliative medicine 2009, 23(8):723-730. | Not related to new model of care |
|  | Ivbijaro GO, Enum Y, Khan AA, Lam SSK, Gabzdyl A. Collaborative Care: Models for Treatment of Patients with Complex Medical-Psychiatric Conditions. Current Psychiatry Reports 2014, 16 11 | Discussion – no data |
|  | Jack BA, Bray L, Kirby J, O'Brien M, Brown J, Swift J, Leigh R, Gavin-Daley A: Modernizing the school health workforce. Staff perceptions of a rapid roll-out redesign programme. Journal of nursing management 2008, 16(6):700-706 | No data of relevance |
|  | Jaganathan, G. Alam, F.Krishna, S. Walton, C. Impact of a new approach "Acute Care Model" on detained patients in adult psychiatry wards in England. European Psychiatry. Conference: 18th European Congress of Psychiatry Munich Germany. Conference Start. 2010 | Conference abstract |
|  | Jarvis A: Accessible and integrated care: Scotland's community nursing review. British journal of community nursing 2007, 12(4):155-157. | Describes a review of services in Scotland |
|  | Johnston B, Buchanan D, Papadopoulou C, Sandeman G, Lord H: Integrating palliative care in lung cancer: an early feasibility study. International journal of palliative nursing 2013, 19(9):433-437. | Data relate to feasibility of carrying out the main study only, no data regarding implementation/applicability |
|  | Jones DEJ, Sutcliffe K, Pairman J, Wilton K, Newton JL: An integrated care pathway improves quality of life in Primary Biliary Cirrhosis. QJM : monthly journal of the Association of Physicians 2008, 101(7):535-543. | Provides only clinical data |
|  | Jones K: Integrated care pilot programme: ensuring people with dementia receive joined up care. Nursing times 2010, 106(10):12-14. | Commentary |
|  | Karakusevic S: Designing an integrated health care system -- what are the key features? Journal of Integrated Care 2010, 18(4):36-42. | Provides a descriptive overview of the South Devon integrated network, highlighting the importance of a systems perspective. The only evaluative information reports that the Trust was shortlisted for awards and ranking in quality indicators. |
|  | Kennedy C, Morioka S: The development of whole-system integrated care in England. Journal of Integrated Care 2014, 22(4):142-153. | Describes the development of a model to support the implementation of integrated care. No data other than limited survey results regarding views of what support is needed. |
|  | Krishna S, Alam, F. Jaganathan, G. Effect of acute care model in provision of general adult psychiatric services. European Psychiatry. Conference: 18th European Congress of Psychiatry Munich Germany. Conference Start. 2010 | Conference abstract |
|  | Kümpers S, Mur I, Hardy B, van Raak A, Maarse H. Integrating dementia care in England and The Netherlands: Four comparative local case studies. Health & Place 2006: 12 (4) 404-420 | Descriptive |
|  | Lai K, Howes K, Butterworth C, Salter M: Lewisham integrated medicines optimisation service: Delivering a system-wide coordinated care model to support patients in the management of medicines to retain independence in their own home. European Journal of Hospital Pharmacy 2015, 22(2):98-101. | Describes the model but provides no data |
|  | Leigh JA, Wild J, Hynes C, Wells S, Kurien A, Rutherford J, Rosen L, Ashcroft T, Hartley V: Transforming community services through the use of a multidimensional model of clinical leadership. Journal of Clinical Nursing 2015, 24(5/6):749-760. | Describes the development of training for clinical leaders |
|  | Lennox A, Anderson ES: Delivering quality improvements in patient care: the application of the Leicester Model of interprofessional education. Quality in primary care 2012, 20(3):219-226. | Discussion paper |
|  | Lewis G, Vaithianathan R, Wright L, Brice MR, Lovell P, Rankin S, Bardsley M: Integrating care for high-risk patients in England using the virtual ward model: lessons in the process of care integration from three case sites. International Journal of Integrated Care 2013, 13. | Compares the intervention and processes across the three sites but provides only description rather than data. |
|  | Liddle A, Wellburn D: Accountable care -- aligning incentives with outcomes. Journal of Integrated Care 2012, 20(3):138-145. | Useful overview of the concept of accountable care organisations, descriptive only |
|  | Limb M: NHS will not improve while new models of care delivery remain "minority interest," conference hears. BMJ (Clinical research ed) 2015, 350:h981. | Commentary |
|  | Lloyd A, Joseph-Williams N, Edwards A, Rix A, Elwyn G: Patchy 'coherence': using normalization process theory to evaluate a multi-faceted shared decision making implementation program (MAGIC). Implementation Science 2013, 8. | Focus is shared decision-making between professionals and patients rather than between practitioners or providers |
|  | Lloyd, T. Steventon, A. Effect of Named, Accountable GPs on Continuity of Care: Protocol for a Regression Discontinuity Study of a National Policy Change. International Journal of Integrated Care 2016; 16(1) | Study protocol |
|  | Lluch M, Abadie F: Exploring the role of ICT in the provision of integrated care-Evidence from eight countries. Health Policy 2013, 111(1):1-13. | Description and some narrative review of the literature. Provides useful background to describe moves towards integrate care across countries. |
|  | Lluch M: Incentives for telehealthcare deployment that support integrated care: a comparative analysis across eight European countries. International Journal of Integrated Care [Electronic Resource] 2013, 13:e042. | Described as a case study using interviews and desk top analysis however, is a descriptive overview of literature in the area. Useful background to the situation and drivers in each country. |
|  | Longwill S, Apea V, Davis P, Ellis S, Scott D, Ncube B, Ault N, Sarner L: Implementation of a nurse-led asymptomatic screening clinic within a London HIV service. HIV Medicine 2010, 11:101-102. | Conference abstract |
|  | Macfarlane DP, Voigt D, Mackie A, Brennan G. Introduction of an integrated care pathway improves management of diabetic ketoacidosis. Diabetic Medicine. 2011;28:191. | Conference abstract |
|  | Main J, Whittle C, Treml J, Woolley J, Main A: The development of an Integrated Care Pathway for all patients with advanced life-limiting illness - the Supportive Care Pathway. Journal of nursing management 2006, 14(7):521-528. | Describes development, very little data relating to evaluation of this pilot |
|  | Mallinson M, King P: Transformation of services and care pathway redesign in the NHS: Further reforms in health policy. Value in Health 2013, 16 (3):A8-A9. | Conference abstract |
|  | Martin GP, Currie G, Finn R, McDonald R: The medium-term sustainability of organisational innovations in the national health service. Implementation science : IS 2011, 6:19. | Study protocol |
|  | Martin GP, Hewitt GJ, Faulkner TA, Parker H: The organisation, form and function of intermediate care services and systems in England: results from a national survey. Health & social care in the community 2007, 15(2):146-154. | Describes characteristics of the current delivery of services |
|  | Mathers N, Thomas M: Integration of care: a bridge too far? The British journal of general practice : the journal of the Royal College of General Practitioners 2012, 62(601):402-403. | Editorial |
|  | McEvoy P, Barnes P: Using the chronic care model to tackle depression among older adults who have long-term physical conditions. Journal of psychiatric and mental health nursing 2007, 14(3):233-238. | Describes the chronic care model, and a case study of application of the model (no data) |
|  | Miller R, Darcy C, Friel A, Scott M, Toner S: The introduction of a new consultant pharmacist case management service on the care of elderly patients in the intermediate care setting. International Journal of Pharmacy Practice 2014, 22:105-106. | Poster |
|  | Mitchell E: Cross-organisational staff liabilities in integrated care settings: the first higher court decision. Journal of Integrated Care 2012, 20(6):367-370. | Description |
|  | Mitchell E: Rising to the challenge: Implications for integrated care of the 'community right to challenge' provisions of the Localism Act 2011. Journal of Integrated Care 2012, 20(4):241-245. | Description |
|  | Mooney H: Service redesign. How regional units take the crisis out of trauma. The Health service journal 2009, 119(6187):22-23. | Commentary |
|  | Morton M, Paice E: Co-Production at the Strategic Level: Co-Designing an Integrated Care System with Lay Partners in North West London, England. International Journal of Integrated Care 2016, 16(2):2. | Describes the involvement of patients in design of the new initiative. It provides learning points and recommendations rather than data. |
|  | Murray J, Young J, Forster A, Herbert G, Ashworth R: Feasibility study of a primary care-based model for stroke aftercare. The British journal of general practice : the journal of the Royal College of General Practitioners 2006, 56(531):775-780. | Describes feasibility of proposed assessments and care delivery for proposed study |
|  | Nelson P, Tabberer S, Chrisp T: Integrated working in children's centres: A user pathway analysis. Practice: Social Work in Action 2011, 23(5):293-310. | Description only with no data |
|  | Nuffield Trust. Evaluation of the first year of the Inner North West London Integrated Care Pilot. London: Nuffield Trust, 2013. | Describes data from the work however, not in detail. The included paper from the same study provides full data. |
|  | Nuno R, Coleman K, Bengoa R, Sauto R: Integrated care for chronic conditions: the contribution of the ICCC Framework. Health policy (Amsterdam, Netherlands) 2012, 105(1):55-64. | Describes a framework |
|  | Parker H, Glasby J: The art of the possible: reforming community health services. British journal of community nursing 2008, 13(10):480-486. | Descriptive overview of the issues |
|  | Parker H, Glasby J: Transforming community health services: English lessons on not relying on organisational reform. Health & social care in the community 2008, 16(5):449-450. | Editorial |
|  | Parkes, Charles, Samuels, Sarah, Hassiotis, Angela, Lynggaard, Henrik, Hall, Ian. Incorporating the views of service users in the development of an integrated psychiatric service for people with learning disabilities. British Journal of Learning Disabilities 2007: 35 (1) 23-29 | More about detailed patient experiences than integration. |
|  | Partridge MR: Integrated care: delivering better outcomes for patients with respiratory disease. British Journal of Healthcare Management 2015, 21(1):7-9. | Describes discussion at a conference regarding integrated care. Provides 2 elements of data from a survey at the conference indicating that members felt that integrated/continuity of care was important for patients. |
|  | Pearce S, Beech N: Challenges and successes in implementing a new Community Care Model. Palliative Medicine 2010, 24(2):247-248. | Conference poster |
|  | Pensuk P: The effectiveness of the liverpool care pathway in end of life care: A systematic review protocol. JBI Library of Systematic Reviews 2013, 11(11):64-76. | Review protocol only |
|  | Procter S,Wilson PM, Brooks F, Kendall, S. Success and failure in integrated models of nursing for long term conditions: Multiple case studies of whole systems. International Journal of Nursing Studies 2013; 50 (5) 632-643 | Descriptive |
|  | Quickfall J, Pollock L. Community nursing: redesign in Scotland. British journal of community nursing. 2008;13(8):373-7. | General redesign rather than integrated care |
|  | Rachev BT: The economics of health service transformation: A business model for care coordination for chronic condition patients in the UK and US. Clinical Governance 2015, 20(3):113-122. | Interesting overview of US models and Trafford UK model but description only. |
|  | Raju K, Siotia R. Acute care model in older peoples mental health services - A service evaluation. European Psychiatry Conference: 20th European Congress of Psychiatry, | Conference abstract |
|  | Raza K, Empson B: Doing things differently: Managing RA in primary care. Rheumatology (United Kingdom) 2014, 53:i2-i3. | Conference abstract |
|  | Renedo A, Marston C. Developing patient-centred care: an ethnographic study of patient perceptions and influence on quality improvement. Bmc Health Services Research. 2015;15. | Focus on quality improvement rather than new model of care |
|  | Richards, David A. Lankshear, Annette J. Fletcher, Janine  Rogers, Anne, Barkham, Michael et al. Developing a U.K. protocol for collaborative care: a qualitative study. General hospital psychiatry 2006; 28 (4) 296-305 | Mainly clinical outcomes |
|  | Richfield E, Adams D, Jones E, Campbell C, Johnson M: 28 Specialist palliative care for parkinson's disease: experiences of a novel integrative service. Age & Ageing 2014, 43(suppl_1):i7-i7. | Conference abstract |
|  | Rickenbach MA, Wedderburn C: Follow the yellow brick road: integrated care--can we do better? The British journal of general practice : the journal of the Royal College of General Practitioners 2012, 62(601):e587-589. | Descriptive opinion article |
|  | Ricote L: The value of modernised professional thinking and role re-design in meeting the challenge of future healthcare demand. Journal of Medical Imaging and Radiation Oncology 2009, 53:A161. | Conference abstract |
|  | Roberts NJ, Ward M, Patel IS, Yorke J, Williams J. What skills, experience and training are need to work in integrated respiratory specialist roles and how can we roll these posts out in the UK? Thorax 2014 69 A127 | Conference abstract |
|  | Robinson S, Williams I, Dickinson H, Freeman T, Rumbold B. Priority-setting and rationing in healthcare: Evidence from the English experience. Social Science & Medicine 75 (2012) 2386e2393 | Describes priority setting by PCTs |
|  | Rollings C: Using nurse led clinical commissioning to improve services for patients with diabetes. Nursing times 2010, 106(33):13-14. | Commentary |
|  | Ross C: Reduction in osteoporosis physiotherapy waiting times through service redesign. Osteoporosis International 2010, 21:S508. | Conference abstract |
|  | Rowe, F, Walker, M, Rockliffe, J, Pollock, A. et al. Delivery of high quality stroke and vision care: experiences of UK services. Disability and Rehabilitation 2016; 38 (8): 813-817 | Mainly clinical outcomes |
|  | Samuels, Sarah, Hall, Ian, Parkes, Charles, Hassiotis, Angela. Professional staff and carers' views of an integrated mental health service for adults with learning disabilities. Psychiatric Bulletin 2007; 31 (1) 13-16 | Very little data |
|  | Sewak NPS, Devienne E, Karia R. The hybrid purchaser-provider split in England: Should Europe follow suit? Value in Health 2013 16 (7) A462 | Conference abstract |
|  | Shaw S LR. Towards integrated care in Trafford. London: Nuffield, 2011. | Details the process of setting up the initiative in Trafford. General description with no data. |
|  | Singh I, Ramakrishna S, Williamson K: The Rapid Assessment Interface and Discharge service and its implications for patients with dementia. Clinical Interventions in Aging 2013, 8:1101-1108. | Describes the RAID model and provides a descriptive overview of studies investigating its use and outcomes |
|  | Spence H, Cappleman J: Fostering teamwork in an intermediate care unit. Nursing management 2011, 18(3):20-24. | Describes the initiative however, provides no data |
|  | Spicer J: Integrated care in the UK: variations on a theme? London Journal of Primary Care 2015, 7(3):41-43. | Discusses and compares reforms in Scotland and England |
|  | Steeden A: The integrated care pilot in north west london. London Journal of Primary Care 2012, 5(1):8-11. | Background description of the pilot and some descriptive data only |
|  | Stewart A, MacIntyre G, Care management in the twenty-first century. Journal of Integrated Care 2013, 21 (2): 91 – 104. | Describes carrying out interviews and focus groups but provides only general description of findings and no data. |
|  | Sutcliffe C, Hughes J, Abendstern M, Clarkson P, Challis D: Developing multidisciplinary assessment--exploring the evidence from a social care perspective. International Journal of Geriatric Psychiatry 2008, 23(12):1297-1305. | Date relate to the impact of a single assessment process on clinical needs and outcomes |
|  | Thomas K, Rowlands MS, Wilson JA: PA18 Heart of gold. integrating cross boundary care across different sectors with patients at the heart of care - a population-based public health perspective. BMJ supportive & palliative care 2015, 5 Suppl 1:A25. | Conference abstract |
|  | Thomas P, Meads G, Moustafa A, Nazareth I, Stange KC, Hess GD: Combined horizontal and vertical integration of care: A goal of practice-based commissioning. Quality in Primary Care 2008, 16(6):425-432. | Discusses different models, useful overview. |
|  | Tomlinson J, Heazell A, Martindale E. An integrated care pathway improves care for families who experience a stillbirth. On behalf of Greater Manchester, Lancashire and South Cumbria special interest group into stillbirth. BJOG: An International Journal of Obstetrics and Gynaecology. 2016;123:44. | Conference abstract |
|  | Torjesen I: Emergency admissions for diabetes fall by almost 7% in integrated care pilot scheme. BMJ (Clinical research ed) 2012, 344:e3562. | Editorial |
|  | Tucker H: Integrating care in Norfolk - progress of a national pilot. Journal of Integrated Care 2010, 18(1):31-37. | Describes the pilot in Norfolk, focuses on the key principles underlying the design however, there are no data apart from a few quotes from patients regarding what change is required. |
|  | Twigg MJ, Bhattacharya D, Desborough JA, Wright D: A drop-in clinic for patients with poorly-controlled diabetes: a community pharmacy feasibility study. International Journal of Clinical Pharmacy 2015, 37(2):395-402. | Reports a study to explore whether a drop in pharmacy clinic is feasible to implement. Data relate to acceptability and pharmacist views of potential benefits for patients. |
|  | Turner, Simon, Allen, Pauline, Bartlett, Will, Perotin, Virginie. Innovation and the English National Health Service: A qualitative study of the independent sector treatment centre programme. Social Science & Medicine 2011; 73 (4) 522-529 | Not new models of care in NHS |
|  | Turner, S. Ramsay, A. Perry, C. Boaden, R. et al. Lessons for major system change: centralization of stroke services in two metropolitan areas of England. Journal of Health Services & Research Policy 2016 21 (3) 156-165 | Not new models of care |
|  | Urban R, Buchan R, Turner R: Implementation of a pharmacy urgent repeat medicines (PURM) service to reduce burden on out-of-hours provision. International Journal of Pharmacy Practice 2016, 24:8-9. | Conference abstract |
|  | Vakil Z, Kong C: An audit of a multidisciplinary foot clinic effectiveness in West Hertfordshire. Diabetic Medicine 2015, 32:154-155. | Conference abstract |
|  | Ward V, Pinkney L, Fry G. Developing a framework for gathering and using service user experiences to improve  integrated health and social care: the SUFFICE  framework. BMC Res Notes (2016) 9:437 | Describes the development of survey tool. |
|  | Wilding H: Integrating care: from horizontal to vertical integration. Journal of Integrated Care 2010, 18(3):15-20. | Describes the initiatives being introduced in Torbay, no data. |
|  | Wilkinson DL, McCarthy M. Use of comparative data for integrated cancer services. BMC Health Services Research 2007; 7:204 doi:10.1186/1472-6963-7-204 | Describes use of regional and national data for comparisons |
|  | Wistow G, Callaghan G: Connected care in Hartlepool re-visited: can a holistic and community-centred approach survive implementation? Journal of Integrated Care 2008, 16(2):5-14. | Describes the process of developing the bid to become a pilot site. Provides no data apart from some quotes in the bid document. |
|  | Wodchis WP, Dixon A, Anderson GM, Goodwin N. Integrating care for older people with complex needs: key insights and lessons from a seven-country cross-case analysis. International Journal of Integrated Care 2015, 15 e012 | Discusses different models, useful overview. |
|  | Woodman J, Lewis H, Cheung R, Gilbert R, Wijlaars LP: Integrating primary and secondary care for children and young people: sharing practice. Archives of Disease in Childhood 2016, 101(9):792-797. | A realist exploration of mechanisms underpinning initiatives in 5 case studies. Describes carrying out interviews to collect data and outlines initiatives and mechanisms but provides no data to support the conclusions. |
|  | Woods L: Evaluating the clinical effectiveness of neonatal nurse practitioners: an exploratory study. Journal of Clinical Nursing 2006, 15(1):35-44. | Describes the clinical effectiveness of the role rather than service provision outcomes |
|  | Woolrych R, Sixsmith J. Integrated services for dementia: The formal carer experience. Alzheimer's and Dementia 2013, Vol 1, P528 | Conference abstract |
|  | Zakaria S, Ranmal R: Building the evidence of new models of CVD care from the integrated care pilots. Global Heart 2016, 1):e105. | Conference abstract |
|  | Zhang J, Burridge L, Baxter K, Donald M, Foster M, Hollingworth S, Ware R, Russell A, Jackson C: A new model of integrated primary-secondary care for complex diabetes in the community: study protocol for a randomised controlled trial. Trials 2013, 14:382. | Protocol only |
|  | Currie G, Finn R, Martin G. Accounting for the 'dark side' of new organizational forms: the case of healthcare professionals. Human Relations. 2008;n61(4):539e - 64. | Not specifically relating to integrated care |
|  | Doherty C. A qualitative study of health service reform on nurses' working lives: learning from the UK National Health Service (NHS). International journal of nursing studies. 2009;46(8):1134-42. | Not specifically relating to integrated care |
|  | Kümpers S, Mur I, Hardy B, van Raak A, Maarse H. Integrating dementia care in England and The Netherlands: Four comparative local case studies. Health & Place. 2006;12(4):404-20. | Focus on clinical care rather than integration of services |
|  | Procter S, Wilson PM, Brooks F, Kendall S. Success and failure in integrated models of nursing for long term conditions: Multiple case studies of whole systems. International Journal of Nursing Studies. 2013;50(5):632-43. | Focus on models of care rather than integration of services |
|  | Richards DA, Lankshear AJ, Fletcher J, Rogers A, Barkham M, Bower P, et al. Developing a U.K. protocol for collaborative care: a qualitative study. General hospital psychiatry. 2006;28(4):296-305. | Focus on collaboration in clinical care rather than integration |
|  | Rowe F, Walker M, Rockliffe J, Pollock A, Noonan C, Howard C, et al. Delivery of high quality stroke and vision care: experiences of UK services. Disability and Rehabilitation. 2016;38(8):813-7. | Focus on providing care for vision within stroke care rather than integrating services |
|  | Samuels S, Hall I, Parkes C, Hassiotis A. Professional staff and carers' views of an integrated mental health service for adults with learning disabilities. Psychiatric Bulletin. 2007;31(1):13-6. | Relates to clinical care rather than integration |
|  | Turner S, Allen P, Bartlett W, Perotin V. Innovation and the English National Health Service: A qualitative study of the independent sector treatment centre programme. Social Science & Medicine. 2011;73(4):522-9. | Evaluates independent treatment centres with no data relating to integration between these and NHS services |
|  | Turner S, Ramsay A, Perry C, Boaden R, McKevitt C, Morris S, et al. Lessons for major system change: centralization of stroke services in two metropolitan areas of England. Journal of Health Services & Research Policy. 2016;21(3):156-65. | Focus on service centralisation, with no data relating to integration |
|  | Parkes C, Samuels S, Hassiotis A, Lynggaard H, Hall I. Incorporating the views of service users in the development of an integrated psychiatric service for people with learning disabilities. British Journal of Learning Disabilities. 2007;35(1):23-9. | Describes views of service users regarding the environment of a new ward rather than relating to integration |
|  | Ellis A, Trappes-Lomax T, Fox M, Taylor R, Power M, Stead J, et al. Buying Time II: an economic evaluation of a joint NHS/Social Services residential rehabilitation unit for older people on discharge from hospital. Health & Social Care in the Community. 2006;14(2):95-106. | Not specifically integrated care |
|  | Hendry A. Lanarkshire's managed care network: an integrated improvement collaborative. Journal of Integrated Care. 2010;18(3):45-51. | Not specifically integrated care |
|  | McVean AJ, Shenkin SD, Coull AJ. Service redesign in acute medicine of the elderly wards. Age and Ageing. 2012;41:i20. | Service redesign generally rather than integrated care focus |
|  | Nazar H, Nazar Z, Simpson J, Yeung A, Whittlesea C. Use of a service evaluation and lean thinking transformation to redesign an NHS 111 refer to community Pharmacy for Emergency Repeat Medication Supply Service (PERMSS). BMJ Open. 2016;6(8):e011269. | Not specifically integrated care |
|  | Peel C, Thomas S, Worth P. Developing an integrated care pathway: the process and its application to neurological conditions. British Journal of Neuroscience Nursing. 2013;9(6):292-300. | No integration across services or professions |
|  | Rigby L, Hannah J, Haworth K, Molloy L, Scutts K. An audit of an integrated care pathway for a crisis resolution/home treatment team. British Journal of Occupational Therapy. 2007;70(12):527-33. | Limited data regarding implementation |
|  | Trappes-Lomax T, Ellis A, Fox M, Taylor R, Power M, Stead J, et al. Buying Time I: a prospective, controlled trial of a joint health/social care residential rehabilitation unit for older people on discharge from hospital. Health & Social Care in the Community. 2006;14(1):49-62. | Not specifically integrated care |
|  | Nuffield Trust. Evaluation of the first year of the Inner North West London Integrated Care Pilot. London: Nuffield Trust, 2013. | Other, more detailed evaluations of this study included |

**Review papers excluded at full paper review**

|  | Adinolfi P. Barriers to reforming healthcare: the Italian case. Health care analysis : HCA : journal of health philosophy and policy. 2014;22(1):36-58. | Descriptive overview |
| --- | --- | --- |
|  | Amiel JM, Pincus HA. The medical home model: new opportunities for psychiatric services in the United States. Current opinion in psychiatry 2011; 24 (6) 562-8 | Not new model |
|  | Anderson A, Chojnacka I. Benefits of using the Liverpool Care Pathway in end of life care. Nursing Standard. 2012;26(34):42-50. | General description |
|  | Anderson, Kathryn J.A review of health care reform in the United States and in Alaska. International journal of circumpolar health 2010 69 (5) 424-36 | Not systematic review |
|  | Avery MD, Montgomery O, Brandl-Salutz E. Essential components of successful collaborative maternity care models: the ACOG-ACNM project. Obstetrics and gynecology clinics of North America. 2012; 39 (3) 423-34 | Not systematic review |
|  | Bakker, Franka C.Robben, Sarah H. M., Olde Rikkert, Marcel G. M. Effects of hospital-wide interventions to improve care for frail older inpatients: a systematic review. BMJ quality & safety 2011; 20 (8) 680-91 | Interventions not service models |
|  | Bambra, Clare, Garthwaite, Kayleigh, Hunter, David. All things being equal: does it matter for equity how you organize and pay for health care? A review of the international evidence. International journal of health services : planning, administration, evaluation 2014; 44 (3) 457-77 | About financial equity not new service models |
|  | Battista RN, Blancquaert I, Laberge AM, van Schendel N, Leduc N. Genetics in health care: an overview of current and emerging models. Public health genomics. 2012;15(1):34-45. | Relates to clinical models rather than service delivery |
|  | Batty, C. Systematic review: interventions intended to reduce admission to hospital of older people. International Journal of Therapy & Rehabilitation. 2010; 17 (6) 310-322 | More than 50% of included papers not within scope. |
|  | Bauer, Amy M. Thielke, Stephen M. Katon, Wayne Unutzer, Jurgen Arean, Patricia. Aligning health information technologies with effective service delivery models to improve chronic disease care. Preventive medicine 2014, 66 167-72 | Not systematic review |
|  | Belgaied W, Urbinati D, Toumi M. Funding Integrated Health Care Services. Value in Health, 2014 A323-A686 | Conference Abstract |
|  | Block, Keith I. On models for integrative medical practice. Integrative cancer therapies 2007, 6 (4) 309-12 | Editorial |
|  | Boult C, Green AF, Boult LB, Pacala JT, Snyder C, Leff B. Successful models of comprehensive care for older adults with chronic conditions: evidence for the institute of medicine's 'Retooling for an aging America' report. Journal of the American Geriatrics Society. 2009;57(12):2 | Conference paper |
|  | Bourbeau, Jean, Saad, Nathalie. Integrated care model with self-management in chronic obstructive pulmonary disease: from family physicians to specialists. Chronic respiratory disease 2010 10 (2) 99-105 | Not systematic review |
|  | Bradford, Daniel W., Cunningham, Natasha T.. Slubicki, Monica N. et al. An evidence synthesis of care models to improve general medical outcomes for individuals with serious mental illness: a systematic review. The Journal of clinical psychiatry 2013; 74 (8) e754-64 | Interventions not service models |
|  | Brand C, Hunter D, Hinman R, March L, Osborne R, Bennell K. Improving care for people with osteoarthritis of the hip and knee: how has national policy for osteoarthritis been translated into service models in Australia? International journal of rheumatic diseases. 2011;14(2):181-90. | Descriptive |
|  | Brand, Caroline A., Barker, Anna L., Morello, Renata T. et al. A review of hospital characteristics associated with improved performance. International journal for quality in health care : journal of the International Society for Quality in Health Care / ISQua 2012; 24 (5) 483-94 | About hospital performance not service models |
|  | Briggs, C. J. Garner, P. Strategies for integrating primary health services in middle- and low-income countries at the point of delivery. The Cochrane database of systematic reviews 2006, Issue 2. | Low-middle income countries |
|  | Briggs, Philip D. Back to the future: the way forward in health care reform. Family practice management 2012, 19 (3) 5-6 | Not systematic review |
|  | Brink-Huis, Anita, van Achterberg, Theo, Schoonhoven, Lisette. Pain management: a review of organisation models with integrated processes for the management of pain in adult cancer patients. Journal of clinical nursing 2008; 17 (15) 1986-2000 | Interventions not service models |
|  | Bruera, Eduardo, Hui, David. Conceptual models for integrating palliative care at cancer centers. Journal of palliative medicine 2012, 15 (11) 1261-9 | Not systematic review |
|  | Burns, Lawton Robert, Muller, Ralph W. Hospital-physician collaboration: landscape of economic integration and impact on clinical integration. The Milbank quarterly 2008, 86 (3) 375-434 | Not systematic review |
|  | Butler, Mary, Kane, Robert L., McAlpine, Donna et al., Does integrated care improve treatment for depression? A systematic review. The Journal of ambulatory care management 2011; 34 (2) 113-25 | Interventions not service models |
|  | Cameron A, Lart R. Revisiting joint working. Journal of Integrated Care. 2012;20(2):89-93. | Descriptive |
|  | Carey TS, Crotty KA, Morrissey JP,et. al. Future research needs for evaluating the integration of mental health and substance abuse treatment with primary care. Journal of psychiatric practice 2013; 19 (5) 345-59 | Not systematic review |
|  | Carter, R., Riverin, B., Levesque, J. F. eta l. The impact of primary care reform on health system performance in Canada: a systematic review. BMC Health Services Research 2016; 16 p324 | Interventions not service models |
|  | Cerimele JM, Strain JJ. Integrating primary care services into psychiatric care settings: a review of the literature. Primary Care Companion to the Journal of Clinical Psychiatry. 2010;12(6). | Relates to integration of clinical care not services |
|  | Chung, Vincent C. H., Ma, Polly H. X., Hong, Lau Chun, Griffiths, Sian M. Organizational determinants of interprofessional collaboration in integrative health care: systematic review of qualitative studies. PloS one 2012; 7 (11) e50022 | Interventions not service models |
|  | Clauser, Steven B. Wagner, Edward H. Aiello Bowles, Erin J. Tuzzio, Leah Greene, Sarah M. Improving modern cancer care through information technology. American Journal of preventive medicine 2011, 40, 5 suppl 2, S198-207 | Not systematic review |
|  | Coleman, Katie, Austin, Brian T., Brach, Cindy, Wagner, Edward H. Evidence on the Chronic Care Model in the new millennium. Health affairs (Project Hope) 2009; 28 (1) 75-85 | Focus on CCM |
|  | Cooper, K. Brailsford, S. C. Davies, R. Raftery, J. A review of health care models for coronary heart disease interventions. Health care management science 2006, 9 (4) 311-24 | Not systematic review |
|  | Coulter, I. D., Khorsan, R., Crawford, C., Hsiao, A. Integrative Health Care Under Review: An Emerging Field. Journal of Manipulative & Physiological Therapeutics 2010; 33 (9) 690-710 | No data |
|  | Cresswell, Kathrin, Sheikh, Aziz. Organizational issues in the implementation and adoption of health information technology innovations: an interpretative review. International journal of medical informatics. 2013; 82 (5) e73-86 | Innovation, not service model |
|  | Damery S, Flanagan S, Combes G. The effectiveness of interventions to achieve co-ordinated multidisciplinary care and reduce hospital use for people with chronic diseases: Study protocol for a systematic review of reviews. Systematic Reviews. 2015;4 (1) (no pagination)(64). | Review protocol |
|  | Deschodt M, Claes V, Van Grootven B, Van den Heede K, Flamaing J, Boland B, et al. Structure and processes of interdisciplinary geriatric consultation teams in acute care hospitals: A scoping review. International Journal of Nursing Studies. 2016;55:98-114. | Descriptive scoping review, no data |
|  | Doebbeling BN, Flanagan ME. Emerging perspectives on transforming the healthcare system: redesign strategies and a call for needed research. Medical care. 2011;49 Suppl:S59-64. | Descriptive overview, not a systematic review |
|  | Dudley L, Garner P. Strategies for integrating primary health services in low- and middle-income countries at the point of delivery. The Cochrane database of systematic reviews. 2011(7):CD003318. | Focus on LMICs |
|  | Ehrlich C, Kendall E, Muenchberger H, Armstrong K. Coordinated care: what does that really mean? Health & social care in the community. 2009;17(6):619-27. | Commentary, not a systematic review |
|  | Eldridge GN, Korda H. Value-based purchasing: the evidence. The American journal of managed care. 2011;17(8):e310-3. | Not a systematic review or models of care |
|  | Evans JM, Baker GR, Berta W, Barnsley J. The evolution of integrated health care strategies. Advances in health care management. 2013;15:125-61. | Conceptual paper, not a systematic review |
|  | Evans JM, Baker GR. Shared mental models of integrated care: aligning multiple stakeholder perspectives. Journal of health organization and management. 2012;26(6):713-36. | Conceptual paper, not a systematic review |
|  | Farooq S. Collaborative care for depression: a literature review and a model for implementation in developing countries. International health. 2013;5(1):24-8. | Not a systematic review, focus on LMICs |
|  | Filson CP, Hollingsworth JM, Skolarus TA, Clemens JQ, Hollenbeck BK. Health care reform in 2010: transforming the delivery system to improve quality of care. World journal of urology. 2011;29(1):85-90. | Descriptive overview, not a systematic review |
|  | Fisher L, Dickinson WP. Psychology and primary care: New collaborations for providing effective care for adults with chronic health conditions. The American psychologist. 2014;69(4):355-63. | Descriptive overview, not a systematic review |
|  | Fisher MP, Elnitsky C. Health and social services integration: a review of concepts and models. Social work in public health. 2012;27(5):441-68. | Conceptual paper, not a systematic review |
|  | Fitzsimons M, Normand C, Varley J, Delanty N. Evidence-based models of care for people with epilepsy. Epilepsy & behavior : E&B. 2012;23(1):1-6. | Narrative review |
|  | Flannery F, Adams D, O'Connor N. A community mental health service delivery model: integrating the evidence base within existing clinical models. Australasian psychiatry : bulletin of Royal Australian and New Zealand College of Psychiatrists. 2011;19(1):49-55. | Conceptual paper, not a systematic review |
|  | Ford SR, Pearse RM. Do integrated care pathways have a place in critical care? Current opinion in critical care. 2012;18(6):683-7. | Narrative review |
|  | Franx G, Dixon L, Wensing M, Pincus H. Implementation strategies for collaborative primary care-mental health models. Current opinion in psychiatry. 2013;26(5):502-10. | Review of implementation strategies rather than care models *per se* |
|  | Franx G, Kroon H, Grimshaw J, Drake R, Grol R, Wensing M. Organizational change to transfer knowledge and improve quality and outcomes of care for patients with severe mental illness: a systematic overview of reviews. Canadian journal of psychiatry Revue canadienne de psychiatrie. 2008;53(5):294-305. | Review of reviews, most too old for inclusion |
|  | French RS, Coope CM, Graham A, Gerressu M, Salisbury C, Stephenson JM. One stop shop versus collaborative integration: what is the best way of delivering sexual health services? Sexually Transmitted Infections. 2006;82(3):202-6. | Not systematic review (narrative review plus qualitative interviews) |
|  | Fry MM. Barriers and facilitators for successful after hours care model implementation: reducing ED utilisation. Australasian Emergency Nursing Journal. 2009;12(4):137-44. | Narrative review of barriers/facilitators |
|  | Gagliardi AR, Dobrow MJ, Wright FC. How can we improve cancer care? A review of interprofessional collaboration models and their use in clinical management. Surgical oncology. 2011;20(3):146-54. | Narrative/descriptive overview |
|  | Gesundheit Österreich GmbH. Integrated mental health care of children and adolescents (Structured abstract). Health Technology Assessment Database. 2015(3). | Report in German |
|  | Gilbody S, Bower P, Fletcher J, Richards D, AJ. S. Collaborative care for depression: a cumulative meta-analysis and review of longer-term outcomes. Arch Intern Med 2006;166(21):2314–21. | Only reports individual clinical outcomes |
|  | Giorda CB. The role of the care model in modifying prognosis in diabetes. Nutrition, metabolism, and cardiovascular diseases : NMCD. 2013;23(1):11-6. | Narrative review not systematic review |
|  | Glenton C, Colvin CJ, Carlsen B, Swartz A, Lewin S, Noyes J, et al. Barriers and facilitators to the implementation of lay health worker programmes to improve access to maternal and child health: qualitative evidence synthesis. Cochrane Database of Systematic Reviews. 2013(10). | Lay health workers not integrated service |
|  | Goodman C, Dening T, Gordon AL, Davies SL, Meyer J, Martin FC, et al. Effective health care for older people living and dying in care homes: a realist review. BMC Health Services Research. 2016;16:269. | UK-focused review |
|  | Grover A, Niecko-Najjum LM. Primary care teams: are we there yet? Implications for workforce planning. Academic medicine : journal of the Association of American Medical Colleges. 2013;88(12):1827-9. | Commentary |
|  | Ham C. The ten characteristics of the high-performing chronic care system. Health Economics, Policy and Law. 2010;5(1):71-90. | Narrative review/commentary, not systematic review |
|  | Harfield S, Davy C, Kite E, McArthur A, Munn Z, Brown N, et al. Characteristics of Indigenous primary health care models of service delivery: a scoping review protocol. JBI Database Of Systematic Reviews And Implementation Reports. 2015;13(11):43-51. | Protocol for systematic review |
|  | Helfgott AW. The patient-centered medical home and accountable care organizations: an overview. Current opinion in obstetrics & gynecology. 2012;24(6):458-64. | Narrative overview |
|  | Hoeper K, Amelung VE, Hartmann J, Hermanowski T, Krauth C. Integrated care programs in europe: Factors of success. Value in Health. 2013;16 (3):A197-A8 | Conference abstract |
|  | Horsfall J, Cleary M, Hunt GE. Acute inpatient units in a comprehensive (integrated) mental health system: a review of the literature. Issues in mental health nursing. 2010;31(4):273-8. | Narrative review |
|  | Howarth M, Holland K, Grant MJ. Education needs for integrated care: a literature review. Journal of advanced nursing. 2006;56(2):144-56. | Not international review (UK focus) |
|  | Huffman JC, Niazi SK, Rundell JR, Sharpe M, Katon WJ. Essential articles on collaborative care models for the treatment of psychiatric disorders in medical settings: a publication by the academy of psychosomatic medicine research and evidence-based practice committee. Psychosomatics. 2014;55(2):109-22. | Descriptive bibliography, no synthesis |
|  | Hunter B, Segrott J. Are clinical pathways 'a good thing'? Reviewing the evidence. MIDIRS Midwifery Digest. 2009;19(4):515-7. | Summary of a review |
|  | Hurlow AB, Godfrey M, Bennett MI. A conceptual framework for integrated palliative care interventions: Understanding when pathways work, don't work and why. Palliative Medicine. 2012;26 (4):531. | Conference abstract |
|  | Jansen DEMC, Krol B, Groothoff JW, Post D. Integrated care for MS patients. Disability and rehabilitation. 2007;29(7):597-603. | Commentary |
|  | Jenkinson J, Howard R. Provision of specialist continuing care services for older adults across the UK. International Psychogeriatrics. 2016;28(6):959-66. | Narrative review and UK survey |
|  | Johnson C. Health care transitions: a review of integrated, integrative, and integration concepts. Journal of manipulative and physiological therapeutics. 2009;32(9):703-13. | Editorial |
|  | Jortberg BT, Miller BF, Gabbay RA, Sparling K, Dickinson WP. Patient-centered medical home: how it affects psychosocial outcomes for diabetes. Current diabetes reports. 2012;12(6):721-8. | Narrative review |
|  | Kash BA, Zhang Y, Cline KM, Menser T, Miller TR: The perioperative surgical home (PSH): a comprehensive review of US and non-US studies shows predominantly positive quality and cost outcomes. The Milbank quarterly 2014, 92(4):796-821. | Data predominantly relate to patient education pre-operatively and surgical processes rather than care integration |
|  | Katon W, Unützer J. Collaborative Care Models for DepressionTime to Move From Evidence to Practice. Arch Intern Med. 2006;166(21):2304-2306. doi:10.1001/archinte.166.21.2304 | Editorial |
|  | Kim JJ, Kim CM: Models for joint ophthalmology-optometry patient management. Current opinion in ophthalmology 2011, 22(4):256-260. | Descriptive overview |
|  | Kim K, Choi JS, Choi E, Nieman CL, Joo JH, Lin FR, Gitlin LN, Han HR: Effects of Community-Based Health Worker Interventions to Improve Chronic Disease Management and Care Among Vulnerable Populations: A Systematic Review. American Journal of Public Health 2016, 106(4):E3-E28. | Evaluates the role of established community workers |
|  | Kitsiou S, Pare G, Jaana M: Systematic Reviews and Meta-Analyses of Home Telemonitoring Interventions for Patients With Chronic Diseases: A Critical Assessment of Their Methodological Quality. Journal of Medical Internet Research 2013, 15(7):216-238. | Relates to use of technology and care outcomes |
|  | Kodner DL: Whole-system approaches to health and social care partnerships for the frail elderly: an exploration of North American models and lessons. Health & social care in the community 2006, 14(5):384-390. | Descriptive overview of Program of All-Inclusive Care for Elderly People (PACE) programme in the United States, and the Système de soins Intégrés pour Personnes Âgées (SIPA) and the  Programme of Research to Integrate Services for the Maintenance of  Autonomy (PRISMA) in Canada. |
|  | Koea JB, Srinivasa S, Hundal H: Provision of acute general surgery: a systematic review of models of care. The journal of trauma and acute care surgery 2014, 76(1):219-225. | Review of models of surgical care rather than integrated working or models |
|  | Kuhlmann AS, Gavin L, Galavotti C: The Integration of Family Planning with Other Health Services: A Literature Review. International Perspectives on Sexual and Reproductive Health 2010, 36(4):189-196. | Primary studies from developing countries |
|  | Lee SJ, Crowther E, Keating C, Kulkarni J: What is needed to deliver collaborative care to address comorbidity more effectively for adults with a severe mental illness? The Australian and New Zealand journal of psychiatry 2013, 47(4):333-346. | Describes searching databases but is not a systematic review |
|  | Lee S-YD, Weiner BJ, Harrison MI, Belden CM: Organizational transformation: a systematic review of empirical research in health care and other industries. Medical care research and review : MCRR 2013, 70(2):115-142. | Only 13 of 56 included studies were in healthcare |
|  | Loader BD, Hardey M, Keeble L: Health informatics for older people: A review of ICT facilitated integrated care for older people. International Journal of Social Welfare 2008, 17(1):46-53. | Descriptive overview of literature |
|  | MacAdam M. Frameworks of Integrated Care for the Elderly: A Systematic Review. Ontario: Canadian Policy Research Networks, 2008 | Described as a systematic review but processes for selection of studies unclear and does not meet criteria for a systematic review. |
|  | MacLure K, Stewart D, Strath A: Mind the gap: Multidisciplinary team perceptions of ehealth in relation to integrated care. International Journal of Pharmacy Practice 2014, 22:16. | Conference presentation |
|  | Makoul G, Clayman ML: An integrative model of shared decision making in medical encounters. Patient Education and Counseling 2006, 60(3):301-312. | Conference presentation |
|  | Mannion R: General practitioner commissioning in the English National Health Service: continuity, change, and future challenges. International journal of health services : planning, administration, evaluation 2008, 38(4):717-730. | Descriptive overview |
|  | McColl MA, Shortt S, Godwin M, Smith K, Rowe K, O'Brien P, Donnelly C: Models for integrating rehabilitation and primary care: a scoping study. Archives of Physical Medicine & Rehabilitation 2009, 90(9):1523-1531. | A scoping review which describes different models of care rather than an evaluation |
|  | McDonald PS, Whittle CL, Dunn L, De Luc K: Shortfalls in integrated care pathways. Part 1: What don't they contain? Journal of Integrated Care Pathways 2006, 10(1):17-22. | Overview |
|  | McDonald PS, Whittle CL, Dunn L, De Luc K: Shortfalls in integrated care pathways. Part 2: How well are we doing? Journal of Integrated Care Pathways 2006, 10(1):23-27. | Overview |
|  | Mikocka-Walus AA, Andrews JM, von Kanel R, Moser G: What are the implications of changing treatment delivery models for patients with inflammatory bowel disease: a discussion paper. European journal of gastroenterology & hepatology 2013, 25(4):393-398. | Descriptive overview |
|  | Miller BF, Mendenhall TJ, Malik AD: Integrated primary care: an inclusive three-world view through process metrics and empirical discrimination. Journal of clinical psychology in medical settings 2009, 16(1):21-30. | Describes a methodology |
|  | Morrison RS: Models of palliative care delivery in the United States. Current opinion in supportive and palliative care 2013, 7(2):201-206. | Descriptive overview |
|  | Motheral BR: Telephone-based disease management: why it does not save money. The American journal of managed care 2011, 17(1):e10-16. | Relates to health models rather than models of care |
|  | Nuno R, Coleman K, Bengoa R, Sauto R. Integrated care for chronic conditions: the contribution of the ICCC Framework. Health policy (Amsterdam, Netherlands). 2012;105(1):55-64. | Overview of studies |
|  | O'Donnell S, Li LC, King J, Lauzon C, Finn H, Vliet Vlieland TPM: Development of a framework for reporting health service models for managing rheumatoid arthritis. Clinical rheumatology 2010, 29(2):151-165. | Relates to models of health rather than service delivery |
|  | Ouwens M, Hulscher M, Hermens R, Faber M, Marres H, Wollersheim H, Grol R: Implementation of integrated care for patients with cancer: a systematic review of interventions and effects. International journal for quality in health care : journal of the International Society for Quality in Health Care / ISQua 2009, 21(2):137-144. | Focus on patient-centred care for example information leaflet provision, only one included study of 33 refers to multi-disciplinary care |
|  | Pearson M, Hunt H, Cooper C, Shepperd S, Pawson R, Anderson R: Providing effective and preferred care closer to home: a realist review of intermediate care. Health & Social Care in the Community 2015, 23(6):577-593. | Refers to integrated care but focus is on how integrated care relates to and underpins the establishment of intermediate care. Main conclusions relate to service user understanding of intermediate care. |
|  | Pensuk P. The effectiveness of the liverpool care pathway in end of life care: A systematic review protocol. JBI Library of Systematic Reviews. 2013;11(11):64-76. | Protocol for a review |
|  | Perla RJ, Bradbury E, Gunther-Murphy C. Large-scale improvement initiatives in healthcare: a scan of the literature. Journal for healthcare quality : official publication of the National Association for Healthcare Quality. 2013;35(1):30-40. | Descriptive overview |
|  | Perla RJ, Bradbury E, Gunther-Murphy C: Large-scale improvement initiatives in healthcare: a scan of the literature. Journal for healthcare quality : official publication of the National Association for Healthcare Quality 2013, 35(1):30-40. | Used a Delphi approach to identify literature |
|  | Philp I: The contribution of geriatric medicine to integrated care for older people. Age and Ageing 2015, 44(1):11-15. | Descriptive overview of the role of geriatricians |
|  | Ramsay A, Fulop N. The Evidence Base for Integrated Care. Summary of research evidence prepared for the Department of Health, 2008. | Summary overview |
|  | Reed J, Childs S, Cook G, Hall A, McCormack B: Integrated care for older people: methodological issues in conducting a systematic literature review. Worldviews on evidence-based nursing / Sigma Theta Tau International, Honor Society of Nursing 2007, 4(2):78-85. | Describes the challenges of the methodology rather than review findings |
|  | Retchin SM: A conceptual framework for interprofessional and co-managed care. Academic medicine : journal of the Association of American Medical Colleges 2008, 83(10):929-933. | Descriptive overview |
|  | Reynolds HW, Sutherland EG: A systematic approach to the planning, implementation, monitoring, and evaluation of integrated health services. BMC health services research 2013, 13:168. | Describes how monitoring and evaluation methods may be used |
|  | Richards DA: Stepped care: a method to deliver increased access to psychological therapies. Canadian journal of psychiatry Revue canadienne de psychiatrie 2012, 57(4):210-215. | Descriptive overview of the approach |
|  | Rummery K: Healthy partnerships, healthy citizens? An international review of partnerships in health and social care and patient/user outcomes. Soc Sci Med 2009, 69(12):1797-1804. | Descriptive overview |
|  | Schmied V, Mills A, Kruske S, Kemp L, Fowler C, Homer C: The nature and impact of collaboration and integrated service delivery for pregnant women, children and families. Journal of clinical nursing 2010, 19(23-24):3516-3526. | Descriptive overview |
|  | Schottle D, Karow A, Schimmelmann BG, Lambert M: Integrated care in patients with schizophrenia: results of trials published between 2011 and 2013 focusing on effectiveness and efficiency. Current opinion in psychiatry 2013, 26(4):384-408. | Paper has a clinical rather than service delivery focus |
|  | Shaw S, Rosen R, Rumbold B. What is Integrated Care? London: Nuffield Trust, 2011. | Overview of concepts and terminology |
|  | Shortell MS, Gillies R, F. W. United States Innovations in Health Care Delivery. Public Health Reviews 2010;32:190–212. | Not a systematic review |
|  | Smith E, Ross FM: Service user involvement and integrated care pathways. International journal of health care quality assurance 2007, 20(2-3):195-214. | Focus of the paper is on how service users have been involved in different areas of health services |
|  | Smith G, Clarke D: Assessing the effectiveness of integrated interventions: terminology and approach. The Medical clinics of North America 2006, 90(4):533-548. | Discussion paper |
|  | Stokes J, Checkland K, Kristensen SR: Integrated care: theory to practice. Journal of Health Services & Research Policy 2016, 29:29. | Descriptive overview |
|  | Thaldorf C, Liberman A. Integration of health care organizations: using the power strategies of horizontal and vertical integration in public and private health systems. The health care manager. 2007;26(2):116-27. | Relates to clinical care only |
|  | Torrey WC, Tepper M, Greenwold J. Implementing Integrated Services for Adults With Co-occurring Substance Use Disorders and Psychiatric Illnesses: A Research Review. Journal of Dual Diagnosis. 2011;7(3):150-61. | Descriptive overview |
|  | Tsiachristas A, Dikkers C, Boland MRS, Rutten-van Molken MPMH: Exploring payment schemes used to promote integrated chronic care in Europe. Health policy 2013, 113(3):296-304. | Described as a review and interview study. The review is descriptive and no data from the interviews is presented. |
|  | Wilson P, Bunn F, Morgan J: A mapping of the evidence on integrated long term condition services. British journal of community nursing 2009, 14(5):202-206. | Provides an overview (map) of literature rather than a systematic review |
|  | Wiltsey Stirman S, Kimberly J, Cook N, Calloway A, Castro F, Charns M: The sustainability of new programs and innovations: a review of the empirical literature and recommendations for future research. Implementation science : IS 2012, 7:17. |  |
|  | Wistow G: Integration and the NHS reforms. Journal of Integrated Care 2011, 19(4):5-13. | Focus on all literature reporting sustainability outcomes |
|  | Wulsin LR, Sollner W, Pincus HA: Models of integrated care. The Medical clinics of North America 2006, 90(4):647-677. | Descriptive overview |

**Non-UK studies excluded at full paper review**

|  | Reference | Reason for exclusion |
| --- | --- | --- |
|  | Abdul, A. A.Muhammad, N. A. Sulong, S.  Aljunid, S. The integrated care pathway for managing post stroke (iCaPPS) patients in the community: A cost-effectiveness analysis. Value in health 2014 17 (7) A761 | Conference abstract |
|  | Auerbach, David I. Liu, Hangsheng, Hussey, Peter S.  Lau, Christopher, Mehrotra, Ateev. Accountable care organization formation is associated with integrated systems but not high medical spending. Health Affairs 2013 32 (10) 1781-8 | No intervention |
|  | Bayliss, Elizabeth A. Bhardwaja, Bharati, Ross, Colleen. Beck, Arne, Lanese, Diane M. Multidisciplinary team care may slow the rate of decline in renal function. Clinical journal of the American Society of Nephrology : CJASN 2011 6(4) 704-10 | Clinical outcomes |
|  | Bergmo, T., Berntsen, G. Dalbakk, M. Rumpsfeld, M. The effectiveness and cost effectiveness of the PAtient-Centred Team (PACT) model: study protocol of a prospective matched control before-and-after study. BMC geriatrics 2015 15; 133 | Study protocol |
|  | Bissonnette, J. Woodend, K. Davies, B. Stacey, D.  Knoll, G. A. Evaluation of a collaborative chronic care approach to improve outcomes in kidney transplant recipients. Clinical transplantation 2013 27 (2) 232-8 | Clinical outcomes |
|  | Bleijenberg, Nienke, Drubbel, Irene, Ten Dam, Valerie H. Numans, Mattijs E. Schuurmans, Marieke J.de Wit, Niek J. Proactive and integrated primary care for frail older people: design and methodological challenges of the Utrecht primary care PROactive frailty intervention trial (U-PROFIT). BMC geriatrics 2012 12 p16 | Study protocol |
|  | Blom, J. Elzen, W. Houwelingen, A. H. V. Heijmans, M. Stijnen, T. Hout, W. Gussekloo, J. Effectiveness and cost-effectiveness of a proactive, goal-oriented, integrated care model in general practice for older people. A cluster randomised controlled trial: Integrated systematic care for older people-the ISCOPE study. Age and ageing 2016 45 (1) 30-41 | Health outcomes |
|  | Boland, M. Kruis, A. Tsiachristas, A. Assendelft, W.  Gussekloo, J. Blom, C. Chavannes, N. Rutten-Van, M. M. Cost-effectiveness of integrated COPD care: The RECODE cluster randomised trial. BMJ Open 2015 5 (pages not given) | Health outcomes |
|  | Boumans, Nicolle P. G. Berkhout, Afke J. M. B. Vijgen, Sylvia M. C. Nijhuis, Frans J. N. Vasse, Rineke M. The effects of integrated care on quality of work in nursing homes: a quasi-experiment. International journal of nursing studies 2008 45 (8) 1122-36 | Health worker outcomes |
|  | Brannstrom, Margareta, Boman, Kurt. A new model for integrated heart failure and palliative advanced homecare--rationale and design of a prospective randomized study. European journal of cardiovascular nursing. 2012 12 (3) 269-75 | Study protocol |
|  | Casas A, Troosters T, Garcia-Aymerich J, Roca J, Hernandez C, Alonso A, et al. Integrated care prevents hospitalisations for exacerbations in COPD patients. The European respiratory journal. 2006;28(1):123-30. | Focus not on integrated care |
|  | Chan, J. Jia, W. Li, W. Guo, X. Zhang, Y. Li, X. et al. The effects of a web-based integrated care program with or with out a nurse coordinator on cardiometabolic control in type 2 diabetes-the china jade (joint asia diabetes evaluation) program. Diabetes 2014 63 A301 | Clinical outcomes |
|  | Chaney, Edmund F. Rubenstein, Lisa V. Liu, Chuan-Fen, Yano, Elizabeth M. Bolkan, Cory et al. Implementing collaborative care for depression treatment in primary care: a cluster randomized evaluation of a quality improvement practice redesign. Implementation science : IS 2011 6 121 | Health worker outcomes |
|  | Coburn, K. Marcantonio, S. Lazansky, R. Keller, M.  Davis, N. Effect of a community-based nursing intervention on mortality in chronically ill older adults: a randomized controlled trial. PLoS medicine 2012 9 (7) e1001265 | Clinical outcomes |
|  | Conrad, Douglas, Fishman, Paul, Grembowski, David  Ralston, James, Reid, Robert et al. Access intervention in an integrated, prepaid group practice: effects on primary care physician productivity. Health services research2008 43 5 Pt 21 888-905 | Health worker outcomes |
|  | Costa, F. Porcu, A. Balestracci, S. Mignani, D.  Magnani, F. Cost and effectiveness of 2 years integrated care intervention in COPD. European Respiratory Journal 2015 46 (no page numbers) | Conference abstract |
|  | Doubova, S. V. Infante-Castaneda, C. Espinosa-Alarcon, P. Flores-Hernandez, S. Martinez-Vega, I.  Perez-Cuevas, R. Effectiveness of an integrative health-care model for climacteric-stage women. Climacteric 2013 16 (5) 590-600 | Health outcomes |
|  | Dubuc, Nicole, Dubois, Marie-France, Raiche, Michel  Gueye, N'Deye Rokhaya, Hebert, Rejean. Meeting the home-care needs of disabled older persons living in the community: does integrated services delivery make a difference? BMC geriatrics 2011 11 p67 | Patient need outcomes |
|  | Erickson, Steven, Hambleton, Jeffrey. A pharmacy's journey toward the patient-centered medical home. Journal of the American Pharmacists Association 2011 51(2) 156-60 | No data |
|  | Esselens, Greet, Westhovens, Rene, Verschueren, Patrick. Effectiveness of an integrated outpatient care programme compared with present-day standard care in early rheumatoid arthritis. Musculoskeletal care 2009 7 (1) 1-16 | Health outcomes |
|  | Fairbrother, G. Jones, A. Rivas, K. Changing model of nursing care from individual patient allocation to team nursing in the acute inpatient environment. Contemporary Nurse 2010 35 (2) 202-220 | Health worker outcomes |
|  | Fifield, Judith, Forrest, Deborah Dauser, Martin-Peele, Melanie, Burleson, Joseph A. Goyzueta, Jeanette, Fujimoto, Marco, Gillespie, William. A randomized, controlled trial of implementing the patient-centered medical home model in solo and small practices. Journal of general internal medicine. 2013 28 (6) 770-7 | Quality assurance outcomes |
|  | Garcia-Aymerich J, Hernandez C, Alonso A, Casas A, Rodriguez-Roisin R, Anto JM, et al. Effects of an integrated care intervention on risk factors of COPD readmission. Respiratory medicine. 2007;101(7):1462-9. | Focus not on integrated care |
|  | Ho, S. Groessl, E. Brau, N. Cheung, R. Weingart, K.  Ward, M. Prospective multisite randomized trial of Integrated Care (IC) vs. Usual Care (UC) for improving access to antiviral therapy for high risk patients with chronic HCV. Journal of hepatology 2012 56 S386 | Conference abstract |
|  | Kanter, Michael H. Lindsay, Gail, Bellows, Jim, Chase, Alide. Complete care at Kaiser Permanente: transforming chronic and preventive care. Joint Commission journal on quality and patient safety / Joint Commission Resources 2013 39 (11) 484-94 | Health outcomes |
|  | Katon W, Unutzer J, Fan MY, Williams JW, Jr., Schoenbaum M, Lin EH, et al. Cost-effectiveness and net benefit of enhanced treatment of depression for older adults with diabetes and depression. Diabetes Care. 2006;29(2):265-70. | Intervention enhanced care rather than integration of services |
|  | Kruis, Annemarije L. Boland, Melinde R. S. Assendelft, Willem J. J. Gussekloo, Jacobijn, Tsiachristas, Apostolos. et al. Effectiveness of integrated disease management for primary care chronic obstructive pulmonary disease patients: results of cluster randomised trial. BMJ 2014 349 g5392 | Health outcomes |
|  | Lambeek, L. Bosmans, J. Royen, B. Tuler, M. Mechelen, W. Anema, J. Effect of integrated care for sick listed patients with chronic low back pain: economic evaluation alongside a randomised controlled trial. BMJ 2010 341:c6414 2 | Health worker outcomes |
|  | Leeuwen, K. Bosmans, J. Jansen, A. Hoogendijk, E.  Muntinga, M. et al. Cost-Effectiveness of a Chronic Care Model for Frail Older Adults in Primary Care: Economic Evaluation Alongside a Stepped-Wedge Cluster-Randomized Trial. Journal of the American Geriatrics Society 2015 63 (12) 2494-2504 | Clinical outcomes |
|  | Legido, P. Lanzeta, I. Begiristain, M. Iza, I. Rodriguez, J. et al. Results of the introduction of a comprehensive health care model for plurypathological patients. European journal of internal medicine 2013 24 e231 | Conference abstract |
|  | Makai, P. Looman, W. Adang, E. Melis, R. Stolk, E.  Fabbricotti, I. Cost-effectiveness of integrated care in frail elderly using the ICECAP-O and EQ-5D: does choice of instrument matter? European Journal of Health Economics 2014 2 epub | Health outcomes |
|  | Marcu, Mircea I. Knapp, Caprice A. Madden, Vanessa L. Wang, Hua Kaufmann, Meggen Sloyer, Phyllis. Effect of an integrated care system on utilization for CSHCN in Florida. Maternal and child health journal 2014 18 (1) 38-44 | Medicaid utilisation outcomes |

Non-UK non-comparator studies excluded at full paper review

|  | Andersson A-C. Managers' views and experiences of a large-scale county council improvement program: limitations and opportunities. Quality management in health care. 2013;22(2):152-60. | Quality improvement rather than integration |
| --- | --- | --- |
|  | Baloh J, Zhu X, Vaughn T, MacKinney AC, Mueller KJ, Ullrich F, et al. Facilitating the formation of accountable care organizations in rural areas. Rural policy brief. 2014(2014 9):1-4. | Policy brief, no data |
|  | Barraclough F, Longman J, Barclay L. Integration in a nurse practitioner-led mental health service in rural Australia. Australian Journal of Rural Health. 2016;24(2):144-50. | Description of service |
|  | Block R, Slomp M, Patterson S, Jacobs P, Ohinmaa AE, Yim R, Dewa CS: The impact of integrating mental and general health services on mental health's share of total health care spending in Alberta. Psychiatric services (Washington, DC) 2008, 59(8):860-863. | Focus on comparing share of spend between mental health and general services |
|  | Bray P, Cummings DM, Thompson DK. Use of integrated care delivery to improve the quality of diabetes management among African Americans. North Carolina medical journal. 2011;72(5):390-2. | Relates to a reimbursement system |
|  | Callahan, Christopher M, Boustani, Malaz A, Weiner, Michael, Beck, Robin A. Implementing dementia care models in primary care settings: The Aging Brain Care Medical Home. Aging & mental health 2011 15 (1) 5-12 | Descriptive |
|  | Carlfjord S, Lindberg M, Bendtsen P, Nilsen P, Andersson A. Key factors influencing adoption of an innovation in primary health care: a qualitative study based on implementation theory. BMC family practice. 2010;11:60. | Not integration intervention |
|  | Ciccone MM, Aquilino A, Cortese F, Scicchitano P, Sassara M, Mola E, et al. Feasibility and effectiveness of a disease and care management model in the primary health care system for patients with heart failure and diabetes (Project Leonardo). Vascular health and risk management. 2010;6:297-305. | Description only |
|  | Cohen E, Lacombe-Duncan A, Spalding K, MacInnis J, Nicholas D, Narayanan U, et al. Integrated complex care coordination for children with medical complexity: a mixed-methods evaluation of tertiary care-community collaboration (Provisional abstract). BMC Health Services Research. 2012;12(2):366. | Description only |
|  | Colyer H. Accountability in integrated working: Meaning and implications for cancer care teams. Radiography. 2012;18(1):43-6. | Description only |
|  | Cremieux P-Y, Fortin P, Meilleur M-C, Montague T, Royer J. The economic impact of a partnership-measurement model of disease management: Improving Cardiovascular Outcomes in Nova Scotia (ICONS). Healthcare quarterly (Toronto, Ont). 2007;10(2):38-46. | Description only |
|  | Dahrouge S, Hogg W, Ward N, Tuna M, Devlin RA, Kristjansson E, et al. Delivery of primary health care to persons who are socio-economically disadvantaged: does the organizational delivery model matter? BMC health services research. 2013 ;13:517. | Explores the association between social disadvantage and payment systems |
|  | Dunt D, Elsworth G, Southern D, Harris C, Potiriadis M, Young D. Individual and area factors associated with general practitioner integration in Australia: a multilevel analysis. Social science & medicine (1982). 2006;63(3):680-90. | Describes a measure of GP integraion |
|  | Elg M, Stenberg J, Kammerlind P, Tullberg S, Olsson J. Swedish healthcare management practices and quality improvement work: development trends. International journal of health care quality assurance. 2011;24(2):101-23. | Reports trends in management practices |
|  | Evans JM, Baker GR, Berta W, Barnsley J. A cognitive perspective on health systems integration: results of a Canadian Delphi study. BMC health services research. 2014;14:222. | Describes development of a framework |
|  | Funderburk JS, Sugarman DE, Maisto SA, Ouimette P, Schohn M, Lantinga L, et al. The description and evaluation of the implementation of an integrated healthcare model. Families, systems & health : the journal of collaborative family healthcare. 2010;28(2):146-60. | Behavioural health providers only |
|  | Gaboury I, Boon H, Verhoef M, Bujold M, Lapierre LM, Moher D. Practitioners' validation of framework of team-oriented practice models in integrative health care: a mixed methods study. BMC health services research. 2010;10:289. | Focus on integration of alternative medicine into mainstream |
|  | Henry JKFF, Commonwealth F. Experiences and Attitudes of Primary Care Providers Under the First Year of ACA Coverage Expansion: Findings from the Kaiser Family Foundation/Commonwealth Fund 2015 National Survey of Primary Care Providers. Issue brief (Commonwealth Fund). 2015;17:1-21. | Reports survey of views on legislation |
|  | Hepworth J, Marley JE. Healthcare teams - a practical framework for integration. Australian family physician. 2010;39(12):969-71. | Development of a framework of team functioning |
|  | Jensen TB. Design principles for achieving integrated healthcare information systems. Health informatics journal. 2013;19(1):29-45. | Description only |
|  | Kirchner J, Edlund CN, Henderson K, Daily L, Parker LE, Fortney JC. Using a multi-level approach to implement a primary care mental health (PCMH) program. Families, systems & health : the journal of collaborative family healthcare. 2010;28(2):161-74. | Description only |
|  | Lamothe L, Fortin J-P, Labbe F, Gagnon M-P, Messikh D. Impacts of telehomecare on patients, providers, and organizations. Telemedicine journal and e-health : the official journal of the American Telemedicine Association. 2006;12(3):363-9. | Implementation of telemedicine |
|  | Landis SE, Barrett M, Galvin SL. Effects of different models of integrated collaborative care in a family medicine residency program. Families, systems & health : the journal of collaborative family healthcare. 2013;31(3):264-73. | Focus on type of service |
|  | Latour CHM, Huyse FJ, de Vos R, Stalman WAB. A method to provide integrated care for complex medically ill patients: the INTERMED. Nursing & health sciences. 2007;9(2):150-7. | Description only of a tool for assessing health risk |
|  | Leonard M, McGlone S, Boardman A. Taking integrated care management to the street: can we find the road to our triple aim? The Journal of ambulatory care management. 2011;34(2):192-202. | Description only |
|  | Lettieri E, Bartoli L, Masella C. Coordinating intra-sector services in healthcare: Requirements and elements that managers should take into account. European Management Journal. 2013;31(6):591-601. | General health care managemetn |
|  | Lewis VA, McClurg AB, Smith J, Fisher ES, Bynum JPW. Attributing patients to accountable care organizations: performance year approach aligns stakeholders' interests. Health affairs (Project Hope). 2013;32(3):587-95. | Methods to assign patients to ACOs |
|  | MacKay C, Veinot P, Badley EM. Characteristics of evolving models of care for arthritis: a key informant study. BMC health services research. 2008;8:147. | Describes a range of methods |
|  | Mak VSL, Clark A, Poulsen JH, Udengaard KU, Gilbert AL. Pharmacists' awareness of Australia's health care reforms and their beliefs and attitudes about their current and future roles. The International journal of pharmacy practice. 2012;20(1):33-40. | Explores views of health reform in general |
|  | Masso M, Owen A. Linkage, coordination and integration: Evidence from rural palliative care. Australian Journal of Rural Health. 2009;17(5):263-7. | Describes existing intervention |
|  | Masso M, Robert G, McCarthy G, Eagar K. The Clinical Services Redesign Program in New South Wales: perceptions of senior health managers. Australian Health Review. 2010;34(3):352-9. | Service redesign which is not integration |
|  | May SG, Cheng PH, Tietbohl CK, Trujillo L, Reilly K, Frosch DL, et al. Shared medical appointments to screen for geriatric syndromes: preliminary data from a quality improvement initiative. Journal of the American Geriatrics Society. 2014;62(12):2415-9. | Relates to a screening programme |
|  | McGeehan SK, Applebaum R. The evolving role of care management in integrated models of care. Care management journals : Journal of case management ; The journal of long term home health care. 2007;8(2):64-70. | Discusses the role of care manager and care management in general |
|  | Minkman M, Ahaus K, Fabbricotti I, Nabitz U, Huijsman R. A quality management model for integrated care: results of a Delphi and Concept Mapping study. International journal for quality in health care : journal of the International Society for Quality in Health Care / ISQua. 2009;21(1):66-75. | Development of a model |
|  | Minkman MMN, Ahaus KTB, Huijsman R. A four phase development model for integrated care services in the Netherlands. BMC health services research. 2009;9:42. | Describes stages in a process for developing care management |
|  | Minkman MMN, Vermeulen RP, Ahaus KTB, Huijsman R. The implementation of integrated care: the empirical validation of the Development Model for Integrated care. BMC health services research. 2011;11:177. | Evaluates model |
|  | Muhlbacher AC, Bethge S, Eble S. Characteristics of integrated care programmes and their impact on patient benefit: a discrete-choice experiment for integrated care networks. Gesundheitswesen. 2015;77(5):340-50. | Describes general patient preferences for service delivery |
|  | Neuhausen, Katherine, Grumbach, Kevin, Bazemore, Andrew, Phillips, Robert L. Integrating community health centers into organized delivery systems can improve access to subspecialty care. Health affairs (Project Hope) 2012; 31(8) 1708-16 | Describes methods of accessing specialist care rather than integrating services |
|  | Noble DJ, Greenhalgh T, Casalino LP. Improving population health one person at a time? Accountable care organisations: Perceptions of population health-a qualitative interview study. BMJ Open. 2014;4 (4) (no pagination)(e004665). | Perceptions of meaning of terms |
|  | Nugus P, Carroll K, Hewett DG, Short A, Forero R, Braithwaite J. Integrated care in the emergency department: A complex adaptive systems perspective. Social Science and Medicine. 2010;71(11):1997-2004. | Evaluates a method |
|  | Ouimet MJ, Pineault R, Prud'Homme A, Provost S, Fournier M, Levesque JF. The impact of primary healthcare reform on equity of utilization of services in the province of Quebec: A 2003-2010 follow-up Interventions in primary health care to improve outcome and equity in health. International Journal for Equity in Health. 2015;14 (1) (no pagination)(139). | Associations between service use and socio-economic status |
|  | Ouwens MMMTJ, Hermens RRPMG, Termeer RAR, Vonk-Okhuijsen SY, Tjan-Heijnen VCG, Verhagen AFTM, et al. Quality of integrated care for patients with nonsmall cell lung cancer: variations and determinants of care. Cancer. 2007;110(8):1782-90. | Describes the quality of services provided |
|  | Pourat N, Hadler MW, Dixon B, Brindis C. One-stop shopping: efforts to integrate physical and behavioral health care in five California community health centers. Policy brief (UCLA Center for Health Policy Research). 2015(PB2015-1):1-11. | Description only |
|  | Rea H, Kenealy T, Horwood F, Sheridan N, Parsons M, Wemekamp B, et al. Integrated systems to improve care for very high intensity users of hospital emergency department and for long-term conditions in the community. The New Zealand medical journal. 2010;123(1320):76-85. | Description only |
|  | Silow-Carroll S, Lamphere J. State innovation models: early experiences and challenges of an initiative to advance broad health system reform. Issue brief (Commonwealth Fund). 2013;25:1-12. | Policy brief |
|  | Silversmith J, Reform MMAWGtAHC. Five payment models: the pros, the cons, the potential. Minnesota medicine. 2011;94(2):45-8. | Recommendations rather than data |
|  | Smith KA. An Evaluation of a Pilot Epilepsy Special Care Center: University of California, Los Angeles; 2011. | Dissertation |
|  | Solberg LI, Asche SE, Shortell SM, Gillies RR, Taylor N, Pawlson LG, et al. Is integration in large medical groups associated with quality? The American journal of managed care. 2009;15(6):e34-41. | Focus on models of physician care |
|  | Solberg LI, Crain AL, Sperl-Hillen JM, Hroscikoski MC, Engebretson KI, O'Connor PJ. Care quality and implementation of the chronic care model: a quantitative study. Annals of family medicine. 2006;4(4):310-6. | Focus on disease-specific outcomes |
|  | Stewart B, Allan S, Keane B, Marshall B, Ayling J, Luxford T. Palliative Care Partnership: a successful model of primary/secondary integration. The New Zealand medical journal. 2006;119(1242):U2235. | Description only |
|  | Stiefel FC, Huyse FJ, Sollner W, Slaets JPJ, Lyons JS, Latour CHM, et al. Operationalizing integrated care on a clinical level: the INTERMED project. The Medical clinics of North America. 2006;90(4):713-58. | Description only |
|  | Suter E, Hyman M, Oelke N. Measuring key integration outcomes: a case study of a large urban health center. Health care management review. 2007;32(3):226-35. | Reports progress towards a model |
|  | Tebest R, Mehnert T, Nordmann H, Stock S. "Pflegestutzpunkte": Care Support Centers in Germany. Where are We Heading? Results of the Evaluation of all 48 Care Support Centers in Baden-Wurttemberg. Gesundheitswesen. 2015;9:9. | Non-English |
|  | Thomassen J-P, Ahaus K, Van de Walle S. Developing and implementing a service charter for an integrated regional stroke service: an exploratory case study. BMC health services research. 2014;14:141. | Development of a service charter |
|  | Tracy CS, Bell SH, Nickell LA, Charles J, Upshur REG. The IMPACT clinic: innovative model of interprofessional primary care for elderly patients with complex health care needs. Canadian family physician Medecin de famille canadien. 2013;59(3):e148-55. | Description only |
|  | Tsiachristas A, Hipple-Walters B, Lemmens KMM, Nieboer AP, Rutten-van Molken MPMH. Towards integrated care for chronic conditions: Dutch policy developments to overcome the (financial) barriers. Health policy (Amsterdam, Netherlands). 2011;101(2):122-32. | Description only |
|  | van Gils RF, Groenewoud K, Boot CRL, Rustemeyer T, van Mechelen W, van der Valk PGM, et al. Process evaluation of an integrated, multidisciplinary intervention programme for hand eczema. Contact dermatitis. 2012;66(5):254-63. | Focus on clinical care |
|  | Van Mierlo LD, Meiland FJM, Van Hout HPJ, Droes R-M. Towards personalized integrated dementia care: a qualitative study into the implementation of different models of case management. BMC geriatrics. 2014;14:84. | Clinical models |
|  | Vickers KS, Ridgeway JL, Hathaway JC, Egginton JS, Kaderlik AB, Katzelnick DJ. Integration of mental health resources in a primary care setting leads to increased provider satisfaction and patient access. General hospital psychiatry. 2013;35(5):461-7. | Care expansion rather than integration |
|  | Wagner EH, Sandhu N, Coleman K, Phillips KE, Sugarman JR. Improving care coordination in primary care. Medical care. 2014;52(11 Suppl 4):S33-8. | Use of a checklist for care co-ordination |
|  | Weinreb L, Nicholson J, Williams V, Anthes F. Integrating behavioral health services for homeless mothers and children in primary care. The American journal of orthopsychiatry. 2007;77(1):142-52. | Little data of relevance |
|  | Williams MV, Li J, Hansen LO, Forth V, Budnitz T, Greenwald JL, et al. Project BOOST implementation: lessons learned. Southern medical journal. 2014;107(7):455-65. | Patient safety focus |

**Appendix S5 Completed quality appraisal checklists**

**Quality appraisal UK RCTs and other studies with comparator groups**

| First author & date | Potential for selection bias? | | Potential for performance bias? | Potential for detection bias? | Potential for attrition bias? | Potential reporting  Bias? | Other sources of bias (comment) |
| --- | --- | --- | --- | --- | --- | --- | --- |
|  | Random sequence generation. | Allocation concealment. | Blinding of participants and personnel | Blinding of outcome assessments | Incomplete outcome data assessments | Selective reporting. | . |
| Boyle 2012 | NA  Anonymised data set | NA | NA | N | N | N | Able to describe associations, not causal links |
| Clarkson 2011 | NA, re-analysis of data from an earlier trial |  |  |  |  |  |  |
| Cunningham 2008 | N cluster | N | Y | N/Y | N | N | Many staff failed to complete required documentation |
| Department of Health 2012 | NA | NA | Y | Y | Y | N | Service data from intervention sites matched to control areas |
| Gravelle 2007 | NA | NA | N | Y | N | N | Small sample so low power. Definition of high risk group may be problematic. Used matched controls from other practices in England. |
| Higginson 2014 | N | Y | Single blind | Y | N | N | Self-Reported primary outcome, short follow up |
| Huws 2008 | N | N | N (Not possible) | N(Admissions data) | NA | N | Small number of practices and nurses delivering intervention |
| Julian 2007 | Y Compares different PCTs | N | Not possible | Y | Y | Y  No discussion of study limitations | Some data self-reported diary, only 32% if eligible GPs used tool |
| Lyon 2006 | Y  Compares neighbouring practices | N | Y | Y | N  Admissions data | N | Comparative observational study |
| Roland 2012 | NA | NA | N (for hospital data) | N | N | N | Data comprise both a self-reported questionnaire and hospital routine data. Used matched controls from other areas. |
| Simmons 2014 | N | NA | NA (uses routine data) | N | N | N (only two outcomes) | Only one year of data for intervention |
| Sinclair 2006 | NA  Crossover design compares period of intervention to no intervention | NA | Not possible | N  Data mostly routinely collected patient data | N  Routine hospital data | N | The intervention period lasted only 12 weeks. |
| Steventon 2011 | N | NA | Not possible | N | N  Routine hospital data | N | Much of the data self-reported |
| Stokes 2016 | NA  Anonymised data from a dataset, propensity matching, no significant baseline differences | NA | NA | NA | N  Data from existing dataset | N | Used a diference-in-differences design. Multiple intervention start dates. |
| Waller 2007 | NA  All patients in given time frame, small sample, some baseline differences | NA | CD | CD | N  Retrospective data from case notes | CD  May have been other service changes in time period | Compares group of patients prior to introduction with a separate group of patients after introduction. |
| Wilberforce, 2016 | CD  Anonymised patient data randomly selected but no details | NA | Y | Y | Y  Data for 877 of 960 – no explanation for missing data | N | The study is described as observational with two data time points, but compares two forms of teams and conclusions are based on this comparison. |

**Y=yes N=no NR=not reported NA= not applicable CD= cannot determine**

**Table 11. Quality appraisals UK Before and after/cohort studies**

| First author & date | 1. Was the study question or objective clearly stated? | 2. Were eligibility/selection criteria for the study population pre-specified and clearly described? | 3. Were the participants in the study representative of those who would be eligible for the test/service/intervention in the general or clinical population of interest? | 4. Were all eligible participants that met the pre-specified entry criteria enrolled? | 5. Was the sample size sufficiently large to provide confidence in the findings? | 6. Was the test/service/intervention clearly described and delivered consistently across the study population? | 7. Were the outcome measures pre-specified, clearly defined, valid, reliable, and assessed consistently across all study participants? | 8. Were the people assessing the outcomes blinded to the participants' exposures/interventions? | 9. Was the loss to follow-up after baseline 20% or less? Were those lost to follow-up accounted for in the analysis? | 10. Did the statistical methods examine changes in outcome measures from before to after the intervention? Were statistical tests done that provided p values for the pre-to-post changes? | 11. Were outcome measures of interest taken multiple times before the intervention and multiple times after the intervention (i.e., did they use an interrupted time-series design)? | 12. If the intervention was conducted at a group level (e.g., a whole hospital, a community, etc.) did the statistical analysis take into account the use of individual-level data to determine effects at the group level? |
| --- | --- | --- | --- | --- | --- | --- | --- | --- | --- | --- | --- | --- |
| Addicott 2008 | Y | Y | Y | N | Y | Y | Y | N | NA | Y | N | CD |
| Ahmad 2007 | N | N | Y | CD | N | CD | N | N | NA | CD | N | N |
| Bakerly 2009 | Y | Y | Y | N | CD | Y | Y | N | Y | Y | N | NA |
| Beacon 2015 | Y | N | CD | CD | CD | Y | Y | N | NA | N | N | N |
| Boyle 2008 | Y | NA | Y | NA | Y | Y | Y | N | NA | Y | N | CD |
| Boyle 2012 | Y | NA | Y | NA | Y | Y | Y | N | NA | Y | N | CD |
| Choo 2014 | Y | NA | Y | NA | Y | Y | Y | N | NA | Y | N | CD |
| Coupe 2013 | N | NA | Y | NA | Y | CD | N | N | CD | N | N | NA |
| Dodd 2011 | Y | N | Y | N | N | Y | CD | N | CD | Y | N | CD |
| Graffy 2008 | Y | Y | Y | CD | N | Y | N | N | Y | N | N | N |
| Ham 2010 | Y | Y | Y | CD | Y | N | N | CD | CD | N | N | N  Overview of work at a number of sites |
| Harris 2013 | Y | NA | NA | NA | NA | NA | NA | NA | NA | NA | NA | NA |
| Hawthorne 2009 | Y | NA | NA | NA | Y | CD | CD | N | NA | N | N | NA |
| Hockley 2010 | Y | Y | Y | N | CD | N | Y | N | NA | Y | N | CD |
| Jha 2007 | Y | CD | Y | N | N | CD | Y | N | NA | Y | N | N |
| Johnstone 2012 | Y | Y | Y | N | Y | CD | Y | N | NA | N | N | NA |
| Kent 2006 | Y | NA | Y | NA | Y | CD | Y | N | NA | Y | N | CD |
| Letton 2013 | Y | CD | Y | CD | CD | CD | Y | N | NA | Y | N | CD |
| Levelt 2008 | Y | Y | Y | N | CD | N | Y | N | NA | Y | N | N |
| Mertes 2013 | Y | Y | Y | Y | Y | CD | Y | N | NA | Y | N | N |
| Ng 2014 | Y | CD | Y | Y | Y | CD | Y | N | NA | N | N | N |
| Paize 2007 | Y | Y | Y | CD | N | N | Y | N | NA | N | N | N |
| Pettie 2011 | Y | Y | Y | Y | Y | N | Y | N | NA | Y | N | N |
| Richings 2011 | Y | Y | Y | N | N | CD | Y | N | CD | N | N | N |
| Roberts 2010 | Y | Y | Y | Y | Y | CD | Y | N | NA | Y | N | N |
| Soljak 2013 | Y | Y | Y | CD | Y | CD | Y | Y | CD | Y | N | Y |
| Tucker 2012 | Y | NA | Y (patients) | NA | N | CD | Y | N | NA | N | N | NA |
| Windle 2009 | Y | Y | Y | N | Y | N | CD | N | Y | Y | N | Y |

Y=yes N=no NR=not reported NA= not applicable CD= cannot determine

**Table 12. Quality appraisal UK Cross-sectional studies**

| First author & date | 1. Was the research question clearly stated? | 2. Was the study population clearly specified and defined? | 3. Was the participation rate of eligible persons at least 50%? | 4. Were all the subjects selected or recruited from the same or similar populations (including the same time period)? Were inclusion and exclusion criteria for being in the study prespecified and applied uniformly to all participants? | 5. Was a sample size justification, power description, or variance and effect estimates provided? | 6. For exposures that can vary in amount or level, did the study examine different levels of the exposure as related to the outcome? | 7. Were the exposure measures (independent variables) clearly defined, valid, reliable, and implemented consistently across all study participants? | 8. Were the outcome measures (dependent variables) clearly defined, valid, reliable, and implemented consistently across all study participants? | 9. Were the outcome assessors blinded to the exposure status of participants? | 10. Were key potential confounding variables measured and adjusted statistically for their impact on the relationship between exposure(s) and outcome(s)? |
| --- | --- | --- | --- | --- | --- | --- | --- | --- | --- | --- |
| Lamb 2014 | Y | Y | Y 54% | Y | N | NA | NA | NA  Survey of staff | NA | NA Non comparative |
| MacLean 2008 | Y | N | N | N | N | NA | NA | NA  Staff survey | NA | NA Non comparative |
| Offredy 2008 | N | N | CD | CD | N | CD | CD | CD  Limited data | N | N |
| Pearson 2011 | Y | N | CD | CD | N | CD | CD | Y | CD | N |
| Roberts 2012 | Y | Y | Y | Y | N | NA | N | N | N | N |
| Rowlandson 2009 | Y | Y | All eligible | Y | N | NA | NA | Little data on main outcome -timing of referral | NA | NA  Non-comparative |
| Ryan 2007 | Y | Y | CD | Y | N | NA | NA | Potential for inconsistent recording of information | NA | NA  Non-comparative |
| Smith 2012 | N | N | CD | N | N | NA | NA | N  Missing data | NA | NA  Non-comparative |
| Tucker 2009 | Y | Y | Y (72%) | Y | NA All eligible | NA | NA Survey of views | N  No reporting of survey development | NA | NA  Non-comparative |

Y=yes N=no NR=not reported NA= not applicable CD= cannot determine

**Table 13. Quality appraisal Non-UK RCTs and other studies with comparator groups**

| First author & date | Potential for selection bias? | | Potential for performance bias? | Potential for detection bias? | Potential for attrition bias? | Potential reporting bias? | Other sources of bias (comment) |
| --- | --- | --- | --- | --- | --- | --- | --- |
|  | Random sequence generation. | Allocation concealment. | Blinding of participants and personnel | Blinding of outcome assessments | Incomplete outcome data assessments | Selective reporting. | . |
| Aiken 2006 | Y | Y | N | CD | N | N |  |
| Battersby 2007 | Y | NA | NA | CD | CD | Y | Some HCPs cared for patients in both groups  Variable patient selection |
| Beland 2006 | Y | Y | NA | Y | N | CD | Inadequate statistical power (N=1230 where N=1270 was calculated to detect differences of 25% hospital and 50% nursing home utilisation.  Some contamination likely. |
| Bird | N (dummy control) | N | NA | Y | NA | CD | Self-selected groups, cannot establish cause-effect. |
| Bird 2012 | N (dummy control) | N | NA | Y | NA | CD | As above |
| Boult | Y cluster | Y | NA | Y | Y | N | Inadequate statistical power  Incomplete follow up (frail elderly population) |
| Brannstrom 2014 | Y | N | NA | N | N | N | Small samples (N=36 in each group), in one setting. Intervention group higher mean age than control. |
| Brown 2012 | Y | Y | CD | Y | N | N | Inadequate statistical power |
| Callahan 2006 | Y cluster | Y | Until recruitment | Y | Y but not outcomes relevant to our review | N | Small samples (N=84 intervention, n=69 control), therefore inadequate statistical power. |
| Colla 2012/16 | CD | CD | CD | CD | N | N | High degree of heterogeneity |
| Counsell 2007/ 2009 | Y | Y | Researchers blinded | CD | N | N | Multiple outcome measures and testing |
| Dorr 2008 | N | N | N | N | CD | CD | Differences in referral rates among physicians |
| Ettner 2006 | N/A | N/A | N | CD | N | N | Patients form own control group  Self-reported data |
| Fagan 2010 | N/A | N | CD | N | Y | N | Lack of data from comparator practices |
| Farmer 2011 | Y | CD | CD | CD | Y | N | Self-reported data  Large drop-out rate |
| Gray 2010 Hogg 2009 | Y | CD | N | N | N | N | Small sample |
| Hajewski 2014 | N | N/A | N | N | Y | CD | Some data sets not available at follow up (incomplete data recording of complex data). |
| Hammar 2009 | Y | Y | Y (Low risk patients)  N (higher risk for researchers recruiting) | N | N | N | Higher number of participants recruited to intervention group than control. |
| Hebert 2010 | N | N | N | N | CD | N | Overall sample identified randomly. |
| Hildebrandt 2012 | N | N | N | N | CD | Y | Selective and limited reporting |
| Hullick 2016 | N | N | N | CD | CD | N | Selection of participants on basis of number of ED attendances. |
| Jack 2009 | Y | Partial | Y | CD | N | N | Sample may be younger than other studies  Self-reported data |
| Janse 2014 | N | N | N | CD | Y | N | Small sample size; large loss to follow up.  Large number of statistical tests carried out. |
| Martinussen 2012 | N | N | N | N | N | CD | Pre-test measures not carried out.  Course was voluntary.  Self-report measures. |
| McGregor  Katon 2010 | Y | NR | Personnel blinded, blinding of patients not possible | Y | Y | CD/Unclear |  |
| Morales-Asencio 2008 | N | N | N | N | CD | CD |  |
| Olsson 2009 | N | N | N | N | Y | CD |  |
| Parsons 2012 | Y (cluster) | N | N | N | CD | CD | Practices randomised before participants identified |
| Paulus 2008 | N | N | N | N | NA | CD | Or NA for reporting bias? |
| Rosenheck 2016 | Y (cluster) | N | CD (blinding of patients not possible) | Y | CD | CD | Practices randomised before participants identified. |
| Sahlen 2015 | Y | NR | N | N | CD | N | Described as ‘open evaluation’. Separate methods/protocol paper lists outcomes |
| Salmon 2012 | N | NA | NA | NA | NA | CD | Study uses mainly administrative data |
| Stampa 2014 | N | N | N | N | CD | CD |  |
| Stewart 2010 | N | N | N | N | N | CD |  |
| Taylor 2013 | N | N | NA | N | NA | N | Small number of outcomes |
| Theodoridou 2015 | Y | Y | N (not possible) | N | N | CD | Allocation independent of trial personnel |
| van der Marck 2013 | N | N | CD | CD (wording unclear) | Y | CD |  |
| van Gils 2013 | Y | Y | N (not possible) | Y | CD | CD |  |
| Wennberg 2010 | Y | y | Y | CD | CD | N |  |

Y=yes N=no NR=not reported NA= not applicable CD= cannot determine

**Table 14. Quality appraisal systematic reviews**

|  | Were the research question and inclusion criteria specified a priori? | Were there at least two data extractors and was there a procedure for disagreements? | Were at least two databases searched? Did the report include years, databases, key words. Was there consultation and reference list checking? | Did the review include a search for grey literature? | Was a list of included and excluded studies provided? | Were the characteristics of included studies provided in an aggregated form such as a table? | Was the quality of included studies assessed? | Was the quality of included studies accounted for in the conclusions? | Were the methods used to combine data appropriately? Do the authors refer to the extent of heterogeneity? | Was the likelihood of publication bias assessed? | Was the source of support included for the review and each included study? |
| --- | --- | --- | --- | --- | --- | --- | --- | --- | --- | --- | --- |
| Alexander 2012 | Y | CD | N | N | N | N | Y | N | N | N | N |
| Allen 2008  2009 | Y | CD | Y | Y | Y | Y | Y | Y | Y | N | N |
| Beland 2011 | Y | Y | Y | CD | N | Y | Y | N | N | N | N |
| Belanger 2008 | Y | CD | Y | N | N | Y | CD | N | N | N | N |
| Best 2012 | Y | Y | Y | Y | Y | Y | CD | N | N | N | N |
| Boult 2009 | Y | Y | N | CD | N | Y | CD | N | N | N | N |
| Cameron 2014 | Y | Y | N | CD | N | N | Y | N | N | N | N |
| Davies 2011 | Y | Y | Y | Y | N | Y | Y | Y | Y | N | N |
| De Bruin 2012 | CD | Y | Y | N | N | Y | Y | Y | Y | N | N |
| Eklund 2009 | CD | Y | N | N | N | Y | Y | Y | Y | N | N |
| Footman 2014 | Y | Y | Y | Y | Y | Y | Y | Y | Y | N | N |
| Huntley 2013 | Y | Y | Y | Y | Y | Y | Y | Y | Y | N | N |
| Hussain 2014 | CD | CD | N | N | N | Y | Y | Y | Y | N | N |
| Jackson 2013 | Y | Y | N | N | Y | Y | Y | Y | Y | N | N |
| Johansson 2010 | Y | U | N | N | N | N | N | N | NA | N | N |
| Kammerlander 2010 | Y | Y | Y | N | Y | Y | N | N | Y | N | N |
| Kinley 2013 | Y | Y | Y | Y | N | N | Y | Y | Y | N | N |
| Kuhlmann 2010 | Y | Y | Y | N | N | Y | CD | N | Y | N | N |
| Laver 2014 | Y | Y | Y | N | N | Y | Y | Y | Y | N | N |
| Loader 2008 | Y | CD | CD | CD | N | N | N | N | CD | N | N |
| Low 2011 | Y | Y | Y | N | N | N | N | Y | N | N | N |
| MacAdam 2008 | Y | CD | Y | Y | N | Y | N | N | Y | N | N |
| Mackie 2016 | N | CD | CD | N | N | N | Y | Y | N | N | N |
| Martinez-Gonzalez 2014 | Y | Y | Y | N | Y | Y | Y | Y | N | N | N |
| Maslin-Prothero 2010 | Y | CD | N | N | N | N | N | N | N | N | N |
| Mason 2015 | Y | Y | Y | Y | N | N | N | N | N | N | N |
| McConnell 2013 | Y | CD | Y | Y | N | N | Y | Y | N | N | N |
| Myors 2013 | N | CD | N | CD | N | Y | N | N | N | N | N |
| Nicholson 2013 | CD | Y | Y | Y | N | Y | Y | N | N | N | N |
| Stewart 2013 | Y | CD | Y | Y | N | Y | N | N | N | N | N |
| Stokes 2015 | Y | Y | Y | N | Y | Y | Y | Y | Y | Y | N |
| Suter et al. 2009 | N | CD | CD | N | N | N | Y | N | N | N | N |
| Tieman 2006 | Y | Y | Y | Y | Y | N | Y | Y | N | N | N |
| Trivedi 2013 | Y | Y | Y | N | N | N | Y | Y | N | N | N |
| Xyrichis 2008 | y | CD | Y | N | N | N | N | N | NA | N | N |

Y=Yes N=No CD = Cannot determine NA=not applicable
