# Supplementary material for: The effects of integrated care: a systematic review of UK and international evidence
Source: BMC Health Serv Res. 2018 May 10;18:350. doi: 10.1186/s12913-018-3161-3 (PMC5946491; doi:10.1186/s12913-018-3161-3)
Supplement: Supplementary file 2 — Table S1. Summary of studies and effect for each outcome (DOCX 335 kb) [file 12913_2018_3161_MOESM2_ESM.docx]

**Table S1. Summary of studies and effect for each outcome**

*Note: studies highlighted in grey are those from the UK of comparative design*

|  | Type of evidence and rating | | | | | | | | |
| --- | --- | --- | --- | --- | --- | --- | --- | --- | --- |
| Outcome | UK studies | Strength of evidence rating | Systematic reviews | Strength of evidence rating | Non-UK comparator studies | Strength of evidence rating | Non-UK other studies | Strength of evidence rating | Overall rating |
| **Resource usage** |  |  |  |  |  |  |  |  |  |
| Number of clinician contacts + | +[30]  -[73] | Inconsistent evidence |  |  | + +[140, 150]  - - -[80, 128, 139] | Inconsistent evidence | -[171] | iii Very limited evidence | **Inconsistent evidence** |
| GP appointments | ±[44]  - -[49] [73] | Inconsistent evidence |  |  |  |  |  |  | **Inconsistent UK only evidence** |
| Length of stay | +[55]  ± ± [30, 71]  ± ± ±[20, 47, 52]  - - - - [40, 49, 67] [22]  - - - - - - - - - - [20, 26, 32, 35, 45, 51, 54, 57, 58, 73] | Inconsistent evidence | - - - - - - [80, 90, 93, 94, 102, 104]  ± ± ± ±.[91, 97, 99, 113] | Inconsistent evidence | - - [120, 138]  ± ±.[143, 161] | Inconsistent evidence | +[183]  - -[165, 172] | Inconsistent evidence | **Inconsistent evidence indicating no difference or reduced length of stay.**  One weak UK-only study reporting an increase. |
| Unscheduled admissions | + + + [60, 67, 68]  + + +[29, 31, 60]  ± ±[32, 64]  ± ± [34, 66]  - - - - - - - [23, 25, 26, 33, 40, 52, 58] | Inconsistent evidence | ± ± [90, 99]-[113] | Inconsistent evidence | - [158] | Very limited evidence | -[165] | iii Very limited evidence | **Inconsistent evidence** UK only evidence reporting and increase. |
| Admissions/  Number of inpatients | +[67]  + + + + [28, 29, 68, 72]  ±[30]  ±[66]  - - - -[31, 49, 60, 63]  - - - - - [25, 27, 32, 57, 69] | Inconsistent evidence | - - - -[95, 99, 102, 113]  ±[92] | Stronger evidence | ± ± ± ± ± ±[123, 128, 132, 133, 150, 158]  - - - - - - - - -[117, 121, 122, 127, 129, 141, 143, 144, 152] | Inconsistent evidence | ±[183]  - - [165, 173] | Inconsistent evidence | **Inconsistent evidence**  UK only evidence of an increase. |
| Re-admission | +[68]  + [55, 68]  ± ± ± [22, 24, 40] | Inconsistent evidence | - - [93, 102]  ±[104] | Inconsistent evidence | ± ± ± ± ± ± ±[123-125, 131, 132, 138, 140, 143] | i Stronger evidence |  |  | **Inconsistent evidence.** Stronger international evidence of no significant effect.  UK only evidence of an increase. |
| Emergency and urgent care attendance | - -[52, 73]  ± [60] | Inconsistent evidence | -[92]  ±[119] | Inconsistent | + + [133, 140]  ± ± ± ± ± ± [80, 116, 123, 139, 150, 161]  - - - - - [121, 122, 129, 132, 159] | Inconsistent evidence | -[166] | iii Very limited evidence | **Inconsistent evidence**  Little evidence indicating an increase in attendance at emergency and urgent care |
| Out-patient appointments | - - [60, 67]  - - [44, 53] | i Stronger evidence |  |  | ±[139] | iii Very limited evidence | ±[183] | iii Very limited evidence | **Inconsistent evidence.** Stronger UK evidence of a reduction. |
| Prescribing | ± [48]  -[71] | Inconsistent evidence | -[80] | iii Very limited evidence |  |  | -[167] | iii Very limited evidence | **iii Very limited evidence**  No evidence of an increase |
| Access to other resources |  |  | + (UK only) [83] | iii Very limited evidence |  |  | +[166] | iii Very limited evidence | **iii Very limited evidence** |
| **Quality of care** |  |  |  |  |  |  |  |  |  |
| Perceived quality | + + + +[31, 50, 58, 69] | i Stronger evidence of staff perceived increase | + + + [85, 87, 108]  ± [104] | i Stronger evidence, staff and patients report | + + [123, 124]  ±[148] | Inconsistent evidence |  |  | **i Stronger evidence of perceived improved quality of care.** |
| Quality standards |  |  |  |  | + +[137, 157]  ±[135] | Inconsistent evidence |  |  | **Inconsistent evidence** |
| Patient satisfaction | + [44]  + + + + + + ++[21, 23, 29, 32, 37, 52, 61, 69] ±[64]  - [60] | i Stronger evidence | + + + + + + + + + + +[13, 82, 85, 86, 92, 99, 102, 110, 111, 114, 185] | i Stronger evidence | + + + +[119, 136, 150, 159]  ± ± [131, 134] | Inconsistent evidence |  |  | **i Stronger evidence of improved patient satisfaction.** |
| Patient preferences met | + + +[20, 39, 188] | ii Weaker evidence |  |  |  |  |  |  | **ii UK only weaker evidence of a positive effect.** |
| Time spent in emergency and urgent care departments | ±[30] | iii Very limited evidence | - | iii Very limited evidence | -[143] | iii Very limited evidence |  |  | **iii Very limited evidence** |
| Number incidents/complaints | - -[25, 57] | iii Very limited evidence |  |  |  |  |  |  | **iii Very limited evidence** |
| Length of wait (contact, diagnosis, investigation, treatment) | - [49]  - - - - [27, 41, 61, 71] | i Stronger evidence | ±[94]  -[103] | Inconsistent evidence | ±[125]  - [118] | Inconsistent evidence | + [183]- [180] | Inconsistent evidence | **Inconsistent evidence** |
| Access to services | + + + + + [35, 41, 59, 72, 73] | i Stronger evidence | + +[76, 104] | ii Weaker evidence | + + + [117, 123, 124, 126] | i Stronger evidence of improved access | + + [167,179] | iii Very limited evidence | **i Stronger evidence of improved access** |
| Unmet need identified |  |  | +[110] | iii Very limited evidence | +[136] | iii Very limited evidence |  |  | **iii Very limited evidence** |
| **Staff work experience** |  |  | + + (UK only) [92, 103] | i Weaker evidence | ±[148] | iii Very limited evidence |  |  | **Inconsistent evidence** |
| **System impact** |  |  |  |  |  |  |  |  |  |
| Cost of provision | + [72]  ± ± [28, 38]  ± ± ± [20, 29, 31]  - [22]  - - - - [23, 32, 58, 63] | Inconsistent evidence | - - - - [77, 80, 82, 85]  ± ± ± ± ± ± [92, 102, 104, 111, 113, 114] | Inconsistent evidence | + + [137, 142]  ± ± ± ± ± ± ± [117, 131, 139, 153, 157, 162, 163, 167]  - - - - - - - [118, 120, 121, 129, 130, 147, 149] | Inconsistent evidence | -[174] | iii Very limited evidence | **Inconsistent evidence** |
| Community care activity | + [72]  + [37]  - [23] | Inconsistent evidence | - [111]  +[104] | Inconsistent evidence | + + [117, 152]  ± [128]  - - [77, 80] | Inconsistent evidence | +[166] | iii Very limited evidence | **Inconsistent evidence** |
| Secondary care activity | - [23] | iii Very limited evidence | -[85]  ± ± [104, 114] | Inconsistent evidence | -[121] | iii Very limited evidence | -[167] | iii Very limited evidence | **Inconsistent evidence** |
| Healthcare utilisation |  |  | Unclear effect[86, 99] | Inconsistent evidence |  |  |  |  | **Inconsistent evidence** |
